# CanVaxKB: a web-based cancer vaccine knowledgebase

**DOI:** 10.1093/narcan/zcad060

**Published:** 2024-01-09

**Authors:** Eliyas Asfaw, Asiyah Yu Lin, Anthony Huffman, Siqi Li, Madison George, Chloe Darancou, Madison Kalter, Nader Wehbi, Davis Bartels, Elyse Fleck, Nancy Tran, Daniel Faghihnia, Kimberly Berke, Ronak Sutariya, Farah Reyal, Youssef Tammam, Bin Zhao, Edison Ong, Zuoshuang Xiang, Virginia He, Justin Song, Andrey I Seleznev, Jinjing Guo, Yuanyi Pan, Jie Zheng, Yongqun He

**Affiliations:** College of Literature, Science, and the Arts, University of Michigan, Ann Arbor, MI 48109, USA; School of Medicine, University of Michigan, Ann Arbor, MI 48109, USA; Unit for Laboratory Animal Medicine, University of Michigan Medical School, Ann Arbor, MI 48109, USA; National Institutes of Health, 9000 Rockville Pike, Bethesda, MD 20892, USA; Department of Computational Medicine and Bioinformatics, University of Michigan, Ann Arbor, MI 48109, USA; College of Literature, Science, and the Arts, University of Michigan, Ann Arbor, MI 48109, USA; College of Literature, Science, and the Arts, University of Michigan, Ann Arbor, MI 48109, USA; College of Literature, Science, and the Arts, University of Michigan, Ann Arbor, MI 48109, USA; College of Literature, Science, and the Arts, University of Michigan, Ann Arbor, MI 48109, USA; College of Literature, Science, and the Arts, University of Michigan, Ann Arbor, MI 48109, USA; College of Electrical Engineering and Computer Science, University of Michigan, Ann Arbor, MI 48109, USA; College of Literature, Science, and the Arts, University of Michigan, Ann Arbor, MI 48109, USA; College of Literature, Science, and the Arts, University of Michigan, Ann Arbor, MI 48109, USA; College of Literature, Science, and the Arts, University of Michigan, Ann Arbor, MI 48109, USA; College of Literature, Science, and the Arts, University of Michigan, Ann Arbor, MI 48109, USA; College of Literature, Science, and the Arts, University of Michigan, Ann Arbor, MI 48109, USA; Department of Chemical, Biochemical and Environmental Engineering, University of Maryland, Baltimore County, Baltimore, MD 21250, USA; Department of Chemical, Biochemical and Environmental Engineering, University of Maryland, Baltimore County, Baltimore, MD 21250, USA; Unit for Laboratory Animal Medicine, University of Michigan Medical School, Ann Arbor, MI 48109, USA; Department of Computational Medicine and Bioinformatics, University of Michigan, Ann Arbor, MI 48109, USA; Unit for Laboratory Animal Medicine, University of Michigan Medical School, Ann Arbor, MI 48109, USA; The College of Brown University, Brown University, Providence, RI 02912, USA; College of Electrical Engineering and Computer Science, University of Michigan, Ann Arbor, MI 48109, USA; Dietrich School of Arts and Sciences, University of Pittsburgh, Pittsburgh, PA 15260, USA; Unit for Laboratory Animal Medicine, University of Michigan Medical School, Ann Arbor, MI 48109, USA; School of Information Management, Nanjing University, Nanjing, Jiangsu 210023, China; Unit for Laboratory Animal Medicine, University of Michigan Medical School, Ann Arbor, MI 48109, USA; School of Medicine, Guizhou University, Guiyang, Guizhou 550025, China; Unit for Laboratory Animal Medicine, University of Michigan Medical School, Ann Arbor, MI 48109, USA; Unit for Laboratory Animal Medicine, University of Michigan Medical School, Ann Arbor, MI 48109, USA; Department of Computational Medicine and Bioinformatics, University of Michigan, Ann Arbor, MI 48109, USA; Rogel Cancer Center, University of Michigan Medical School, Ann Arbor, MI 48109, USA

## Abstract

Cancer vaccines have been increasingly studied and developed to prevent or treat various types of cancers. To systematically survey and analyze different reported cancer vaccines, we developed CanVaxKB (https://violinet.org/canvaxkb), the first web-based cancer vaccine knowledgebase that compiles over 670 therapeutic or preventive cancer vaccines that have been experimentally verified to be effective at various stages. Vaccine construction and host response data are also included. These cancer vaccines are developed against various cancer types such as melanoma, hematological cancer, and prostate cancer. CanVaxKB has stored 263 genes or proteins that serve as cancer vaccine antigen genes, which we have collectively termed ‘canvaxgens’. Top three mostly used canvaxgens are PMEL, MLANA and CTAG1B, often targeting multiple cancer types. A total of 193 canvaxgens are also reported in cancer-related ONGene, Network of Cancer Genes and/or Sanger Cancer Gene Consensus databases. Enriched functional annotations and clusters of canvaxgens were identified and analyzed. User-friendly web interfaces are searchable for querying and comparing cancer vaccines. CanVaxKB cancer vaccines are also semantically represented by the community-based Vaccine Ontology to support data exchange. Overall, CanVaxKB is a timely and vital cancer vaccine source that facilitates efficient collection and analysis, further helping researchers and physicians to better understand cancer mechanisms.

## Graphical Abstract

**Graphical Abstract:**
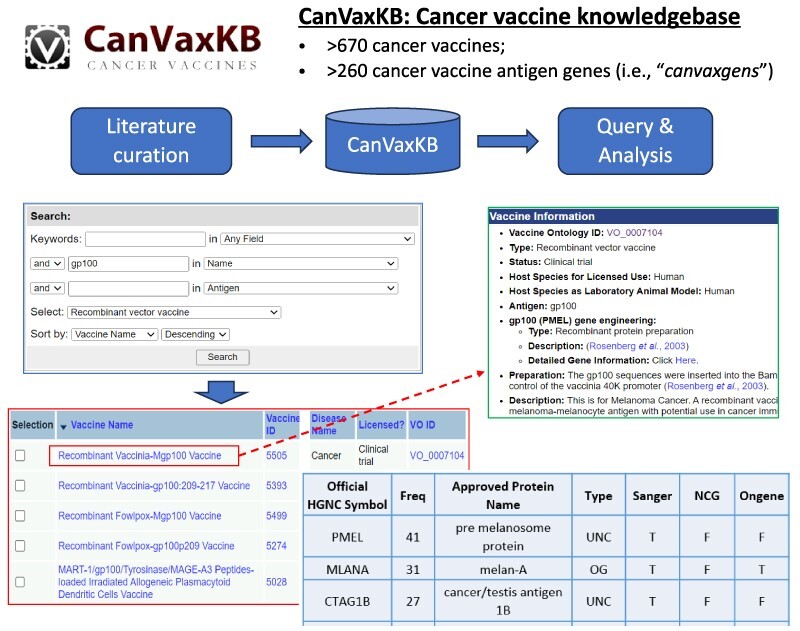

## Introduction

Cancer is the second leading cause of death after heart disease in the United States; its burden on public health and healthcare infrastructure is prominent (1). The GLOBOCAN 2020 estimated 19.3 million new cancer cases and almost 10.0 million cancer deaths in 2020 (2). The numbers are almost doubled compared to the statistics in the year 2000 (2,3). One of the ways to combat the consistent rise in cancer incidence is to put a heavier emphasis on the prevention and early detection since treatment is very likely insufficient (2).

Cancer can be treated by chemotherapy, radiation, surgery and vaccines (4). Cancer vaccines can be either preventive (cancer prevention vaccines) or therapeutic (cancer treatment vaccines) (4,5). Preventive vaccines block high-risk infections related with cancer-causing agents such as the human papillomavirus (HPV). The HPV vaccine has been shown to prevent >80% of cervical cancer and precancerous lesions (6). Therapeutic cancer vaccines aim to stimulate the patient’s adaptive immune system against specific tumor antigens to regain control over tumor growth, induce regression of established tumors and eradicate minimal residual disease (4). Therapeutic vaccines, such as dendritic cell vaccines, have been used to stimulate T-cell responses that have resulted in increased progression-free survival in patients with melanoma (7). Some existing infectious disease vaccines, such as the BCG vaccine, can also stimulate immunity against cancer; therefore, they have been repurposed and have been used in clinical trials for treating colon and lung cancers (8).

The importance of vaccines and vaccine-guided research was made clear due to the COVID-19 pandemic. The methods of immunoinformatics, reverse vaccinology and structural vaccinology have been applied to identify and optimize the SARS-CoV-2 S protein as the ideal candidate as the primary vaccine antigen (9–11). The development of the vaccine utilized both traditional vaccine platforms and the inclusion of the new messenger RNA (mRNA)-based platform. Ultimately, the first SARS-CoV-2 vaccines were licensed in the United States and the European Union in 10 months, using a mRNA-based vaccine format (12). This work was rewarded with a 2023 Nobel Prize for Physiology or Medicine. As such, the COVID-19 provides a proof of concept for successful, rapid development of efficacious vaccines.

Extensive research on cancer vaccines has been reported (13). Different cancer vaccine platforms exist for both preventive and therapeutic vaccines, including peptide vaccines, tumor antigen-expressing recombinant virus vaccines, DNA vaccines, autologous patient-derived immune cell vaccines and heterologous whole-cell vaccines derived from established human tumor cell lines (13,14). Our increasing understanding of the tumor microenvironment has made cancer antigen prediction more efficient, which can be targeted by vaccines (15). Cancer vaccines typically act in conjunction with T cells to immunologically target cancer cells. As a result of targeting a complicated signaling system, cancer vaccines tend to induce poor immunogenicity, and more research must be conducted into the mechanism of tumor immunology. Moreover, the vaccine antigens in cancer vaccines are altered host antigens that usually induce weaker responses in the host than the foreign antigens expressed by infectious pathogens (13). To increase immunogenicity, immune adjuvants and stimulants are frequently used (16). Current bottlenecks in cancer vaccine research include a lack of lucidity in cancer prevention or therapeutic mechanisms, difficulty in rational identification of cancer vaccine antigens and challenges in integrating different cancer vaccine information (16,17).

To better study cancer vaccines, aid cancer vaccine design and identify underlying cancer vaccine mechanisms, we developed CanVaxKB (https://violinet.org/canvaxkb), the first open data resource for a cancer vaccine knowledgebase. CanVaxKB is developed as an independent program under the umbrella of the Vaccine Information Online and Investigation Network (VIOLIN) vaccine database and analysis system (https://violinet.org) (18,19) in 2013. VIOLIN itself was founded in 2008 to serve as a primary knowledgebase and repository for all vaccines. CanVaxKB currently has data on 674 cancer vaccines for treatment or prevention that have been experimentally verified to be effective and/or are under clinical trials. CanVaxKB also stores hundreds of genes and proteins, which have been targeted specifically by cancer vaccines, which we have collectively termed ‘canvaxgens’. We hope that CanVaxKB will help researchers and physicians collaborate to understand cancer mechanisms better.

## Materials and methods

### CanVaxKB system and database design

The CanVaxKB system was built on two HP ProLiant DL380 G6 servers running the Red Hat Enterprise Linux operating system. A three-tier infrastructure was applied: a web user interface (presentation tier) to submit queries and display results, in addition to an application server using PHP/SQL (middle tier) against a MySQL relational database (data tier, database server). CanVaxKB is implemented as a subset of web-based semi-automatic VIOLIN literature mining and curation systems (18,19). This system allows data annotators to annotate and input annotated data to the VIOLIN database easily.

**Figure 1.**
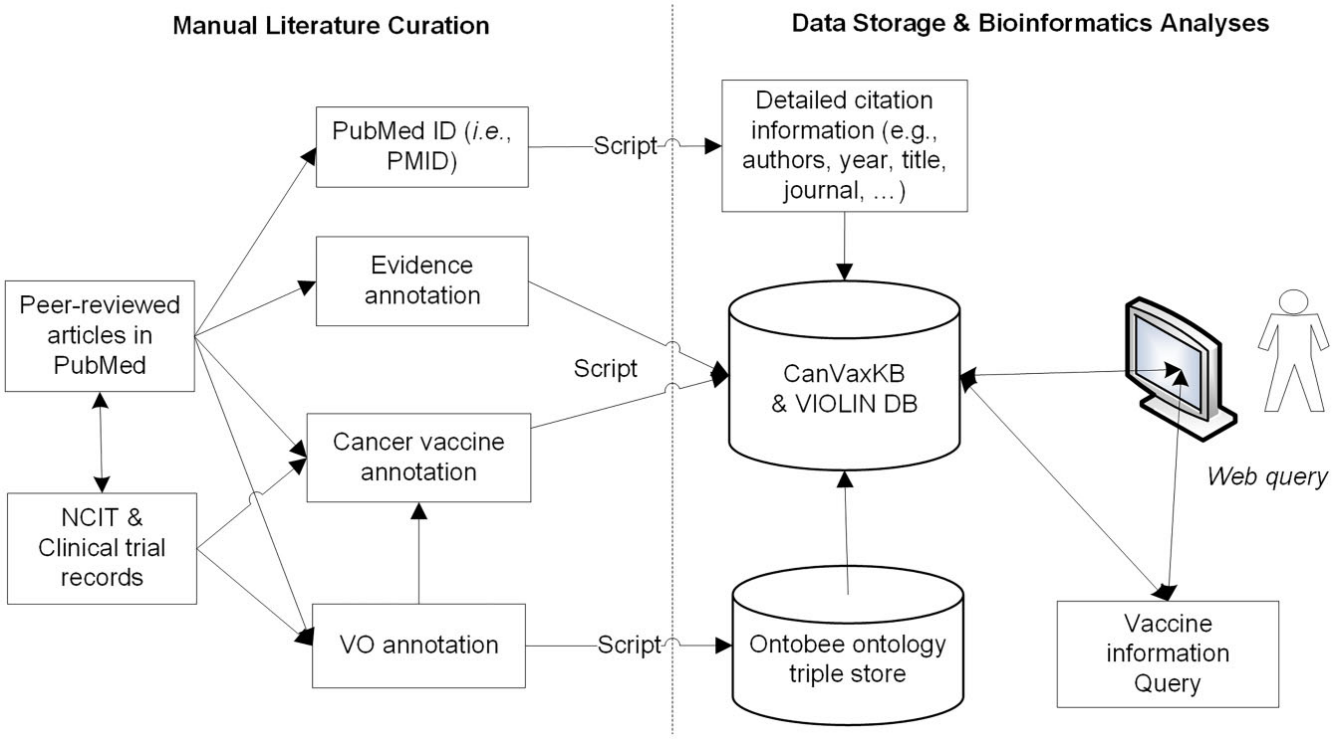
CanVaxKB web server design. Experimentally verified results of cancer vaccines and their related information such as antigens and host responses are manually annotated from peer-reviewed PubMed publications and stored into the CanVaxKB and VIOLIN databases. VO IDs assigned for vaccines and vaccine components were used for data sharing and data integration in CanVaxKB. An internal program was generated to extract comprehensive citation information using a PMID of a referenced paper. The NCBI IDs for the genes, proteins or nucleotides of protective antigens are identified and used by an internal script to retrieve detailed information (including sequences) about individual protective antigens.

Figure 1 illustrates the workflow of the CanVaxKB database design and implementation, which is similar to many other programs in the VIOLIN databases (18). Specifically, CanVaxKB manually collects annotated data from peer-reviewed articles and reliable websites of cancer vaccine clinical trials and federal resources. The genes involved in the cancer vaccines were identified and mapped to the pre-existing National Center for Biotechnology Information (NCBI) gene IDs. For each cancer vaccine, CanVaxKB collects the following information: the cancer vaccine name, Vaccine Ontology (VO) ID (if existent), preparation, vaccine formulation and reference. Detailed vaccine information, including antigen name, vaccine name, vaccine status (i.e. licensed, clinical trial or research), host animal model, vaccination route, protection protocol and efficacy, is also collected. We include additional information on specific components of the vaccine, including modifications of vaccine antigens or vectors. Further information about the vaccine, such as experimentally targeted cancer type, is included as part of the description or host response entries when available. For antigens matched to a gene, gene information is automatically added to the CanVaxKB database by an internal script to retrieve the NCBI gene, protein or nucleotide ID from the NCBI database. For each record, comprehensive citation information is included. The citation information is automatically retrieved from PubMed through an internal script using the PubMed ID (i.e. PMID) (19).

Cancer vaccines, vaccine formulations and vaccine-related information are represented using the standard VO, a community-based biomedical ontology in the area of vaccines and vaccination (20,21). A new VO ID was assigned if a cancer vaccine did not have one, which was then added to the VO as a new term. Updating of VO terms and IDs in batch was conducted via the use of the Ontorat tool (22) and Protege ontology editing tool (23).

### CanVaxKB data query and result display online

The CanVaxKB web interface includes keyword searches with 23 categories individually or combined. Some search categories include vaccine name, manufacturer, cancer antigen and vaccine preparation. The retrieved query results can be sorted by different vaccine types, vaccine names, pathogen names, disease names, VO IDs, or whether licensed or not. The user can also compare individual vaccines from the retrieved query results.

### Analysis of cancer vaccines and vaccine information stored in CanVaxKB

All cancer vaccines were analyzed after retrieving data from the front-end search interface or backend database queries using MySQL scripts. We analyzed the frequency of common antigens and vaccine components found within CanVaxKB. Analysis of the vaccines focused on addressing competence-based usage questions about the composition of vaccines in CanVaxKB after querying the database for information. Additionally, cancer vaccine antigen genes (i.e. canvaxgens) defined in CanVaxKB were compared with three large cancer gene databases: the Sanger Cancer Gene Consensus (CGC) database (24), the ONGene oncogene database (25) and the Network of Cancer Genes (NCG) database (26) for further validation (24). To identify common features in canvaxgens, the canvaxgens were then analyzed using the DAVID functional annotation tool (27). The functional analysis was performed on all cancer vaccine genes from a human background, with all genes annotated using NCBI gene IDs. Analysis results were considered significant if they had a false discovery rate (FDR) <0.05. Clusters were grouped using the default medium classification stringency.

## Results

### CanVaxKB cancer vaccine statistics

**Figure 2.**
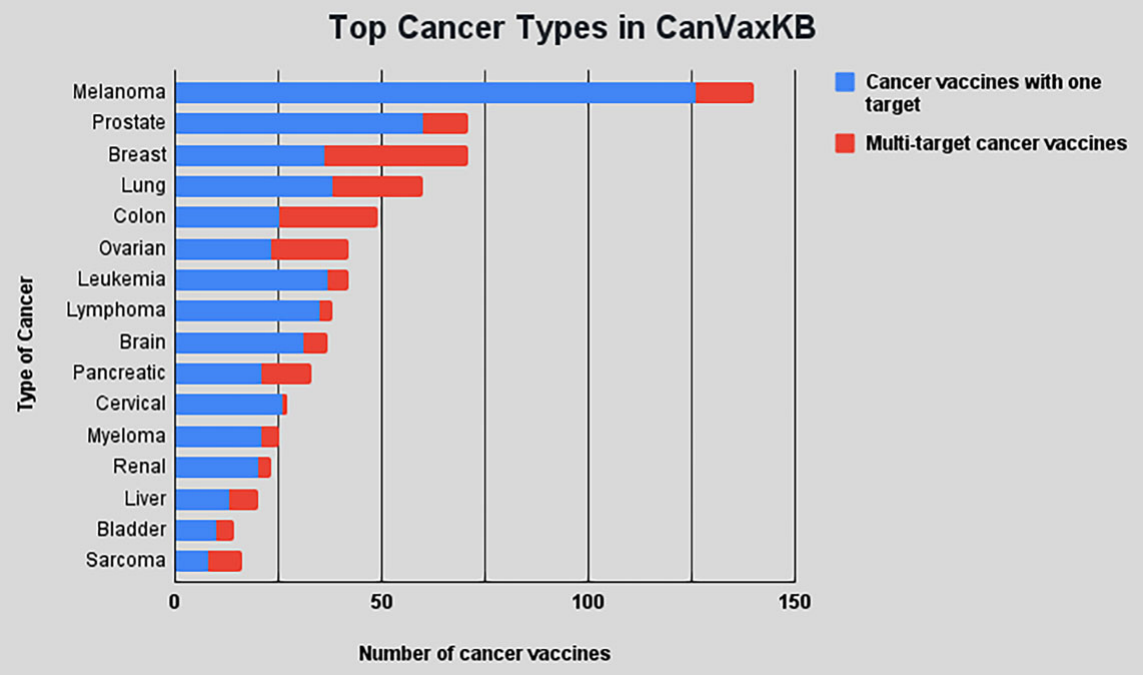
Top cancer types in CanVaxKB. Cancer types were assigned to cancer vaccines based on their vaccine reference. There are a total of 96 vaccines that target more than one cancer type.

As of August 2023, CanVaxKB has included 674 cancer vaccines. These 674 vaccines target 18 different cancer types and involve 263 canvaxgens in total. Each of the 263 canvaxgens contains one or more antigen targets. Some vaccines, like the ‘adenovirus encoding tyrosinase/MART-1/MAGE6-transduced autologous dendritic cell vaccine’, target three canvaxgens simultaneously. The top 3 cancer types in the CanVaxKB are melanoma, hematological cancers (lymphoma, leukemia, myeloma) and prostate cancer, which are associated with 140, 93 and 66 vaccines, respectively (Figure 2). Overall, a sixth of the cancer vaccines in the database have been utilized in treating or preventing at least two types of cancers. Breast cancer vaccines, while being the fifth most prevalent cancer vaccine in CanVaxKB, contain the largest fraction of multi-target cancer vaccines (∼50%), closely followed by colon cancer (∼49%) (Figure 2). Moreover, 157 cancer vaccines in CanVaxKB are currently being investigated in various stages of clinical trials, mostly earlier phase trials.

**Figure 3.**
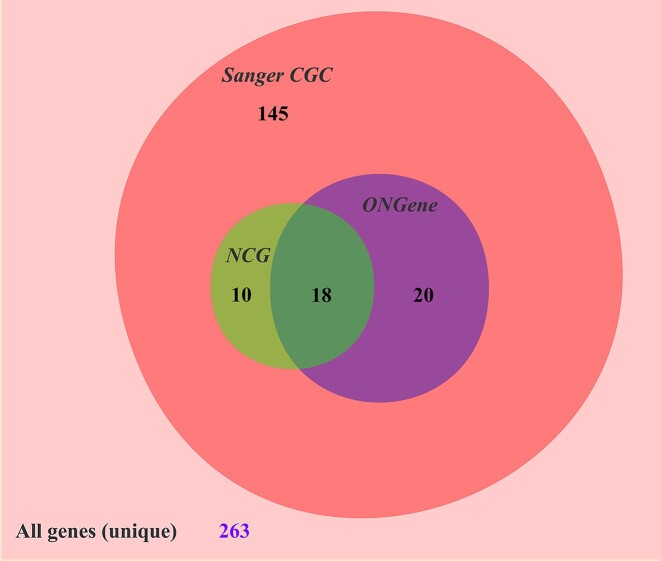
Comparison of CanVaxKB canvaxgens with cancer-related genes in other databases. A total of 193 genes from the ONGene oncogene database, the NCG database and Sanger CGC database are shared with the genes found in CanVaxKB. There are a total of 70 genes unique to CanVaxKB.

Out of 263 canvaxgens, 70 are uniquely found within CanVaxKB, and the remaining 71% (193/263) genes are also located in the Sanger CGC database, the ONGene database and/or the NCG database (Figure 3 and Supplementary Table S1). The Sanger CGC database (Tier 1) contains 719 genes with documented evidence that their mutations changed the activity of gene products in a way that promotes oncogenic transformation. The ONGene database contains 803 oncogenes. The NCG 7.1 database contains >2300 cancer genes and tumor suppressor/oncogene annotations. Our analysis found that many canvaxgens are indeed related to cancer oncogenic transformation or suppression.

### CanVaxKB data sharing and display

The VO is a community-based ontology that logically represents the terms of vaccines, vaccine components (such as vaccine antigens, adjuants, and recombinant vaccine vectors), vaccination, vaccine-induced immune responses and the relations among these terms (20,21). All the CanVaxKB cancer vaccines have been ontologically represented in VO (Supplementary File S1).

**Figure 4.**
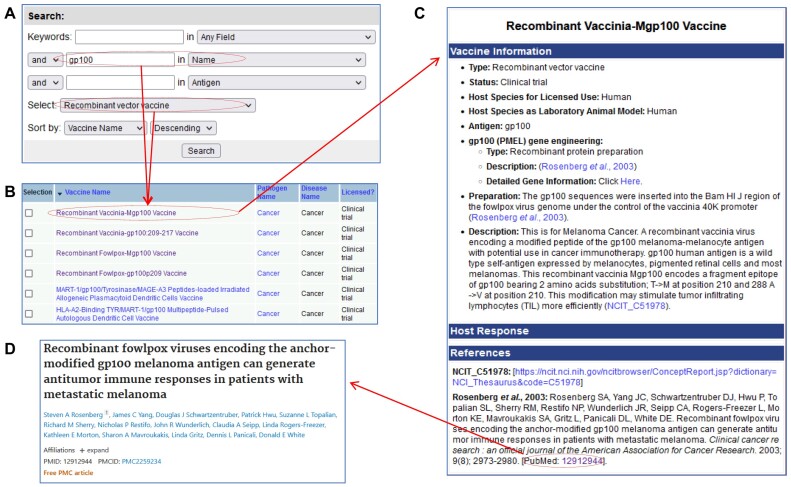
CanVaxKB database search. (**A**) Search for antigen ‘gp100’ and select ‘recombinant vector vaccine’. (**B**) Eleven gp100 cancer DNA vaccines were identified. (**C**) The details of a DNA vaccine by clicking on the vaccine name in panel (B). (**D**) A PubMed article reference detail was shown after clicking on the PubMed ID in panel (C).

The CanVaxKB provides user-friendly web interfaces for users to query and analyze the data stored in the CanVaxKB database. Figure 4 shows an example use of the query to search for specific vaccines by type, name or antigens. All vaccines can be queried through the use of three data fields for specific annotations and sorted via the appropriate vaccine type (Figure 4A). Once the search is completed, a table listing all vaccines can be brought up (Figure 4B). The vaccine names can be clicked on to see the full information of that vaccine in the database (Figure 4C). One can then use external references in CanVaxKB for more detailed information about the cancer vaccine (Figure 4D). Alternatively, multiple vaccines can be selected to compare to any other vaccines shown in the table.

### CanVaxKB top-ranked cancer vaccine antigens

Melanoma vaccines (*N* = 21%) have the largest share of cancer vaccines in CanVaxKB (Figure 2), utilizing 28 distinct antigens including the two most prevalent antigens in the database. Table 1 lists the top 10 most common canvaxgen proteins. The top 1 most common antigen in CanVaxKB is a premelanosome protein (PMEL) used in 41 cancer vaccines. The second most common is an oncogene MLANA (protein melan-A), a transmembrane protein routinely expressed in malignant melanoma (28), which is used in 31 vaccines in total. Altogether, melanoma vaccines account for about 140 vaccines found with CanVaxKB, of which nearly 125 target melanoma primarily, while the rest are also used in many other cancer types. Cancer/testis antigen 1B (CTAG1B), also known as New York esophageal squamous cell carcinoma 1, is the third most common antigen in CanVaxKB, which targets multiple types of cancers. Furthermore, human epidermal growth factor receptor-2 (HER-2) is the most common antigen used in breast cancer vaccines. HER-2 is used as a target because it is overexpressed in >20% of breast cancers (29). Finally, tumor protein p53 (TP53) is the most prevalent tumor suppressor in the CanVaxKB database.

**Table 1.** Top 10 frequently used canvaxgens in CanVaxKB and their presence in three oncogene databases

Official HGNC symbol	Frequency	Approved protein name	Type	Sanger	NCG	ONGene
PMEL	41	Premelanosome protein	UNC	T	F	F
MLANA	31	Melan-A	OG	T	F	T
CTAG1B	27	Cancer/testis antigen 1B	UNC	T	F	F
CEACAM5	25	CEA cell adhesion molecule 5	UNC	T	F	F
ERBB2	24	erb-b2 receptor tyrosine kinase 2	OG	T	T	T
MUC1	21	Mucin 1, cell surface associated	OG	T	T	T
KLK3 (PSA)	19	Kallikrein-related peptidase 3	UNC	T	T	F
MAGEA3	17	MAGE family member A3	UNC	T	F	F
TP53	15	Tumor protein p53	TSG	T	T	F
CD80	14	Cluster of differentiation 80 molecule	UNC	T	F	F

The approved protein name is the long form of the HGNC (HUGO Gene Nomenclature Committee) symbol. OG, oncogene; TSG, tumor suppressor gene; UNC, unclassified; T, true; F, false.

### CanVaxKB cancer vaccine formulation

Cancer vaccine constructs typically utilize synthetic constructs (307, ∼40%) or adjuvants (155/764, 20%) to help antigen presentation to elicit immune response. The synthetic constructs originate from either viruses or bacterial plasmids that have been modified for antigen presentation purposes. Furthermore, the vectors are modified from either a whole virus or a select set of non-infectious virus proteins to produce a virus-like particle. Either way, the use of a viral vector is one of the defining features of recombinant viral vaccines. Bacterial plasmids, in contrast, are used as the vectors for DNA vaccines. Altogether, there are 221 vaccines that utilize a viral vector and only 86 vaccines that use a bacterial plasmid. The two common adjuvants used for cancer vaccines are dendritic cells (108/764, 16.0%) and the granulocyte macrophage colony-stimulating factor (55/764, 7.1%). Both of these adjuvants occur naturally in humans as part of the adaptive immune response. Dendritic cells are commonly used in the construction of autologous vaccines, i.e. vaccines that utilize the antigen that is found with the host’s tumor (30).

### CanVaxKB canvaxgen gene set analysis

The canvaxgen gene set analysis showed a surprising few similarities for annotation information. DAVID mapped 191 canvaxgens as being human genes, while the remaining genes were identified as belonging to cancer-causing viruses (*N* = 25), lab mice (*N* = 24) and others (*N* = 31). Only the 191 human genes were selected for further functional analysis on the DAVID platform. DAVID identified 45 annotation clusters covering 215 statistically significant annotations (Supplementary Table S2). The only functional annotation found for the majority of canvaxgens was ‘protein binding’ (*N* = 159, FDR = 5.80−E07). Otherwise, the largest pluralities relate to cellular colocalization, namely the ‘plasma membrane’ (*N* = 100, FDR = 2.20E−13) or ‘extracellular region’ (*N* = 55, FDR = 4.00E−12). Many vaccines target antigens that must be accessible to the immune system to bind antibodies to, and as such are located either as part of the cell membrane or outside.

Our DAVID analysis also revealed 61 statistically significant clusters (Supplementary File S2). Table 2 shows the eight representative clusters, which represent many distinct functions or domains. The most common analysis for viral genes was ‘host–virus interaction’ (*N* = 22, FDR = 6.8E−4), which would correspond to viral genes known to cause cancer (e.g. HPV E6). The cluster of functional terms is related to ‘pathways of cancer’ (*N* = 30, FDR = 3.70E−08). This cluster contains the majority of statistically significant annotations (121 terms in total, of which 56 are statistically significant). This includes 36 annotations related to specific types of cancers, such as ‘melanoma’ (*N* = 9, FDR = 9.70E−05). One exception to this is a cluster containing ‘tumor antigen’ (*N* = 8, FDR = 9.6E−6) and ‘MAGE protein’ (*N* = 8, FDR = 3.00E−03), which are listed as the fourth separate cluster. This is likely related due to the high number of MAGE vaccines within CanVaxKB.

**Table 2.** Representative functional clusters of canvaxgens (i.e. cancer vaccine antigen genes) using DAVID

Functional cluster	Enrichment score	No. of canvaxgens	FDR	No. of terms in cluster
Signal	15.99	102	1.1E−20	4
Glycoprotein		106	7.1E−13	
Cytokine activity	8.51	5	2.87E−7	5
Plasma membrane	7.57	100	2.3E−13	11
Host cell receptor for virus entry	5.03	9	3.8E−4	4
Host–virus interaction		22	6.8E−4	
Toll-like receptor signaling pathway	4.68	12	3.8E−7	3
Positive regulation of MAP kinase activity	4.36	10	4.3E−8	3
Tumor antigen	3.88	8	9.6E−6	7
Pathways of cancer	2.64	30	3.7E−8	121

The first seven enriched functional clusters listed are the top 7 clusters based on enrichment score calculated by the DAVID tool, and the ‘pathways of cancer’ cluster is the 14th most significant out of 21.

## Discussion

To the best of our knowledge, CanVaxKB is the first web-based and publicly available knowledgebase of cancer vaccines. The CanVaxKB cancer vaccine knowledgebase stores the knowledge and related data of various cancer vaccines and their related information. The data contained in the CanVaxKB database are all manually curated. Well designed web query interfaces are also provided. The analysis of CanVaxKB data uncovers important statistics and enriched patterns from a well-organized database. It is also suggestive of potential gene targets for future cancer vaccines.

The leading cancer type targeted by vaccines in CanVaxKB is melanoma. Melanoma causes >55 000 deaths annually since it becomes life-threatening once it metastasizes to other parts of the body (31). The number of melanoma vaccines has consistently increased since the identification of melanoma-specific tumor antigens and increased understanding of immunological pathways (32). In CanVaxKB, peptide-based vaccines are commonly used in melanoma as they are straightforward and inexpensive. Still, the immune system can develop self-tolerance to these ‘self-antigens’ before they achieve their goal (31). The most frequently used antigen in CanVaxKB, PMEL/gp100, plays an important role in the proliferation of melanocytes and is a natural target of melanoma vaccines (33). When available, epitope-level data of PMEL/gp100 in CanVaxKB can allow easy vaccine design comparisons among multiple vaccines. Moreover, CanVaxKB has also shown that some cancer vaccines can be suitable targets for multiple types of cancers. Further in-depth analysis of how these vaccines interact with tumor environments of different cancer types and illicit immunogenicity is needed.

CanVaxKB is still under active development to address specific challenges. One challenge the CanVaxKB database will face is the consistent influx of new publications on cancer vaccines. Considering this, keeping up with new publications will be difficult. One option to aid in this process is through the use of natural language processing algorithms and pre-existing web tools to analyze abstracts and tests. One such example is the SciMiner tool, which will also allow for efficiently updating CanVaxKB with accurate information (34,35). Artificial intelligence (AI) and machine learning should be applied to the current CanVaxKB system, as AI will help to automatically encode new publications into the CanVaxKB database at a reasonable pace. However, the review and approval by domain experts are still needed for annotations made by machine. Another challenge CanVaxKB faces is that the search interface does not allow use of synonyms to query antigens. Currently, we standardize the name of all cancer antigens to a single antigen name that was decided during the curation process of CanVaxKB. However, the CanVaxKB statistics page contains a master list of all vaccines and vaccine antigens, including synonyms for each vaccine antigen. This list can be used to identify the proper name used to query for antigens within CanVaxKB. In the future, CanVaxKB database search interface backend should utilize the master list and enable the search via synonyms.

Future directions of CanVaxKB also include regularly updating the genes and gene fragments, mechanisms and clinical trials associated with cancer vaccines. Beyond regular maintenance, we plan to increase the number of cancer vaccines in the database as new vaccines are discovered and evaluated clinically. We are in the process of collecting and analyzing peptides and gene fragments used in cancer vaccine development. Such information can be used to support the rational design and development of custom cancer vaccines (36). Additionally, further work needs to be done to create uniformity in collecting, compiling and categorizing data for basic science and clinical research. Future refinement of the CanVaxKB database aims to determine mechanisms for generating new cancer vaccines and subsequent patient outcomes. The DAVID functional analysis is the first step in this process. It will require collaborations between basic scientists and clinicians to evaluate cancer vaccines in both settings. Ultimately, CanVaxKB will make current vaccine information analysis more effective and insightful. We welcome anyone who is interested in adding new vaccines or updating information to pre-existing cancer vaccines.

## Supplementary Material

zcad060_Supplemental_Files

## Data Availability

CanVaxKB website: https://violinet.org/canvaxkb/. The VO source is accessible at https://github.com/vaccineontology/VO and https://doi.org/10.5281/zenodo.10411622.
