# Supplementary material for: CanVaxKB: a web-based cancer vaccine knowledgebase
Source: NAR Cancer. 2024 Jan 9;6(1):zcad060. doi: 10.1093/narcan/zcad060 (PMC10776203; doi:10.1093/narcan/zcad060)
Supplement: zcad060_Supplemental_Files [file zcad060_supplemental_files.zip › Suppl_File2_Canvaxgens_DAVID_clusters.docx]

Supplemental File 2:

**The following is the raw output from our DAVID cluster analysis using the canvaxgens.**

---------

Annotation Cluster 1 Enrichment Score: 15.591865216613058

Category Term Count % PValue Genes List Total Pop Hits Pop Total Fold Enrichment Bonferroni Benjamini FDR

UP_KW_DOMAIN KW-0732~Signal 102 42.14876033057851 6.286592996135238E-22 3123, 3484, 7042, 7162, 2548, 354, 3598, 634, 6868, 8784, 7299, 4582, 6366, 4583, 4584, 4585, 768, 5788, 2719, 2717, 925, 6375, 94025, 5047, 3383, 1084, 6490, 7184, 4072, 1116, 2323, 1234, 2321, 54894, 6007, 10232, 1638, 8829, 4240, 8842, 3791, 2583, 1493, 2064, 2581, 7070, 6382, 780, 941, 942, 1001, 945, 4758, 7123, 3563, 3440, 3043, 2348, 51284, 57823, 2984, 11027, 958, 2902, 55, 7099, 7015, 2760, 7490, 727897, 965, 4153, 7422, 2250, 174, 282617, 11009, 2922, 1437, 1436, 3458, 3456, 3696, 7018, 1956, 1438, 4162, 8000, 5133, 11010, 2022, 3593, 3592, 3106, 1048, 3105, 54742, 1442, 54106, 7306, 1969, 1604 149 4363 14504 2.275707712967649 1.1944526692656952E-20 1.1944526692656952E-20 1.0687208093429905E-20

UP_SEQ_FEATURE CARBOHYD:N-linked (GlcNAc...) asparagine 89 36.77685950413223 3.336756014026076E-15 3123, 3563, 7042, 7162, 2548, 2348, 354, 51284, 57823, 2984, 2346, 3598, 952, 634, 11027, 958, 6868, 2902, 170482, 55, 7099, 8784, 7299, 4582, 4583, 6489, 4585, 2760, 727897, 965, 768, 5788, 2719, 2717, 94025, 7422, 8995, 3383, 1084, 2250, 6490, 174, 7184, 4072, 11009, 1437, 1436, 1116, 3458, 3456, 2323, 2321, 54894, 3696, 10232, 6528, 1638, 8829, 1956, 1438, 4162, 4240, 8000, 8842, 5133, 3791, 2583, 2022, 1493, 3593, 2064, 2581, 7070, 3592, 6382, 780, 3106, 1048, 3105, 54742, 941, 942, 1001, 945, 7306, 54106, 1969, 4758, 1604 188 4375 20543 2.2228899696048634 4.692912725090537E-12 4.701489223762741E-12 4.608060055370011E-12

UP_KW_PTM KW-1015~Disulfide bond 94 38.84297520661157 2.897722815409835E-14 7123, 7124, 3123, 3563, 3484, 3440, 7042, 7162, 2548, 2348, 354, 57823, 3598, 952, 634, 11027, 958, 959, 6868, 2902, 170482, 55, 7099, 8784, 4582, 6366, 4583, 4584, 6489, 4585, 2760, 727897, 965, 768, 207, 2719, 2717, 925, 5903, 6375, 4153, 94025, 7422, 5047, 8995, 1880, 3383, 6490, 174, 282617, 7184, 4072, 11009, 1437, 1436, 1116, 3456, 2323, 1234, 2321, 931, 54894, 3696, 7018, 10232, 6528, 8829, 1956, 1438, 4162, 4240, 8000, 5133, 3791, 2583, 2022, 1493, 3593, 2064, 2581, 7070, 3592, 780, 3106, 1048, 3105, 1442, 941, 942, 945, 7306, 54106, 1969, 1604 172 3816 14111 2.0209186534054897 6.664668816824815E-13 6.954534756983604E-13 5.79544563081967E-13

UP_KW_PTM KW-0325~Glycoprotein 106 43.80165289256198 7.058988868576336E-14 3123, 7042, 7162, 2548, 354, 3598, 634, 6868, 8784, 7299, 4582, 4583, 4584, 6489, 4585, 768, 5788, 2719, 2717, 925, 94025, 5047, 3265, 3383, 1084, 6490, 7184, 4072, 1116, 2323, 1234, 2321, 54894, 10232, 6528, 1638, 8829, 4240, 8842, 3791, 2583, 1493, 2064, 2581, 7070, 6382, 780, 941, 942, 1001, 945, 4758, 7123, 7124, 4893, 3563, 3440, 3043, 2348, 51284, 57823, 2346, 2984, 952, 11027, 958, 959, 2902, 170482, 55, 7099, 2760, 727897, 3728, 80312, 965, 207, 3845, 7422, 8995, 2250, 174, 11009, 1437, 1436, 3458, 3456, 3696, 7018, 1956, 1438, 4162, 8000, 5133, 7157, 2022, 3593, 3592, 3106, 1048, 3105, 54742, 54106, 7306, 1969, 1604 172 4740 14111 1.8346653910313022 1.624034240421679E-12 8.470786642291604E-13 7.058988868576336E-13

Annotation Cluster 2 Enrichment Score: 8.507227971865229

Category Term Count % PValue Genes List Total Pop Hits Pop Total Fold Enrichment Bonferroni Benjamini FDR

GOTERM_BP_DIRECT GO:0042531~positive regulation of tyrosine phosphorylation of STAT protein 12 4.958677685950414 3.497283787293143E-11 11009, 1437, 8784, 1436, 3458, 7124, 1442, 8995, 3440, 3593, 3592, 958 183 68 19414 18.721311475409838 7.25685623814698E-8 2.4189546195444238E-8 2.2883893581521468E-8

KEGG_PATHWAY hsa04060:Cytokine-cytokine receptor interaction 23 9.50413223140496 4.0349586249849027E-10 8784, 6375, 8743, 6366, 7124, 8995, 3563, 3440, 3593, 3592, 7042, 282617, 11009, 1437, 1436, 3458, 3456, 1234, 1442, 3598, 958, 959, 1438 128 297 8465 5.121396254208754 9.966348313295015E-8 4.9831739018563546E-8 3.187617313738073E-8

GOTERM_MF_DIRECT GO:0005125~cytokine activity 16 6.6115702479338845 6.309877294014026E-10 8743, 7422, 7124, 8995, 3440, 3593, 3592, 7042, 282617, 11009, 1437, 3458, 2247, 3456, 2323, 959 185 192 18945 8.533783783783784 2.88361340361476E-7 2.88361392336441E-7 2.8015855185422275E-7

UP_KW_MOLECULAR_FUNCTION KW-0202~Cytokine 15 6.198347107438017 2.9517998679035095E-8 6375, 8743, 6366, 7124, 8995, 3440, 3593, 3592, 282617, 11009, 1437, 3458, 3456, 2323, 959 130 196 11749 6.916601255886971 1.8596322172159674E-6 1.859633916779211E-6 1.7710799207421058E-6

INTERPRO IPR009079:Four-helical cytokine-like, core 8 3.3057851239669422 1.107088546091646E-6 11009, 1437, 3458, 3456, 2323, 1442, 3440, 3592 183 57 19144 14.682389032691017 4.7150878646173844E-4 4.7161972063504117E-4 4.5722756953584973E-4

Annotation Cluster 3 Enrichment Score: 7.571270313977156

Category Term Count % PValue Genes List Total Pop Hits Pop Total Fold Enrichment Bonferroni Benjamini FDR

GOTERM_CC_DIRECT GO:0005886~plasma membrane 100 41.32231404958678 2.221805132213744E-15 3123, 7162, 2548, 634, 10134, 6868, 8784, 4582, 4583, 4100, 4584, 4585, 2315, 367, 768, 5788, 2719, 925, 94025, 1880, 3265, 3383, 1084, 6490, 7184, 4193, 4072, 4904, 2323, 1234, 2321, 54894, 931, 6007, 10232, 6528, 1638, 8829, 8842, 3791, 1493, 2064, 7070, 6382, 780, 941, 942, 1001, 945, 10486, 4758, 6277, 4771, 7124, 4893, 3563, 2348, 51284, 57823, 2346, 2984, 952, 26872, 11027, 958, 959, 2902, 170482, 55, 7099, 8743, 7015, 23532, 6280, 727897, 3329, 3728, 965, 207, 3845, 8995, 2130, 1437, 1436, 3696, 7018, 1956, 1438, 4162, 8000, 5133, 11010, 2022, 3106, 1048, 3105, 54742, 54106, 1969, 1604 188 5333 20624 2.0570434588331983 7.371880883511039E-13 2.4587976796498765E-13 2.273647251965398E-13

UP_SEQ_FEATURE CARBOHYD:N-linked (GlcNAc...) asparagine 89 36.77685950413223 3.336756014026076E-15 3123, 3563, 7042, 7162, 2548, 2348, 354, 51284, 57823, 2984, 2346, 3598, 952, 634, 11027, 958, 6868, 2902, 170482, 55, 7099, 8784, 7299, 4582, 4583, 6489, 4585, 2760, 727897, 965, 768, 5788, 2719, 2717, 94025, 7422, 8995, 3383, 1084, 2250, 6490, 174, 7184, 4072, 11009, 1437, 1436, 1116, 3458, 3456, 2323, 2321, 54894, 3696, 10232, 6528, 1638, 8829, 1956, 1438, 4162, 4240, 8000, 8842, 5133, 3791, 2583, 2022, 1493, 3593, 2064, 2581, 7070, 3592, 6382, 780, 3106, 1048, 3105, 54742, 941, 942, 1001, 945, 7306, 54106, 1969, 4758, 1604 188 4375 20543 2.2228899696048634 4.692912725090537E-12 4.701489223762741E-12 4.608060055370011E-12

GOTERM_CC_DIRECT GO:0005887~integral component of plasma membrane 44 18.181818181818183 2.252943365697107E-12 7124, 1880, 3123, 6490, 3383, 7162, 1436, 2346, 1234, 26872, 2321, 931, 54894, 634, 6007, 10134, 11027, 958, 6868, 959, 2902, 1956, 1438, 7099, 8784, 4582, 8842, 3791, 8743, 4585, 1493, 2064, 7070, 6382, 780, 2315, 3106, 1048, 3105, 965, 5788, 945, 925, 1969 188 1438 20624 3.356671500014796 7.479878938454476E-10 1.4959543948228793E-10 1.3833072265380236E-10

UP_SEQ_FEATURE TOPO_DOM:Extracellular 59 24.380165289256198 3.807557958803041E-9 7124, 3123, 3563, 7162, 51284, 57823, 2984, 2346, 3598, 952, 634, 11027, 958, 959, 6868, 2902, 170482, 7099, 8784, 4582, 8743, 965, 768, 5788, 925, 94025, 8995, 1880, 3383, 1084, 4072, 1436, 2323, 1234, 2321, 931, 54894, 3696, 6528, 8829, 1956, 1438, 4162, 8842, 5133, 3791, 2022, 1493, 2064, 6382, 780, 3106, 3105, 941, 942, 1001, 945, 54106, 1969 188 2941 20543 2.192113335310757 5.364834750665182E-6 2.6824245819767425E-6 2.6291187705535E-6

UP_SEQ_FEATURE TOPO_DOM:Cytoplasmic 68 28.09917355371901 2.1020369526754154E-8 7124, 3123, 3563, 7162, 51284, 57823, 2984, 2346, 3598, 952, 634, 10134, 11027, 958, 959, 6868, 2902, 170482, 7099, 8784, 7299, 4582, 8743, 6489, 2315, 965, 768, 5788, 925, 94025, 8995, 1880, 3383, 1084, 6490, 4072, 1436, 51062, 2323, 1234, 2321, 931, 54894, 3696, 6528, 1638, 8829, 1956, 1438, 4162, 8842, 5133, 3791, 2583, 2022, 1493, 2064, 6382, 780, 3106, 3105, 941, 942, 1001, 945, 7306, 54106, 1969 188 3837 20543 1.9365250999506485 2.9617262416015144E-5 9.8725668877322E-6 9.676376772149162E-6

UP_SEQ_FEATURE TRANSMEM:Helical 82 33.88429752066116 2.5370610699837436E-7 7124, 3123, 3563, 3043, 7162, 445347, 2548, 2348, 51284, 57823, 2984, 2346, 598, 3598, 952, 26872, 634, 10134, 11027, 958, 959, 6868, 2902, 170482, 7099, 8784, 7299, 4582, 8743, 4584, 6489, 4585, 2315, 367, 965, 768, 5788, 925, 94025, 8995, 1880, 3383, 1084, 6490, 4072, 1436, 51062, 2323, 1234, 2321, 931, 54894, 3696, 6007, 6528, 1638, 8829, 1956, 1438, 140738, 4162, 8842, 5133, 3791, 7157, 11010, 2583, 2022, 1493, 2064, 6382, 780, 3106, 3105, 941, 942, 1001, 945, 7306, 54106, 1969, 4758 188 5374 20543 1.6673324675941692 3.574080646411293E-4 8.936797619017737E-5 8.759203344118875E-5

UP_KW_CELLULAR_COMPONENT KW-1003~Cell membrane 69 28.512396694214875 1.6886157663365986E-6 6277, 4771, 7124, 4893, 3123, 7162, 2348, 2984, 2346, 634, 11027, 958, 959, 2902, 170482, 55, 7099, 8784, 4582, 8743, 4585, 23532, 6280, 965, 768, 207, 5788, 2719, 925, 3845, 94025, 8995, 1880, 3265, 2130, 4072, 1436, 2323, 1234, 2321, 931, 54894, 3696, 6007, 10232, 6528, 8829, 1956, 1438, 8000, 8842, 5133, 3791, 2022, 1493, 2064, 7070, 780, 3106, 1048, 3105, 54742, 942, 1001, 945, 10486, 1969, 4758, 1604 182 3923 17708 1.7113108660393899 5.909985525609507E-5 2.9550775910890477E-5 2.701785226138558E-5

GOTERM_CC_DIRECT GO:0016021~integral component of membrane 73 30.165289256198346 1.365296944729555E-4 7124, 3123, 3563, 3043, 445347, 2548, 2348, 51284, 57823, 2984, 2346, 598, 3598, 952, 26872, 634, 10134, 958, 959, 6868, 2902, 170482, 55, 7099, 7299, 4582, 8743, 4584, 6489, 4585, 2315, 367, 965, 768, 5788, 925, 94025, 8995, 1880, 3383, 1084, 6490, 4072, 1436, 10953, 51062, 2323, 1234, 54894, 6007, 6528, 1638, 8829, 1956, 140738, 4162, 8842, 5133, 7157, 11010, 2022, 1493, 2064, 6382, 3106, 3105, 941, 942, 1001, 945, 7306, 54106, 4758 188 5394 20624 1.484659866360574 0.044318856191699174 0.003777321547085102 0.003492884683599778

UP_KW_DOMAIN KW-1133~Transmembrane helix 82 33.88429752066116 2.531030723541116E-4 7124, 3123, 3563, 3043, 7162, 445347, 2548, 2348, 51284, 57823, 2984, 2346, 598, 3598, 952, 26872, 634, 10134, 11027, 958, 959, 6868, 2902, 170482, 7099, 8784, 7299, 4582, 8743, 4584, 6489, 4585, 2315, 367, 965, 768, 5788, 925, 94025, 8995, 1880, 3383, 1084, 6490, 4072, 1436, 51062, 2323, 1234, 2321, 931, 54894, 3696, 6007, 6528, 1638, 8829, 1956, 1438, 140738, 4162, 8842, 5133, 3791, 7157, 11010, 2583, 2022, 1493, 2064, 6382, 780, 3106, 3105, 941, 942, 1001, 945, 7306, 54106, 1969, 4758 149 5844 14504 1.365856795703963 0.004798019611020665 0.0016029861249093735 0.0014342507433399657

UP_KW_DOMAIN KW-0812~Transmembrane 82 33.88429752066116 3.741199780324548E-4 7124, 3123, 3563, 3043, 7162, 445347, 2548, 2348, 51284, 57823, 2984, 2346, 598, 3598, 952, 26872, 634, 10134, 11027, 958, 959, 6868, 2902, 170482, 7099, 8784, 7299, 4582, 8743, 4584, 6489, 4585, 2315, 367, 965, 768, 5788, 925, 94025, 8995, 1880, 3383, 1084, 6490, 4072, 1436, 51062, 2323, 1234, 2321, 931, 54894, 3696, 6007, 6528, 1638, 8829, 1956, 1438, 140738, 4162, 8842, 5133, 3791, 7157, 11010, 2583, 2022, 1493, 2064, 6382, 780, 3106, 3105, 941, 942, 1001, 945, 7306, 54106, 1969, 4758 149 5905 14504 1.3517471827424148 0.007084396102861157 0.0017770698956541603 0.0015900099066379328

UP_KW_CELLULAR_COMPONENT KW-0472~Membrane 107 44.214876033057855 7.023632634687543E-4 3123, 7162, 2548, 598, 3598, 634, 10134, 6868, 8784, 7299, 4582, 4584, 6489, 4585, 2315, 367, 768, 5788, 2719, 925, 94025, 1880, 3265, 3383, 1084, 6490, 4072, 2323, 1234, 2321, 54894, 931, 6007, 10232, 6528, 1638, 8829, 4240, 8842, 3791, 2583, 1493, 2064, 7070, 6382, 780, 941, 942, 1001, 945, 10486, 4758, 6277, 4771, 7124, 4893, 3563, 3043, 445347, 2348, 51284, 53340, 57823, 2346, 2984, 952, 26872, 11027, 958, 959, 2902, 170482, 55, 7099, 8743, 23532, 6280, 3728, 965, 207, 10983, 3845, 5903, 8995, 2130, 51062, 1436, 10953, 3696, 1956, 1438, 140738, 4162, 8000, 5133, 7157, 11010, 2022, 3106, 1048, 3105, 54742, 7428, 54106, 7306, 1969, 1604 182 8234 17708 1.2643608516817164 0.02429144736203881 0.008194238073802134 0.007491874810333379

Annotation Cluster 4 Enrichment Score: 5.032715137829683

Category Term Count % PValue Genes List Total Pop Hits Pop Total Fold Enrichment Bonferroni Benjamini FDR

GOTERM_BP_DIRECT GO:0046718~viral entry into host cell 9 3.71900826446281 5.293977557592981E-6 983, 1234, 941, 942, 1969, 3383, 8829, 1604, 1956 183 102 19414 9.360655737704917 0.010924917363224318 4.0685197896316423E-4 3.848917757612971E-4

UP_KW_BIOLOGICAL_PROCESS KW-0945~Host-virus interaction 22 9.090909090909092 1.0732710711102534E-5 3791, 7157, 4343, 3592, 3383, 4193, 3329, 332, 3106, 3105, 983, 1234, 941, 942, 5698, 10134, 5788, 9978, 1969, 8829, 1604, 1956 125 665 11262 2.9806195488721805 7.40286865555162E-4 7.405570390660748E-4 6.761607747994595E-4

GOTERM_MF_DIRECT GO:0001618~virus receptor activity 8 3.3057851239669422 1.07420725659505E-5 983, 1234, 941, 942, 1969, 3383, 1604, 1956 185 78 18945 10.503118503118504 0.004897123329801745 6.944720365611749E-4 6.747168145145769E-4

UP_KW_MOLECULAR_FUNCTION KW-1183~Host cell receptor for virus entry 8 3.3057851239669422 1.2121597173398103E-5 983, 1234, 941, 942, 1969, 3383, 1604, 1956 130 71 11749 10.183315276273023 7.633737322629841E-4 2.5455354064136016E-4 2.4243194346796205E-4

Annotation Cluster 5 Enrichment Score: 4.681346560721425

Category Term Count % PValue Genes List Total Pop Hits Pop Total Fold Enrichment Bonferroni Benjamini FDR

KEGG_PATHWAY hsa04620:Toll-like receptor signaling pathway 12 4.958677685950414 3.8379481375823866E-7 7099, 51284, 3456, 7124, 941, 942, 207, 3440, 3593, 54106, 3592, 958 128 104 8465 7.630709134615385 9.479284405156108E-5 5.576312882252056E-6 3.567034151400101E-6

GOTERM_BP_DIRECT GO:1901224~positive regulation of NIK/NF-kappaB signaling 7 2.8925619834710745 5.401469985570723E-5 7099, 51284, 7124, 942, 3593, 54106, 1956 183 71 19414 10.45932425152005 0.10603041827791615 0.0026685833857283928 0.0025245441861131736

GOTERM_BP_DIRECT GO:0071222~cellular response to lipopolysaccharide 9 3.71900826446281 4.3586224240224977E-4 11009, 7099, 1437, 1234, 7124, 941, 942, 3593, 54106 183 190 19414 5.025194132873166 0.595300843056281 0.013703244742191943 0.012963599724782065

Annotation Cluster 6 Enrichment Score: 4.3618020145234535

Category Term Count % PValue Genes List Total Pop Hits Pop Total Fold Enrichment Bonferroni Benjamini FDR

GOTERM_BP_DIRECT GO:0043406~positive regulation of MAP kinase activity 10 4.132231404958678 4.2778148819659895E-8 7099, 2247, 7422, 7124, 2321, 3265, 5788, 2064, 958, 1956 183 77 19414 13.777588531686893 8.876072119479161E-5 1.4178581477972078E-5 1.341327973072732E-5

GOTERM_BP_DIRECT GO:0050679~positive regulation of epithelial cell proliferation 7 2.8925619834710745 5.847089195931868E-5 2247, 7422, 6489, 3265, 3397, 2064, 1956 183 72 19414 10.314055859137826 0.11425895734002778 0.0028215604840834013 0.002669264207352153

KEGG_PATHWAY hsa04510:Focal adhesion 8 3.3057851239669422 0.032842200679731134 7422, 3791, 2321, 3696, 3265, 207, 2064, 1956 128 203 8465 2.60621921182266 0.9997382765829108 0.08450024549889155 0.05405278861872416

Annotation Cluster 7 Enrichment Score: 3.8784008460163477

Category Term Count % PValue Genes List Total Pop Hits Pop Total Fold Enrichment Bonferroni Benjamini FDR

UP_KW_MOLECULAR_FUNCTION KW-0825~Tumor antigen 8 3.3057851239669422 3.2014499150722276E-7 7299, 4100, 4101, 4102, 9947, 4105, 51438, 4072 130 42 11749 17.214652014652014 2.0168934298347985E-5 1.0084567232477518E-5 9.604349745216683E-6

INTERPRO IPR002190:MAGE protein 6 2.479338842975207 2.8605937057946186E-5 4100, 4101, 4102, 9947, 4105, 51438 183 38 19144 16.517687661777398 0.01211235119800591 0.003046532296671269 0.0029535630012329436

UP_SEQ_FEATURE DOMAIN:MAGE 6 2.479338842975207 3.0101535540417204E-5 4100, 4101, 4102, 9947, 4105, 51438 188 40 20543 16.3906914893617 0.04152682367077942 0.006059009082349691 0.005938602940188022

SMART SM01373:SM01373 6 2.479338842975207 7.316717868966305E-5 4100, 4101, 4102, 9947, 4105, 51438 122 38 10378 13.431406384814494 0.007871202958754764 0.0041529004605676415 0.003999089332398469

INTERPRO IPR021072:Melanoma associated antigen, MAGE, N-terminal 4 1.6528925619834711 0.0014766952143088537 4100, 4101, 4102, 4105 183 24 19144 17.435336976320585 0.4671616014409343 0.05070621356140467 0.04915884084708951

SMART SM01392:SM01392 4 1.6528925619834711 0.002616826051561737 4100, 4101, 4102, 4105 122 24 10378 14.1775956284153 0.2464708985638232 0.09420573785622254 0.09071663645414021

GOTERM_MF_DIRECT GO:0042826~histone deacetylase binding 6 2.479338842975207 0.009107947228827438 7153, 4100, 7157, 4101, 4102, 4105 185 131 18945 4.690323911697958 0.9847229399693841 0.219070099135481 0.21283834576838853

Annotation Cluster 8 Enrichment Score: 3.876374095458672

Category Term Count % PValue Genes List Total Pop Hits Pop Total Fold Enrichment Bonferroni Benjamini FDR

KEGG_PATHWAY hsa05330:Allograft rejection 11 4.545454545454546 1.339297151965401E-10 3106, 3458, 3105, 7124, 941, 942, 3123, 3593, 3592, 958, 959 128 38 8465 19.143708881578945 3.308064921991871E-8 3.308063965354541E-8 2.1160895001053335E-8

KEGG_PATHWAY hsa04514:Cell adhesion molecules 16 6.6115702479338845 1.0527861125226417E-8 5133, 3123, 1493, 3383, 6382, 3106, 3105, 941, 1001, 942, 965, 3696, 5788, 925, 958, 959 128 157 8465 6.739649681528663 2.6003783338834907E-6 3.279201752580458E-7 2.09762703201503E-7

KEGG_PATHWAY hsa04940:Type I diabetes mellitus 10 4.132231404958678 1.0620896364633064E-8 3329, 3106, 3458, 3105, 7124, 941, 942, 3123, 3593, 3592 128 43 8465 15.379723837209301 2.623357979958385E-6 3.279201752580458E-7 2.09762703201503E-7

KEGG_PATHWAY hsa05416:Viral myocarditis 10 4.132231404958678 2.2464976144344438E-7 3106, 3105, 27, 941, 942, 3123, 3383, 958, 959, 1604 128 60 8465 11.022135416666666 5.548695785517754E-5 3.8051472579913293E-6 2.4340618087555873E-6

KEGG_PATHWAY hsa05320:Autoimmune thyroid disease 9 3.71900826446281 1.0353384323321398E-6 3106, 3105, 941, 942, 3123, 3440, 1493, 958, 959 128 53 8465 11.230100235849056 2.556960293627064E-4 1.2797970079054605E-5 8.186555759071367E-6

KEGG_PATHWAY hsa05332:Graft-versus-host disease 7 2.8925619834710745 3.414030062867469E-5 3106, 3458, 3105, 7124, 941, 942, 3123 128 42 8465 11.022135416666668 0.008397341897737332 2.465164739131549E-4 1.576906999120586E-4

BIOCARTA h_th1th2Pathway:Th1/Th2 Differentiation 7 2.8925619834710745 4.660373267955886E-5 3458, 942, 3123, 3593, 3592, 958, 959 60 20 1623 9.467500000000001 0.008307562428799597 0.0029159229354633505 0.002769312285076925

BBID 58.(CD40L)_immnosurveillance 7 2.8925619834710745 3.200729188365703E-4 3458, 7124, 3383, 3592, 925, 958, 959 28 16 388 6.0625 0.021533219770591416 0.02144488556205021 0.02144488556205021

BIOCARTA h_blymphocytePathway:B Lymphocyte Cell Surface Molecules 5 2.066115702479339 4.301260148309427E-4 941, 3123, 5788, 3383, 958 60 11 1623 12.295454545454545 0.0741185901511685 0.015398511330947747 0.014624284504252051

GOTERM_BP_DIRECT GO:0023035~CD40 signaling pathway 3 1.2396694214876034 0.00374340335266357 942, 958, 959 183 10 19414 31.82622950819672 0.9995828801219508 0.06638941843399067 0.06280598958357768

KEGG_PATHWAY hsa04672:Intestinal immune network for IgA production 5 2.066115702479339 0.00607942609476402 941, 942, 3123, 958, 959 128 49 8465 6.748246173469387 0.7782492594861605 0.019501535654632637 0.012474666532113184

BIOCARTA h_asbcellPathway:Antigen Dependent B Cell Activation 4 1.6528925619834711 0.010068770005155107 941, 3123, 958, 959 60 13 1623 8.323076923076924 0.8365821272713896 0.20025664788030711 0.190187877875152

GOTERM_BP_DIRECT GO:0042130~negative regulation of T cell proliferation 4 1.6528925619834711 0.010885339434556846 941, 942, 3123, 1493 183 49 19414 8.660198505631762 0.9999999998629826 0.151591136420842 0.1434088678525845

KEGG_PATHWAY hsa05310:Asthma 4 1.6528925619834711 0.010912293412043349 7124, 3123, 958, 959 128 31 8465 8.533266129032258 0.9334732058233738 0.0328699569850574 0.02102612633052255

KEGG_PATHWAY hsa05322:Systemic lupus erythematosus 7 2.8925619834710745 0.016880284623738882 3458, 7124, 941, 942, 3123, 958, 959 128 137 8465 3.3790488138686134 0.9850795419992959 0.047379889796176175 0.030307783756258446

BIOCARTA h_ctla4Pathway:The Co-Stimulatory Signal During T-cell Activation 4 1.6528925619834711 0.029219514703740714 941, 942, 3123, 1493 60 19 1623 5.6947368421052635 0.9950493101762212 0.32689332074809924 0.3104573437272451

BIOCARTA h_bbcellPathway:Bystander B Cell Activation 3 1.2396694214876034 0.039688300920162685 3123, 958, 959 60 9 1623 9.016666666666667 0.999289134674253 0.39467810359495115 0.37483395313486984

BBID 3.T_cell_receptor 3 1.2396694214876034 0.41322646296267157 941, 942, 1493 28 20 388 2.0785714285714287 0.9999999999999998 1.0 1.0

Annotation Cluster 9 Enrichment Score: 3.3621796666645927

Category Term Count % PValue Genes List Total Pop Hits Pop Total Fold Enrichment Bonferroni Benjamini FDR

BIOCARTA h_dcPathway:Dendritic cells in regulating TH1 and TH2 Development 10 4.132231404958678 2.053755785862532E-8 7099, 1437, 3458, 51284, 3456, 945, 3593, 54106, 3592, 958 60 22 1623 12.295454545454545 3.676216140391375E-6 3.6762228566939325E-6 3.4913848359663044E-6

KEGG_PATHWAY hsa04620:Toll-like receptor signaling pathway 12 4.958677685950414 3.8379481375823866E-7 7099, 51284, 3456, 7124, 941, 942, 207, 3440, 3593, 54106, 3592, 958 128 104 8465 7.630709134615385 9.479284405156108E-5 5.576312882252056E-6 3.567034151400101E-6

GOTERM_BP_DIRECT GO:0032729~positive regulation of interferon-gamma production 9 3.71900826446281 7.563783261777975E-7 3329, 7099, 51284, 3105, 7124, 3265, 3593, 54106, 3592 183 79 19414 12.08590993982154 0.0015682546219550897 1.1210607334420929E-4 1.060550467347869E-4

GOTERM_BP_DIRECT GO:0051607~defense response to virus 12 4.958677685950414 1.3534438099923094E-5 3458, 51284, 598, 9447, 3456, 4343, 3440, 5788, 3593, 54106, 958, 282617 183 234 19414 5.44038111251226 0.027693455371097464 8.2599879580413E-4 7.81414764416148E-4

KEGG_PATHWAY hsa05164:Influenza A 12 4.958677685950414 4.8554741932912205E-5 7099, 3458, 51284, 8743, 3456, 7124, 3123, 207, 3440, 3593, 3383, 3592 128 171 8465 4.640899122807018 0.011921679313151912 3.331394793730365E-4 2.131013673722258E-4

BIOCARTA h_inflamPathway:Cytokines and Inflammatory Response 8 3.3057851239669422 4.887021679547515E-5 1437, 3458, 3456, 7124, 3123, 3593, 3592, 7042 60 29 1623 7.462068965517242 0.00870983029938388 0.0029159229354633505 0.002769312285076925

KEGG_PATHWAY hsa05152:Tuberculosis 12 4.958677685950414 7.745486301733962E-5 3329, 7099, 3458, 3456, 7124, 3123, 207, 3440, 3593, 54106, 3592, 7042 128 180 8465 4.408854166666667 0.01895023525145767 4.897322657787991E-4 3.132700323605274E-4

KEGG_PATHWAY hsa05142:Chagas disease 9 3.71900826446281 1.3864802736551714E-4 7099, 3458, 3456, 7124, 207, 3593, 54106, 3592, 7042 128 102 8465 5.8352481617647065 0.03366859771248465 7.444796252017986E-4 4.7622583312503715E-4

KEGG_PATHWAY hsa05162:Measles 10 4.132231404958678 2.3018349040026883E-4 7099, 51284, 598, 3456, 7157, 207, 3440, 3593, 54106, 3592 128 139 8465 4.757756294964028 0.05527544173609533 0.0010933715794012768 6.994036823700475E-4

KEGG_PATHWAY hsa05171:Coronavirus disease - COVID-19 12 4.958677685950414 7.021469249967627E-4 7099, 1437, 51284, 4153, 3456, 7124, 3440, 3593, 3592, 6868, 8829, 1956 128 232 8465 3.420662715517241 0.15927545533954912 0.0029497430541014884 0.0018868801722592516

KEGG_PATHWAY hsa04936:Alcoholic liver disease 9 3.71900826446281 0.0012875082975789733 7099, 3456, 7124, 217, 207, 3440, 3593, 3592, 2308 128 142 8465 4.191516285211268 0.27255686546509905 0.004746485813462782 0.003036213597275788

BIOCARTA h_cytokinePathway:Cytokine Network 5 2.066115702479339 0.007059989116634557 3458, 3456, 7124, 3593, 3592 60 22 1623 6.1477272727272725 0.7186680945506729 0.17785436055258505 0.16891196253597462

KEGG_PATHWAY hsa04380:Osteoclast differentiation 7 2.8925619834710745 0.012411988699869215 1436, 3458, 3456, 7124, 207, 11027, 7042 128 128 8465 3.61663818359375 0.9542675066876982 0.036497157248424957 0.02334635969737305

KEGG_PATHWAY hsa04622:RIG-I-like receptor signaling pathway 5 2.066115702479339 0.021688151617444303 3456, 7124, 3440, 3593, 3592 128 71 8465 4.657240316901409 0.995554747961101 0.0576018650484811 0.03684653715651828

KEGG_PATHWAY hsa05168:Herpes simplex virus 1 infection 13 5.371900826446281 0.08217280387697062 7124, 7157, 3123, 3440, 3593, 3592, 3106, 3458, 3105, 598, 3456, 207, 54106 128 512 8465 1.6791534423828125 0.9999999993662065 0.1828529960145202 0.11696669380685908

KEGG_PATHWAY hsa04217:Necroptosis 6 2.479338842975207 0.0906210776938031 7099, 3458, 8743, 3456, 7124, 3440 128 159 8465 2.495577830188679 0.9999999999354366 0.19634566833657338 0.12559763399667448

KEGG_PATHWAY hsa04621:NOD-like receptor signaling pathway 6 2.479338842975207 0.14730360130830739 7099, 598, 9447, 3456, 7124, 3440 128 186 8465 2.1333165322580645 1.0 0.29580479287115385 0.18921926021717536

BIOCARTA h_il1rPathway:Signal transduction through IL1R 3 1.2396694214876034 0.3391639215754326 3456, 7124, 7042 60 33 1623 2.459090909090909 1.0 1.0 0.9550561797752809

Annotation Cluster 10 Enrichment Score: 3.3462573048853317

Category Term Count % PValue Genes List Total Pop Hits Pop Total Fold Enrichment Bonferroni Benjamini FDR

GOTERM_BP_DIRECT GO:0032735~positive regulation of interleukin-12 production 7 2.8925619834710745 3.282250657336291E-6 3329, 7099, 3458, 3593, 54106, 958, 959 183 44 19414 16.877545951316446 0.00678754116456437 3.0957591427149106E-4 2.9286627456141546E-4

GOTERM_BP_DIRECT GO:0032733~positive regulation of interleukin-10 production 6 2.479338842975207 4.40066257982646E-5 3329, 7099, 6375, 3593, 54106, 959 183 42 19414 15.155347384855585 0.08727053604976043 0.0023413781674717703 0.002215000165179318

GOTERM_BP_DIRECT GO:0032755~positive regulation of interleukin-6 production 7 2.8925619834710745 4.2120053251854737E-4 3329, 7099, 3458, 51284, 7124, 54106, 11027 183 103 19414 7.209825454931297 0.5827940188032034 0.01344601699963055 0.01272025608206013

GOTERM_BP_DIRECT GO:0051770~positive regulation of nitric-oxide synthase biosynthetic process 4 1.6528925619834711 5.961543922275036E-4 7099, 3458, 3791, 54106 183 18 19414 23.57498482088646 0.7098592816628139 0.017472022965884227 0.016528954738328064

GOTERM_BP_DIRECT GO:0032727~positive regulation of interferon-alpha production 4 1.6528925619834711 0.0017977190724351795 3329, 7099, 51284, 54106 183 26 19414 16.32114333753678 0.9760939588773558 0.03845636160106183 0.03638064473392018

GOTERM_BP_DIRECT GO:0032760~positive regulation of tumor necrosis factor production 6 2.479338842975207 0.0032378985160541668 7099, 3458, 5788, 3593, 54106, 11027 183 106 19414 6.004948963810702 0.9988049175422277 0.059987851971539254 0.056749953455485086

GOTERM_BP_DIRECT GO:0002755~MyD88-dependent toll-like receptor signaling pathway 3 1.2396694214876034 0.017851566543729745 3329, 7099, 54106 183 22 19414 14.466467958271238 0.9999999999999999 0.2141156102788394 0.20255852673607797

Annotation Cluster 11 Enrichment Score: 3.1341009893584673

Category Term Count % PValue Genes List Total Pop Hits Pop Total Fold Enrichment Bonferroni Benjamini FDR

UP_SEQ_FEATURE DOMAIN:Ig-like V-type 11 4.545454545454546 5.543600863440242E-7 57823, 1048, 5133, 941, 634, 942, 945, 1493, 7070, 1084, 925 188 138 20543 8.710029293863705 7.807886040492873E-4 1.5621867233174604E-4 1.531142558482195E-4

INTERPRO IPR013783:Immunoglobulin-like fold 29 11.983471074380166 2.33070253867971E-6 3563, 3123, 6490, 3383, 1084, 445347, 1436, 57823, 3598, 2321, 634, 11027, 1438, 4162, 5133, 3791, 1493, 3593, 7070, 3106, 1048, 3105, 941, 942, 965, 5788, 945, 925, 1969 183 1115 19144 2.7208507927172927 9.923876958217726E-4 4.964396407387782E-4 4.812900742373601E-4

GOTERM_BP_DIRECT GO:0018108~peptidyl-tyrosine phosphorylation 8 3.3057851239669422 4.130649074378629E-6 1436, 3791, 27, 2321, 5788, 2064, 1956, 7272 183 70 19414 12.124277907884466 0.008534487249491574 3.4284387317342615E-4 3.243385653202099E-4

UP_SEQ_FEATURE DOMAIN:Immunoglobulin V-set 6 2.479338842975207 1.540822756286754E-5 1048, 5133, 634, 1493, 1084, 925 188 35 20543 18.732218844984803 0.02147638630802451 0.003618365439346727 0.0035464603773866783

UP_SEQ_FEATURE DOMAIN:Ig-like 18 7.43801652892562 1.2178977800474615E-4 4162, 5133, 3123, 3593, 7070, 1436, 3106, 57823, 3105, 1048, 941, 942, 2321, 634, 965, 945, 11027, 925 188 662 20543 2.971122324355595 0.15769428419068587 0.017160179720868734 0.01681916834245544

UP_SEQ_FEATURE DOMAIN:Ig-like C2-type 3 8 3.3057851239669422 1.8170247707972002E-4 4162, 1436, 1048, 3791, 2321, 634, 11027, 3383 188 129 20543 6.776513277255484 0.22589058524253425 0.022979108123888686 0.022522461546550937

UP_KW_DOMAIN KW-0393~Immunoglobulin domain 20 8.264462809917356 2.3314332792405106E-4 4162, 5133, 3791, 1493, 3593, 7070, 3383, 1084, 1436, 57823, 3105, 1048, 941, 942, 2321, 634, 965, 945, 11027, 925 149 752 14504 2.5888904755104956 0.004420440655211855 0.0016029861249093735 0.0014342507433399657

INTERPRO IPR013151:Immunoglobulin 7 2.8925619834710745 5.283041519598909E-4 4162, 1436, 2321, 634, 945, 7070, 11027 183 106 19144 6.908341066089288 0.20157723339568923 0.025006396526101503 0.0242432905288261

UP_SEQ_FEATURE DOMAIN:Ig-like C2-type 4 6 2.479338842975207 8.199877457559237E-4 1436, 1048, 3791, 2321, 11027, 3383 188 80 20543 8.19534574468085 0.6852059566144277 0.053289131755115966 0.0522301568160505

GOTERM_BP_DIRECT GO:0002244~hematopoietic progenitor cell differentiation 6 2.479338842975207 9.334689759732713E-4 7153, 1436, 3791, 7157, 2321, 5788 183 80 19414 7.95655737704918 0.8559871473141014 0.023912939816599234 0.02262221728192014

INTERPRO IPR007110:Immunoglobulin-like domain 18 7.43801652892562 9.898804992666114E-4 4162, 5133, 3791, 3123, 3593, 7070, 1436, 3106, 57823, 3105, 1048, 941, 942, 2321, 634, 945, 11027, 925 183 762 19144 2.471150122628114 0.34419903391125717 0.042168909268757646 0.04088206461971105

INTERPRO IPR003599:Immunoglobulin subtype 14 5.785123966942149 0.0015535642220759005 4162, 5133, 3791, 1493, 3383, 1436, 1048, 941, 942, 2321, 634, 945, 11027, 925 183 524 19144 2.7949776832269637 0.4843530640417978 0.05070621356140467 0.04915884084708951

UP_SEQ_FEATURE DOMAIN:Ig-like C2-type 5 5 2.066115702479339 0.0018026785342104886 1436, 1048, 3791, 2321, 3383 188 57 20543 9.585199701381113 0.9213121239472305 0.09758578026140743 0.09564653125692239

INTERPRO IPR013106:Immunoglobulin V-set 13 5.371900826446281 0.002016366672174386 4162, 5133, 1493, 7070, 1084, 57823, 1048, 941, 942, 2321, 634, 945, 925 183 475 19144 2.863065861374748 0.5767699395653456 0.057264813489752556 0.05551729570720143

UP_SEQ_FEATURE DOMAIN:Ig-like C2-type 1 8 3.3057851239669422 0.00285736778925722 4162, 1436, 1048, 3791, 2321, 634, 11027, 3383 188 206 20543 4.243544722164842 0.9822575307637172 0.1220009459110128 0.11957651263527942

UP_SEQ_FEATURE DOMAIN:Ig-like C2-type 2 8 3.3057851239669422 0.00285736778925722 4162, 1436, 1048, 3791, 2321, 634, 11027, 3383 188 206 20543 4.243544722164842 0.9822575307637172 0.1220009459110128 0.11957651263527942

INTERPRO IPR001824:Tyrosine-protein kinase, receptor class III, conserved site 3 1.2396694214876034 0.0030969562268416367 1436, 3791, 2321 183 9 19144 34.87067395264117 0.7332252188130965 0.08245645953965858 0.07994018260534974

SMART SM00409:IG 14 5.785123966942149 0.007948755315792078 4162, 5133, 3791, 1493, 3383, 1436, 1048, 941, 942, 2321, 634, 945, 11027, 925 122 524 10378 2.2727443373795517 0.5776393161968163 0.1716931148211089 0.16533411056847525

INTERPRO IPR003598:Immunoglobulin subtype 2 8 3.3057851239669422 0.011542427093615762 4162, 1436, 1048, 3791, 2321, 634, 3593, 11027 183 257 19144 3.2564053496629883 0.9928858654835437 0.21304909108966855 0.20654759300477255

SMART SM00408:IGc2 8 3.3057851239669422 0.03059822803083818 4162, 1436, 1048, 3791, 2321, 634, 3593, 11027 122 257 10378 2.647955603750718 0.965133255660451 0.2833334178151797 0.27283958752572857

UP_SEQ_FEATURE DOMAIN:Ig-like C2-type 6 3 1.2396694214876034 0.04901962184196782 1048, 3791, 2321 188 39 20543 8.405482815057283 1.0 0.8003651833327734 0.7844601264602982

SMART SM00406:IGv 6 2.479338842975207 0.33605693201957626 5133, 2321, 942, 1493, 7070, 925 122 328 10378 1.556077568972411 1.0 1.0 0.9719626168224299

Annotation Cluster 12 Enrichment Score: 3.0141410437300484

Category Term Count % PValue Genes List Total Pop Hits Pop Total Fold Enrichment Bonferroni Benjamini FDR

GOTERM_CC_DIRECT GO:0043235~receptor complex 16 6.6115702479338845 1.6654455635464477E-9 7099, 3791, 3563, 2022, 3593, 2064, 780, 1436, 51284, 3598, 2321, 1969, 925, 8829, 1956, 1438 188 220 20624 7.978336557059961 5.529277732341242E-7 6.911599088717758E-8 6.391147350109493E-8

GOTERM_BP_DIRECT GO:0018108~peptidyl-tyrosine phosphorylation 8 3.3057851239669422 4.130649074378629E-6 1436, 3791, 27, 2321, 5788, 2064, 1956, 7272 183 70 19414 12.124277907884466 0.008534487249491574 3.4284387317342615E-4 3.243385653202099E-4

GOTERM_BP_DIRECT GO:0033674~positive regulation of kinase activity 8 3.3057851239669422 4.5464803537014E-6 1436, 3791, 2321, 3123, 2064, 1969, 1956, 780 183 71 19414 11.953513430308627 0.009389607909309206 3.628441051511694E-4 3.4325926670445567E-4

GOTERM_MF_DIRECT GO:0004714~transmembrane receptor protein tyrosine kinase activity 7 2.8925619834710745 1.2157059720983368E-5 1436, 3791, 2321, 2064, 1969, 1956, 780 185 53 18945 13.52524222335543 0.005540405093232836 6.944720365611749E-4 6.747168145145769E-4

INTERPRO IPR020635:Tyrosine-protein kinase, catalytic domain 8 3.3057851239669422 2.2345151945609286E-5 1436, 3791, 27, 2321, 2064, 1969, 1956, 780 183 89 19144 9.403327807453797 0.009473977478943518 0.003046532296671269 0.0029535630012329436

GOTERM_MF_DIRECT GO:0019838~growth factor binding 6 2.479338842975207 2.7724468002949136E-5 1436, 3791, 2321, 2064, 1969, 8829 185 37 18945 16.60628195763331 0.01259032773655211 0.0012670081877347757 0.0012309663793309417

UP_KW_MOLECULAR_FUNCTION KW-0829~Tyrosine-protein kinase 9 3.71900826446281 3.528675941974436E-5 1436, 3791, 27, 2321, 2064, 1969, 1956, 7272, 780 130 114 11749 7.1350202429149805 0.002220635798847237 5.557664608609737E-4 5.293013912961654E-4

INTERPRO IPR008266:Tyrosine-protein kinase, active site 8 3.3057851239669422 3.655611159436123E-5 1436, 3791, 27, 2321, 2064, 1969, 1956, 780 183 96 19144 8.717668488160292 0.015452553129365887 0.0031145807078395765 0.0030195348176942373

GOTERM_BP_DIRECT GO:0046777~protein autophosphorylation 10 4.132231404958678 4.952751771867686E-5 1436, 3791, 2321, 207, 5788, 2064, 7070, 1956, 7272, 780 183 179 19414 5.926672161675367 0.09766744833599739 0.0025065755918598654 0.0023712809093112846

GOTERM_BP_DIRECT GO:0007275~multicellular organism development 9 3.71900826446281 6.505271684796777E-5 1436, 5133, 3791, 2321, 1001, 2064, 1969, 1956, 780 183 144 19414 6.630464480874316 0.1262742835922125 0.0030113695799874178 0.002848828185790506

SMART SM00219:TyrKc 8 3.3057851239669422 7.690556408458595E-5 1436, 3791, 27, 2321, 2064, 1969, 1956, 780 122 89 10378 7.6463437097071285 0.008271719812093403 0.0041529004605676415 0.003999089332398469

INTERPRO IPR001245:Serine-threonine/tyrosine-protein kinase catalytic domain 9 3.71900826446281 8.697732148920416E-5 1436, 2984, 3791, 27, 2321, 2064, 1969, 1956, 780 183 148 19144 6.36154186973859 0.03637585396576615 0.006175389825733495 0.005986938962506886

GOTERM_MF_DIRECT GO:0004713~protein tyrosine kinase activity 8 3.3057851239669422 1.1808736228766572E-4 1436, 3791, 27, 2321, 2064, 1969, 1956, 7272 185 113 18945 7.249940205692418 0.05253862797048259 0.00449716038045527 0.004369232404643632

GOTERM_BP_DIRECT GO:0007169~transmembrane receptor protein tyrosine kinase signaling pathway 8 3.3057851239669422 2.863107644878068E-4 1436, 3791, 2321, 2064, 1969, 925, 1956, 780 183 135 19414 6.2866626189030566 0.4479849123738512 0.009739259611675394 0.009213574273599421

UP_SEQ_FEATURE DOMAIN:Serine-threonine/tyrosine-protein kinase catalytic 4 1.6528925619834711 3.809089186113014E-4 1436, 3791, 2064, 1956 188 16 20543 27.31781914893617 0.4153856671965651 0.03354379164520773 0.03287720103763795

GOTERM_BP_DIRECT GO:0030335~positive regulation of cell migration 10 4.132231404958678 8.40697665975244E-4 1437, 4162, 1436, 7422, 3791, 2321, 3265, 1969, 6868, 1956 183 262 19414 4.0491386142743915 0.825386679099699 0.022364713549982453 0.02115755792704364

UP_SEQ_FEATURE DOMAIN:Ig-like C2-type 5 5 2.066115702479339 0.0018026785342104886 1436, 1048, 3791, 2321, 3383 188 57 20543 9.585199701381113 0.9213121239472305 0.09758578026140743 0.09564653125692239

INTERPRO IPR001824:Tyrosine-protein kinase, receptor class III, conserved site 3 1.2396694214876034 0.0030969562268416367 1436, 3791, 2321 183 9 19144 34.87067395264117 0.7332252188130965 0.08245645953965858 0.07994018260534974

UP_SEQ_FEATURE DOMAIN:Protein kinase 12 4.958677685950414 0.006905320599949703 1436, 983, 2984, 3791, 27, 2321, 207, 2064, 1969, 1956, 7272, 780 188 507 20543 2.58630240463301 0.9999424781205271 0.24323991813322832 0.2384061937132635

INTERPRO IPR000719:Protein kinase, catalytic domain 12 4.958677685950414 0.008602424442102869 1436, 983, 2984, 3791, 27, 2321, 207, 2064, 1969, 1956, 7272, 780 183 501 19144 2.505677170249665 0.9747892845707761 0.18323164061679112 0.17764006472942423

GOTERM_MF_DIRECT GO:0004712~protein serine/threonine/tyrosine kinase activity 11 4.545454545454546 0.011027873134178554 1436, 983, 3791, 27, 2321, 207, 2064, 1969, 1956, 7272, 780 185 440 18945 2.5601351351351354 0.993703320347249 0.23715770573924355 0.23041142526963707

INTERPRO IPR017441:Protein kinase, ATP binding site 10 4.132231404958678 0.012502880932492286 1436, 983, 3791, 27, 2321, 207, 2064, 1969, 1956, 7272 183 390 19144 2.682359534818551 0.9952981725859418 0.21304909108966855 0.20654759300477255

INTERPRO IPR011009:Protein kinase-like domain 12 4.958677685950414 0.015544576064325728 1436, 983, 2984, 3791, 27, 2321, 207, 2064, 1969, 1956, 7272, 780 183 546 19144 2.299165315558758 0.9987366610144657 0.24525886679269482 0.2377744412802417

GOTERM_BP_DIRECT GO:0038083~peptidyl-tyrosine autophosphorylation 3 1.2396694214876034 0.017851566543729745 3791, 1956, 780 183 22 19414 14.466467958271238 0.9999999999999999 0.2141156102788394 0.20255852673607797

GOTERM_MF_DIRECT GO:0016301~kinase activity 7 2.8925619834710745 0.024596167934994376 983, 2323, 8852, 207, 2064, 1969, 1956 185 229 18945 3.1302962350997285 0.9999885896999826 0.41631291652934926 0.40447031715324083

UP_SEQ_FEATURE ACT_SITE:Proton acceptor 14 5.785123966942149 0.05156904483955757 3791, 27, 2064, 7272, 780, 1436, 983, 2321, 216, 217, 207, 1969, 1956, 4758 188 861 20543 1.7767687251340596 1.0 0.8003651833327734 0.7844601264602982

UP_KW_MOLECULAR_FUNCTION KW-0418~Kinase 13 5.371900826446281 0.11780236002105993 3791, 27, 8852, 2064, 7272, 780, 1436, 983, 2323, 2321, 207, 1969, 1956 130 746 11749 1.5749329758713135 0.9996279072800437 0.7421548681326775 0.7068141601263596

GOTERM_MF_DIRECT GO:0005524~ATP binding 19 7.851239669421488 0.24166570113649796 7153, 9585, 3791, 27, 4343, 2064, 7272, 780, 7184, 3329, 1436, 2984, 983, 2321, 207, 1969, 1956, 3845, 3308 185 1543 18945 1.260986845562348 1.0 1.0 0.9736842105263158

UP_KW_LIGAND KW-0547~Nucleotide-binding 23 9.50413223140496 0.2450398786511088 7153, 9585, 3791, 27, 4343, 4893, 3265, 2064, 7272, 780, 7184, 3329, 51062, 1436, 2984, 983, 2321, 216, 207, 1969, 1956, 3845, 3308 71 1860 6858 1.1944116310767834 0.9994942355264869 1.0 1.0

UP_KW_LIGAND KW-0067~ATP-binding 18 7.43801652892562 0.2918026054408275 7153, 9585, 3791, 27, 4343, 2064, 7272, 780, 7184, 3329, 1436, 983, 2321, 207, 1969, 1956, 3845, 3308 71 1441 6858 1.2065564797529102 0.9999100156951477 1.0 1.0

GOTERM_MF_DIRECT GO:0004672~protein kinase activity 6 2.479338842975207 0.3107084831506337 983, 2984, 27, 207, 2064, 1956 185 378 18945 1.6254826254826258 1.0 1.0 0.9736842105263158

UP_KW_MOLECULAR_FUNCTION KW-0808~Transferase 24 9.917355371900827 0.4887852383999687 7015, 3791, 27, 6489, 4255, 8852, 2583, 2064, 4193, 7272, 780, 1436, 2875, 983, 2323, 952, 54894, 2321, 207, 9978, 1969, 1956, 3845, 5903 130 2048 11749 1.0591045673076924 1.0 1.0 0.967741935483871

Annotation Cluster 13 Enrichment Score: 2.959903838524912

Category Term Count % PValue Genes List Total Pop Hits Pop Total Fold Enrichment Bonferroni Benjamini FDR

GOTERM_BP_DIRECT GO:0042102~positive regulation of T cell proliferation 6 2.479338842975207 4.1357446561237195E-4 941, 942, 5788, 3593, 3592, 959 183 67 19414 9.500367017372156 0.5761368024380453 0.013408859627276122 0.012685104312454471

GOTERM_BP_DIRECT GO:0031295~T cell costimulation 5 2.066115702479339 5.246530282193334E-4 6366, 941, 942, 207, 959 183 40 19414 13.260928961748633 0.6634271690323184 0.015777609181958214 0.014925998469486251

KEGG_PATHWAY hsa04672:Intestinal immune network for IgA production 5 2.066115702479339 0.00607942609476402 941, 942, 3123, 958, 959 128 49 8465 6.748246173469387 0.7782492594861605 0.019501535654632637 0.012474666532113184

Annotation Cluster 14 Enrichment Score: 2.955120209714945

Category Term Count % PValue Genes List Total Pop Hits Pop Total Fold Enrichment Bonferroni Benjamini FDR

KEGG_PATHWAY hsa05330:Allograft rejection 11 4.545454545454546 1.339297151965401E-10 3106, 3458, 3105, 7124, 941, 942, 3123, 3593, 3592, 958, 959 128 38 8465 19.143708881578945 3.308064921991871E-8 3.308063965354541E-8 2.1160895001053335E-8

KEGG_PATHWAY hsa05144:Malaria 11 4.545454545454546 2.5000813833619547E-9 7099, 3458, 7124, 3043, 54106, 3383, 3592, 958, 959, 7042, 6382 128 50 8465 14.54921875 6.175199070979076E-7 1.106926075441929E-7 7.080741697158898E-8

KEGG_PATHWAY hsa04940:Type I diabetes mellitus 10 4.132231404958678 1.0620896364633064E-8 3329, 3106, 3458, 3105, 7124, 941, 942, 3123, 3593, 3592 128 43 8465 15.379723837209301 2.623357979958385E-6 3.279201752580458E-7 2.09762703201503E-7

KEGG_PATHWAY hsa05145:Toxoplasmosis 12 4.958677685950414 8.15947134469626E-7 7099, 3458, 598, 1234, 7124, 3123, 207, 3593, 3592, 958, 959, 7042 128 112 8465 7.085658482142858 2.0151871682527744E-4 1.1196607900777645E-5 7.16220262478894E-6

KEGG_PATHWAY hsa05143:African trypanosomiasis 7 2.8925619834710745 1.6087156762276246E-5 3458, 7124, 3043, 3593, 54106, 3383, 3592 128 37 8465 12.511613175675677 0.003965675539891467 1.3245092400940775E-4 8.472569228132156E-5

BIOCARTA h_th1th2Pathway:Th1/Th2 Differentiation 7 2.8925619834710745 4.660373267955886E-5 3458, 942, 3123, 3593, 3592, 958, 959 60 20 1623 9.467500000000001 0.008307562428799597 0.0029159229354633505 0.002769312285076925

KEGG_PATHWAY hsa05164:Influenza A 12 4.958677685950414 4.8554741932912205E-5 7099, 3458, 51284, 8743, 3456, 7124, 3123, 207, 3440, 3593, 3383, 3592 128 171 8465 4.640899122807018 0.011921679313151912 3.331394793730365E-4 2.131013673722258E-4

BIOCARTA h_inflamPathway:Cytokines and Inflammatory Response 8 3.3057851239669422 4.887021679547515E-5 1437, 3458, 3456, 7124, 3123, 3593, 3592, 7042 60 29 1623 7.462068965517242 0.00870983029938388 0.0029159229354633505 0.002769312285076925

GOTERM_BP_DIRECT GO:0042088~T-helper 1 type immune response 4 1.6528925619834711 6.530681016840182E-5 7099, 3123, 3265, 3593 183 9 19414 47.14996964177292 0.12673485851101174 0.0030113695799874178 0.002848828185790506

KEGG_PATHWAY hsa05152:Tuberculosis 12 4.958677685950414 7.745486301733962E-5 3329, 7099, 3458, 3456, 7124, 3123, 207, 3440, 3593, 54106, 3592, 7042 128 180 8465 4.408854166666667 0.01895023525145767 4.897322657787991E-4 3.132700323605274E-4

KEGG_PATHWAY hsa05142:Chagas disease 9 3.71900826446281 1.3864802736551714E-4 7099, 3458, 3456, 7124, 207, 3593, 54106, 3592, 7042 128 102 8465 5.8352481617647065 0.03366859771248465 7.444796252017986E-4 4.7622583312503715E-4

KEGG_PATHWAY hsa05146:Amoebiasis 9 3.71900826446281 1.3864802736551714E-4 5272, 7099, 1437, 3458, 4583, 7124, 3593, 3592, 7042 128 102 8465 5.8352481617647065 0.03366859771248465 7.444796252017986E-4 4.7622583312503715E-4

BBID 58.(CD40L)_immnosurveillance 7 2.8925619834710745 3.200729188365703E-4 3458, 7124, 3383, 3592, 925, 958, 959 28 16 388 6.0625 0.021533219770591416 0.02144488556205021 0.02144488556205021

BIOCARTA h_nktPathway:Selective expression of chemokine receptors during T-cell polarization 7 2.8925619834710745 3.607867755362383E-4 1437, 3458, 1234, 3593, 3592, 959, 7042 60 28 1623 6.762500000000001 0.06255059029883947 0.015398511330947747 0.014624284504252051

KEGG_PATHWAY hsa05321:Inflammatory bowel disease 7 2.8925619834710745 4.0570541894343985E-4 7099, 3458, 7124, 3123, 3593, 3592, 7042 128 65 8465 7.1219951923076925 0.0953702831810862 0.0018557266385005491 0.00118706400357525

GOTERM_BP_DIRECT GO:0009615~response to virus 7 2.8925619834710745 5.978378942543518E-4 6375, 3458, 598, 3456, 7124, 3696, 3592 183 110 19414 6.7510183805265775 0.7108716556812311 0.017472022965884227 0.016528954738328064

KEGG_PATHWAY hsa05140:Leishmaniasis 7 2.8925619834710745 0.00100603482620884 7099, 3458, 7124, 3123, 3593, 3592, 7042 128 77 8465 6.012073863636363 0.22012036169406723 0.003944295271009261 0.0025230714689047098

GOTERM_BP_DIRECT GO:0032700~negative regulation of interleukin-17 production 4 1.6528925619834711 0.0012500493105538813 7099, 3458, 3593, 3592 183 23 19414 18.449988120693753 0.9253895643428468 0.03016107348138725 0.028533102286247315

GOTERM_BP_DIRECT GO:0097191~extrinsic apoptotic signaling pathway 5 2.066115702479339 0.0014241930124787355 3458, 7124, 5788, 3592, 7042 183 52 19414 10.200714585960487 0.9480413406462405 0.03283556112103751 0.031063232038841752

GOTERM_BP_DIRECT GO:0098586~cellular response to virus 6 2.479338842975207 0.001583030458289585 3458, 51284, 3456, 3440, 3592, 282617 183 90 19414 7.072495446265939 0.9626488249718843 0.035704219575553135 0.033777052061113644

GOTERM_BP_DIRECT GO:0034393~positive regulation of smooth muscle cell apoptotic process 3 1.2396694214876034 0.0017795755239252582 3458, 3593, 3592 183 7 19414 45.46604215456674 0.9751751165215016 0.03845636160106183 0.03638064473392018

GOTERM_BP_DIRECT GO:0071346~cellular response to interferon-gamma 6 2.479338842975207 0.002408123565880719 7099, 6375, 6366, 7124, 965, 3593 183 99 19414 6.429541314787216 0.9932814478365535 0.048046696146177806 0.045453332305998566

BBID 97.Immune_injury_MS-lesions_MS_antigen 6 2.479338842975207 0.002873337117030061 3458, 7124, 941, 942, 3592, 925 28 16 388 5.196428571428571 0.17771495098329482 0.09625679342050705 0.09625679342050705

BIOCARTA h_cytokinePathway:Cytokine Network 5 2.066115702479339 0.007059989116634557 3458, 3456, 7124, 3593, 3592 60 22 1623 6.1477272727272725 0.7186680945506729 0.17785436055258505 0.16891196253597462

GOTERM_BP_DIRECT GO:0046427~positive regulation of JAK-STAT cascade 4 1.6528925619834711 0.008615193722935857 7124, 1442, 3593, 3592 183 45 19414 9.429993928354584 0.9999999840527112 0.12860810773447412 0.12166636890736035

KEGG_PATHWAY hsa05134:Legionellosis 5 2.066115702479339 0.010341756352322002 3329, 7099, 7124, 3593, 3592 128 57 8465 5.801123903508772 0.9232893064931142 0.03193017273779418 0.020424968795835954

BIOCARTA h_il18Pathway:IL 18 Signaling Pathway 3 1.2396694214876034 0.017739991586623687 3458, 3593, 3592 60 6 1623 13.525 0.9593989928782907 0.28867804490960364 0.2741635063387297

BIOCARTA h_no2il12Pathway:NO2-dependent IL 12 Pathway in NK cells 4 1.6528925619834711 0.021587923181728464 3458, 1234, 3593, 3592 60 17 1623 6.364705882352941 0.9798904182504147 0.3220198541274496 0.30582891174115323

GOTERM_BP_DIRECT GO:0045672~positive regulation of osteoclast differentiation 3 1.2396694214876034 0.02276324313633832 3458, 7124, 3593 183 25 19414 12.730491803278689 1.0 0.24859857635737903 0.2351802435612217

BBID 19.Cytokine_microglia 4 1.6528925619834711 0.034125211188869325 3458, 7124, 3592, 958 28 11 388 5.038961038961038 0.9056772487398158 0.6570232038443005 0.6570232038443005

BIOCARTA h_il12Pathway:IL12 and Stat4 Dependent Signaling Pathway in Th1 Development 4 1.6528925619834711 0.03809935400068382 3458, 1234, 3593, 3592 60 21 1623 5.152380952380952 0.9990443282775352 0.39467810359495115 0.37483395313486984

GOTERM_BP_DIRECT GO:0048662~negative regulation of smooth muscle cell proliferation 3 1.2396694214876034 0.03816222943126069 3458, 3593, 3592 183 33 19414 9.644311972180825 1.0 0.34132166409424974 0.32289851885157217

BBID 111.Stress_influences_immunity 3 1.2396694214876034 0.0588378988517284 3458, 7124, 3592 28 6 388 6.928571428571428 0.9838124440552228 0.6570232038443005 0.6570232038443005

BBID 112.StressandCRHinfluence 3 1.2396694214876034 0.0588378988517284 3458, 7124, 3592 28 6 388 6.928571428571428 0.9838124440552228 0.6570232038443005 0.6570232038443005

BBID 59.L-type_Ca2+_Tat_immune_cells 3 1.2396694214876034 0.10071046620652088 3458, 3592, 925 28 8 388 5.196428571428571 0.9992668908307986 0.7892548667953373 0.7892548667953373

BIOCARTA h_granulocytesPathway:Adhesion and Diapedesis of Granulocytes 3 1.2396694214876034 0.10076270330401144 3458, 7124, 3383 60 15 1623 5.41 0.9999999944601025 0.7841966909312195 0.7447678070296497

KEGG_PATHWAY hsa05133:Pertussis 4 1.6528925619834711 0.10563409552147941 7099, 7124, 3593, 3592 128 76 8465 3.480674342105263 0.9999999999989425 0.21982188590476145 0.14061480960709435

BBID 15.T-cell_polarization-chemokine_receptors 4 1.6528925619834711 0.1060193104650453 3458, 1234, 3592, 958 28 17 388 3.2605042016806722 0.9995098676394658 0.7892548667953373 0.7892548667953373

BBID 56.Macrophage_regulation_of_CD4+T_cells 3 1.2396694214876034 0.14852827890600115 3458, 7124, 3592 28 10 388 4.1571428571428575 0.9999821525751901 0.9046722442456433 0.9046722442456433

KEGG_PATHWAY hsa04658:Th1 and Th2 cell differentiation 4 1.6528925619834711 0.15978467468229388 3458, 3123, 3593, 3592 128 92 8465 2.8753396739130435 1.0 0.31322868767084594 0.20036490952224154

BBID 22.Cytokine-chemokine_CNS 3 1.2396694214876034 0.20006192131643263 3458, 3456, 3592 28 12 388 3.464285714285714 0.9999997442396605 0.957439194871499 0.957439194871499

BBID 18.Cytokine_astocytes 3 1.2396694214876034 0.3075654740124265 3458, 7124, 3383 28 16 388 2.5982142857142856 0.9999999999860121 0.9812803218491704 0.9812803218491704

Annotation Cluster 15 Enrichment Score: 2.785761971427013

Category Term Count % PValue Genes List Total Pop Hits Pop Total Fold Enrichment Bonferroni Benjamini FDR

GOTERM_MF_DIRECT GO:0019955~cytokine binding 6 2.479338842975207 8.123524773641296E-5 1436, 3598, 3563, 3593, 8829, 1438 185 46 18945 13.357226792009403 0.0364452958105238 0.003374955292321884 0.0032789499995424867

GOTERM_BP_DIRECT GO:0019221~cytokine-mediated signaling pathway 9 3.71900826446281 1.0417141156638182E-4 1436, 3456, 3598, 3563, 207, 3440, 3593, 11027, 1438 183 154 19414 6.199914839259102 0.19439919506461723 0.004323113580004845 0.00408976961809615

UP_SEQ_FEATURE DOMAIN:Type I cytokine receptor cytokine-binding 3 1.2396694214876034 8.094820049266929E-4 3598, 3563, 1438 188 5 20543 65.5627659574468 0.6805076744596598 0.053289131755115966 0.0522301568160505

INTERPRO IPR003532:Short hematopoietin receptor, family 2, conserved site 3 1.2396694214876034 0.0013148806421954453 3598, 3563, 1438 183 6 19144 52.306010928961754 0.42908088523852805 0.05070621356140467 0.04915884084708951

INTERPRO IPR015321:Interleukin-6 receptor alpha chain, binding 3 1.2396694214876034 0.006544445384297538 3598, 3563, 1438 183 13 19144 24.14123581336696 0.9390131170971681 0.14673335440582902 0.14225557598499383

GOTERM_MF_DIRECT GO:0004896~cytokine receptor activity 4 1.6528925619834711 0.018818325562306632 3598, 3563, 3593, 1438 185 58 18945 7.062441752096925 0.99983037544436 0.36809119746464325 0.35762033189125075

UP_SEQ_FEATURE MOTIF:WSXWS motif 3 1.2396694214876034 0.02848562717938687 3598, 3563, 1438 188 29 20543 11.303925165077036 1.0 0.557447898552168 0.5463701546490732

Annotation Cluster 16 Enrichment Score: 2.7222378642667167

Category Term Count % PValue Genes List Total Pop Hits Pop Total Fold Enrichment Bonferroni Benjamini FDR

UP_KW_BIOLOGICAL_PROCESS KW-0470~Melanin biosynthesis 4 1.6528925619834711 7.007885193227382E-5 7299, 7306, 6490, 1638 125 8 11262 45.048 0.004823937481718166 0.002417720391663447 0.002207483835866625

UP_SEQ_FEATURE TOPO_DOM:Lumenal, melanosome 3 1.2396694214876034 2.457840754738836E-4 7299, 7306, 1638 188 3 20543 109.27127659574467 0.29273674546584993 0.023087317489513464 0.02262852054862888

UP_SEQ_FEATURE DOMAIN:Tyrosinase copper-binding 3 1.2396694214876034 2.457840754738836E-4 7299, 7306, 1638 188 3 20543 109.27127659574467 0.29273674546584993 0.023087317489513464 0.02262852054862888

INTERPRO IPR002227:Tyrosinase 3 1.2396694214876034 2.679769242223938E-4 7299, 7306, 1638 183 3 19144 104.61202185792351 0.10789680919089928 0.014269771214842469 0.013834308712981079

INTERPRO IPR008922:Uncharacterised domain, di-copper centre 3 1.2396694214876034 2.679769242223938E-4 7299, 7306, 1638 183 3 19144 104.61202185792351 0.10789680919089928 0.014269771214842469 0.013834308712981079

GOTERM_BP_DIRECT GO:0042438~melanin biosynthetic process 4 1.6528925619834711 2.73376794604065E-4 7299, 7306, 6490, 1638 183 14 19414 30.310694769711162 0.4329650859640032 0.009614522861075166 0.009095570301826773

GOTERM_CC_DIRECT GO:0033162~melanosome membrane 4 1.6528925619834711 8.650019964176963E-4 7299, 7306, 6490, 1638 188 21 20624 20.895643363728468 0.24971709762330851 0.019145377520711677 0.017703707526682183

GOTERM_CC_DIRECT GO:0042470~melanosome 6 2.479338842975207 0.00258921400722879 7299, 2315, 7306, 6490, 1638, 7184 188 104 20624 6.328968903436988 0.5771483387026549 0.039073593199998105 0.036131304555419935

KEGG_PATHWAY hsa04916:Melanogenesis 6 2.479338842975207 0.017710851045270794 7299, 4893, 3265, 7306, 1638, 3845 128 101 8465 3.9286819306930694 0.9878907122499802 0.04860644675757651 0.03109238294614206

UP_KW_DISEASE KW-0015~Albinism 3 1.2396694214876034 0.036397399317239704 7299, 7306, 1638 68 21 4623 9.71218487394958 0.8114578933276153 0.5950376398761678 0.5950376398761678

KEGG_PATHWAY hsa00350:Tyrosine metabolism 3 1.2396694214876034 0.10117339753774716 7299, 7306, 1638 128 36 8465 5.511067708333334 0.9999999999963863 0.21542956199847887 0.13780514492210388

GOTERM_MF_DIRECT GO:0016491~oxidoreductase activity 5 2.066115702479339 0.17739180341692434 7299, 26872, 216, 7306, 1638 185 226 18945 2.2656063142788807 1.0 1.0 0.9736842105263158

Annotation Cluster 17 Enrichment Score: 2.635113780035

Category Term Count % PValue Genes List Total Pop Hits Pop Total Fold Enrichment Bonferroni Benjamini FDR

GOTERM_BP_DIRECT GO:0008284~positive regulation of cell proliferation 29 11.983471074380166 1.8867478037870962E-13 9585, 7422, 3265, 2250, 7042, 4193, 4072, 11009, 1437, 1436, 332, 3458, 2247, 598, 2323, 2321, 6868, 1956, 3791, 6489, 6862, 2064, 23532, 7272, 367, 942, 80312, 207, 3845 183 541 19414 5.686756966960597 3.9140080065891425E-10 3.9150016928582247E-10 3.7036859388340697E-10

GOTERM_BP_DIRECT GO:0001934~positive regulation of protein phosphorylation 17 7.024793388429752 1.7726836596553637E-10 3791, 7422, 7124, 3123, 3265, 2022, 2064, 2250, 1436, 332, 3458, 2247, 207, 958, 6868, 1956, 3845 183 213 19414 8.46707201313528 3.678316780053592E-7 9.1957964844622E-8 8.699445059758698E-8

KEGG_PATHWAY hsa05200:Pathways in cancer 30 12.396694214876034 6.937608502068346E-10 7422, 4893, 3563, 3265, 3440, 2250, 7042, 7184, 4193, 1436, 332, 354, 3458, 2247, 598, 2323, 9978, 2308, 1956, 1438, 7015, 7157, 3593, 2064, 3592, 3728, 367, 207, 7428, 3845 128 531 8465 3.73631709039548 1.7135890950648047E-7 5.711964333369605E-8 3.653807144422662E-8

KEGG_PATHWAY hsa04151:PI3K-Akt signaling pathway 24 9.917355371900827 2.031852015072808E-9 7099, 3791, 7422, 7157, 4893, 3563, 3265, 3440, 2064, 2250, 4193, 7184, 1436, 2247, 598, 3456, 2323, 1442, 2321, 3696, 207, 1969, 1956, 3845 128 354 8465 4.483580508474577 5.018673234769011E-7 1.106926075441929E-7 7.080741697158898E-8

KEGG_PATHWAY hsa05417:Lipid and atherosclerosis 19 7.851239669421488 2.6888892520856577E-9 7099, 8743, 7124, 7157, 4893, 3265, 3440, 3593, 3592, 3383, 7184, 3329, 598, 3456, 207, 958, 959, 3845, 3308 128 215 8465 5.844295058139535 6.641554182174048E-7 1.106926075441929E-7 7.080741697158898E-8

KEGG_PATHWAY hsa05205:Proteoglycans in cancer 17 7.024793388429752 5.969567783472805E-8 7099, 3791, 7422, 7124, 7157, 4893, 3265, 3593, 2064, 7042, 6382, 4193, 2247, 207, 2719, 1956, 3845 128 205 8465 5.484184451219512 1.4744724163406886E-5 1.4744832425177828E-6 9.431917097887032E-7

KEGG_PATHWAY hsa05166:Human T-cell leukemia virus 1 infection 17 7.024793388429752 1.8072372146755196E-7 9232, 7015, 7124, 7157, 4893, 3123, 3265, 3383, 7042, 1437, 3106, 3105, 598, 207, 958, 8829, 3845 128 222 8465 5.06422438063063 4.463776693264254E-5 3.5535757633074188E-6 2.2731375328039356E-6

KEGG_PATHWAY hsa05215:Prostate cancer 12 4.958677685950414 1.870303033319694E-7 354, 367, 7157, 4893, 3265, 207, 2064, 2308, 1956, 3845, 7184, 4193 128 97 8465 8.181378865979381 4.619542220751782E-5 3.5535757633074188E-6 2.2731375328039356E-6

KEGG_PATHWAY hsa04660:T cell receptor signaling pathway 12 4.958677685950414 3.8379481375823866E-7 1437, 3458, 5133, 7124, 4893, 3265, 207, 1493, 5788, 925, 959, 3845 128 104 8465 7.630709134615385 9.479284405156108E-5 5.576312882252056E-6 3.567034151400101E-6

KEGG_PATHWAY hsa05167:Kaposi sarcoma-associated herpesvirus infection 15 6.198347107438017 1.1028210885613687E-6 7422, 7157, 4893, 3265, 3440, 3383, 1437, 3106, 3105, 2247, 3456, 1234, 942, 207, 3845 128 194 8465 5.113361791237113 2.7235986239393384E-4 1.2971276613078956E-5 8.297415809176011E-6

KEGG_PATHWAY hsa05219:Bladder cancer 8 3.3057851239669422 2.1326980196451495E-6 7422, 7157, 4893, 3265, 2064, 1956, 3845, 4193 128 41 8465 12.903963414634147 5.266382499472533E-4 2.287168083858159E-5 1.4630467904841665E-5

KEGG_PATHWAY hsa05226:Gastric cancer 13 5.371900826446281 2.1465332952037977E-6 7015, 4583, 7157, 4893, 3265, 2064, 2250, 7042, 3728, 2247, 207, 1956, 3845 128 149 8465 5.769976929530201 5.300537648025294E-4 2.287168083858159E-5 1.4630467904841665E-5

KEGG_PATHWAY hsa04014:Ras signaling pathway 16 6.6115702479338845 2.2223495551658225E-6 3791, 7422, 27, 4893, 3265, 2250, 1436, 2247, 598, 2323, 2321, 207, 1969, 1956, 3845, 2902 128 236 8465 4.483580508474576 5.48770320529357E-4 2.287168083858159E-5 1.4630467904841665E-5

GOTERM_BP_DIRECT GO:0000165~MAPK cascade 11 4.545454545454546 2.332702917542232E-6 367, 1234, 4771, 7124, 4893, 5698, 3265, 5788, 9978, 1956, 3845 183 157 19414 7.432877379833629 0.004828668514647583 2.4201792769500656E-4 2.289547913567701E-4

KEGG_PATHWAY hsa01521:EGFR tyrosine kinase inhibitor resistance 10 4.132231404958678 2.4609969872503584E-6 598, 2247, 7422, 3791, 4893, 3265, 207, 2064, 1956, 3845 128 79 8465 8.371242088607595 6.076822900992207E-4 2.4314650234033543E-5 1.5553500959422265E-5

KEGG_PATHWAY hsa05170:Human immunodeficiency virus 1 infection 15 6.198347107438017 3.170209467614462E-6 7099, 7124, 4893, 3265, 3440, 891, 3106, 3105, 598, 983, 3456, 1234, 207, 9978, 3845 128 212 8465 4.679208431603773 7.827364815562454E-4 3.011698994233739E-5 1.9265119072426347E-5

KEGG_PATHWAY hsa04010:MAPK signaling pathway 17 7.024793388429752 1.0511966373914618E-5 3791, 7422, 7124, 7157, 4893, 3265, 2064, 2250, 7042, 1436, 2247, 2323, 2321, 207, 1969, 1956, 3845 128 302 8465 3.7227079884105962 0.00259310143038316 9.616502571692262E-5 6.151446989179665E-5

KEGG_PATHWAY hsa05218:Melanoma 9 3.71900826446281 1.1016533195958019E-5 2247, 7157, 4893, 3265, 207, 2250, 1956, 3845, 4193 128 72 8465 8.2666015625 0.00271739985464281 9.718156069291537E-5 6.216472303433454E-5

KEGG_PATHWAY hsa04015:Rap1 signaling pathway 14 5.785123966942149 1.465806914213428E-5 3791, 7422, 4893, 3265, 3397, 2250, 1436, 2247, 2321, 207, 1969, 1956, 3845, 2902 128 210 8465 4.408854166666666 0.003614023254189247 1.248463130381782E-4 7.986120429162814E-5

KEGG_PATHWAY hsa04218:Cellular senescence 12 4.958677685950414 2.070723363361282E-5 891, 3106, 3105, 983, 7157, 4893, 3265, 207, 7042, 2308, 3845, 4193 128 156 8465 5.087139423076923 0.005101681655071633 1.649898937903989E-4 1.0554009400357501E-4

KEGG_PATHWAY hsa05163:Human cytomegalovirus infection 14 5.785123966942149 3.048922104066294E-5 7422, 7124, 7157, 4893, 3265, 3440, 4193, 3106, 3105, 3456, 1234, 207, 1956, 3845 128 225 8465 4.1149305555555555 0.007502665834814515 2.3533867490761707E-4 1.5054052888827327E-4

KEGG_PATHWAY hsa05165:Human papillomavirus infection 17 7.024793388429752 3.27388534205234E-5 7015, 7422, 7124, 7157, 4893, 3265, 3440, 4193, 3106, 3105, 3456, 3696, 207, 10134, 2308, 1956, 3845 128 331 8465 3.3965492824773413 0.008054020341106138 2.450453574202812E-4 1.567496618315969E-4

UP_SEQ_FEATURE CROSSLNK:Glycyl lysine isopeptide (Lys-Gly) (interchain with G-Cter in ubiquitin) 12 4.958677685950414 3.8971941484558083E-5 11009, 4904, 5133, 367, 7157, 4893, 80312, 3123, 3265, 207, 1956, 3845 188 270 20543 4.856501182033097 0.053432064654266864 0.006863933193967792 0.0067275313987718395

KEGG_PATHWAY hsa05235:PD-L1 expression and PD-1 checkpoint pathway in cancer 9 3.71900826446281 5.2537551932833674E-5 7099, 3458, 5133, 4893, 3265, 207, 54106, 1956, 3845 128 89 8465 6.687587780898876 0.012893276508372686 3.5072365749756534E-4 2.2434954609156E-4

KEGG_PATHWAY hsa05211:Renal cell carcinoma 8 3.3057851239669422 7.226895129862221E-5 7422, 4893, 3265, 207, 7428, 9978, 7042, 3845 128 69 8465 7.667572463768116 0.017692689417706076 4.697481834410444E-4 3.0048669224163975E-4

KEGG_PATHWAY hsa05230:Central carbon metabolism in cancer 8 3.3057851239669422 7.930886895203224E-5 3417, 7157, 4893, 3265, 207, 2064, 1956, 3845 128 70 8465 7.558035714285714 0.019399429005731106 4.897322657787991E-4 3.132700323605274E-4

UP_KW_DISEASE KW-0656~Proto-oncogene 13 5.371900826446281 9.831647247902548E-5 9232, 5077, 4893, 3265, 2130, 2250, 4193, 1436, 2521, 207, 2308, 1956, 3845 68 232 4623 3.8095207910750504 0.004414685265493934 0.004424241261556146 0.004424241261556146

KEGG_PATHWAY hsa04650:Natural killer cell mediated cytotoxicity 10 4.132231404958678 1.1577041247542552E-4 1437, 3458, 8743, 3456, 7124, 4893, 3265, 3440, 3383, 3845 128 127 8465 5.207308070866142 0.02819192454482322 6.974461434495147E-4 4.4613963831993247E-4

KEGG_PATHWAY hsa04933:AGE-RAGE signaling pathway in diabetic complications 9 3.71900826446281 1.2063208750433497E-4 7422, 7124, 4893, 3265, 207, 3383, 7042, 2308, 3845 128 100 8465 5.951953125 0.029358341858169212 7.094315622278748E-4 4.5380642442106964E-4

KEGG_PATHWAY hsa05212:Pancreatic cancer 8 3.3057851239669422 1.3426476067977653E-4 598, 7422, 7157, 207, 2064, 7042, 1956, 3845 128 76 8465 6.961348684210526 0.03262167322396503 7.444796252017986E-4 4.7622583312503715E-4

KEGG_PATHWAY hsa05220:Chronic myeloid leukemia 8 3.3057851239669422 1.3426476067977653E-4 598, 7157, 4893, 3265, 207, 7042, 3845, 4193 128 76 8465 6.961348684210526 0.03262167322396503 7.444796252017986E-4 4.7622583312503715E-4

KEGG_PATHWAY hsa04068:FoxO signaling pathway 10 4.132231404958678 1.4685551711652515E-4 891, 8743, 4893, 3265, 207, 7042, 2308, 1956, 3845, 4193 128 131 8465 5.048306297709924 0.03562588779542697 7.717726112293981E-4 4.936845043491697E-4

KEGG_PATHWAY hsa05161:Hepatitis B 11 4.545454545454546 1.5257726577580136E-4 7099, 332, 3456, 7124, 7157, 4893, 3265, 207, 3440, 7042, 3845 128 162 8465 4.490499614197531 0.03698805180489906 7.851371801379778E-4 5.022334998453461E-4

KEGG_PATHWAY hsa04210:Apoptosis 10 4.132231404958678 1.9524496985363926E-4 332, 598, 8743, 7124, 7157, 4893, 3563, 3265, 207, 3845 128 136 8465 4.862706801470588 0.04708561437237013 9.841940317112019E-4 6.295654129974491E-4

KEGG_PATHWAY hsa05213:Endometrial cancer 7 2.8925619834710745 2.1655430603647704E-4 7157, 4893, 3265, 207, 2064, 1956, 3845 128 58 8465 7.981546336206896 0.052089040820589294 0.0010488022272747025 6.708937324267329E-4

UP_SEQ_FEATURE REGION:Hypervariable region 3 1.2396694214876034 2.457840754738836E-4 4893, 3265, 3845 188 3 20543 109.27127659574467 0.29273674546584993 0.023087317489513464 0.02262852054862888

KEGG_PATHWAY hsa05210:Colorectal cancer 8 3.3057851239669422 2.9155208496433954E-4 332, 7157, 4893, 3265, 207, 7042, 1956, 3845 128 86 8465 6.15188953488372 0.0694913109044657 0.0013587427355885257 8.691552721578423E-4

KEGG_PATHWAY hsa05221:Acute myeloid leukemia 7 2.8925619834710745 4.7835115539854615E-4 1437, 1436, 3728, 4893, 3265, 207, 3845 128 67 8465 6.909398320895522 0.11146479005525822 0.002148231552426198 0.0013741724100540052

UP_SEQ_FEATURE CARBOHYD:(Microbial infection) O-linked (Glc) threonine; by P.sordellii toxin TcsL 3 1.2396694214876034 4.886187731827289E-4 4893, 3265, 3845 188 4 20543 81.9534574468085 0.49773735776568795 0.036234939548129744 0.03551486977712361

KEGG_PATHWAY hsa05160:Hepatitis C 10 4.132231404958678 5.665795273594507E-4 3458, 3456, 7124, 7157, 4893, 3265, 207, 3440, 1956, 3845 128 157 8465 4.212281050955414 0.1306285530944602 0.002499020415317577 0.0015985636664784502

OMIM_DISEASE 163200~Schimmelpenning-Feuerstein-Mims syndrome, somatic mosaic 3 1.2396694214876034 5.989373149206986E-4 4893, 3265, 3845 84 3 5812 69.19047619047618 0.11716440416979235 0.12457896150350531 0.12457896150350531

KEGG_PATHWAY hsa01522:Endocrine resistance 8 3.3057851239669422 6.471359397806296E-4 7157, 4893, 3265, 207, 2064, 1956, 3845, 4193 128 98 8465 5.39859693877551 0.14776615119799463 0.0028042557390493945 0.0017938154120234996

KEGG_PATHWAY hsa05223:Non-small cell lung cancer 7 2.8925619834710745 7.045944947044041E-4 7157, 4893, 3265, 207, 2064, 1956, 3845 128 72 8465 6.429578993055556 0.15978391905293599 0.0029497430541014884 0.0018868801722592516

KEGG_PATHWAY hsa01524:Platinum drug resistance 7 2.8925619834710745 7.583917917756599E-4 7153, 332, 598, 7157, 207, 2064, 4193 128 73 8465 6.341502568493151 0.17088286785528206 0.003122046209476466 0.0019970983850092377

KEGG_PATHWAY hsa05214:Glioma 7 2.8925619834710745 8.754767075959867E-4 7157, 4893, 3265, 207, 1956, 3845, 4193 128 75 8465 6.172395833333333 0.1945365555302021 0.0035449630619050613 0.002267628193445343

KEGG_PATHWAY hsa04625:C-type lectin receptor signaling pathway 8 3.3057851239669422 9.227342423398938E-4 7124, 4893, 3265, 207, 3593, 3592, 3845, 4193 128 104 8465 5.087139423076923 0.20389212044421168 0.0036760541589992545 0.0023514840369306973

KEGG_PATHWAY hsa05207:Chemical carcinogenesis - receptor activation 11 4.545454545454546 0.0012803490572288775 332, 2247, 7422, 367, 4893, 3265, 207, 2250, 1956, 3845, 7184 128 212 8465 3.431419516509434 0.2712677095495737 0.004746485813462782 0.003036213597275788

KEGG_PATHWAY hsa05224:Breast cancer 9 3.71900826446281 0.0016068464887081936 2247, 7157, 4893, 3265, 207, 2064, 2250, 1956, 3845 128 147 8465 4.048947704081632 0.3278073488121227 0.005836633569278291 0.0037335550767043325

KEGG_PATHWAY hsa04012:ErbB signaling pathway 7 2.8925619834710745 0.001683655378098526 27, 4893, 3265, 207, 2064, 1956, 3845 128 85 8465 5.446231617647058 0.3404604617216014 0.0060269982375411 0.0038553268078198133

KEGG_PATHWAY hsa04370:VEGF signaling pathway 6 2.479338842975207 0.0018398247965677886 7422, 3791, 4893, 3265, 207, 3845 128 59 8465 6.725370762711864 0.36546020821658565 0.00649195321074634 0.004152747397967295

GOTERM_BP_DIRECT GO:0071480~cellular response to gamma radiation 4 1.6528925619834711 0.0027311552555872843 598, 7157, 3265, 4193 183 30 19414 14.144990892531878 0.9965689886411229 0.05247358477170014 0.049641275617757774

BIOCARTA h_telPathway:Telomeres, Telomerase, Cellular Aging, and Immortality 5 2.066115702479339 0.003293304769007411 7015, 7157, 207, 1956, 3845 60 18 1623 7.513888888888888 0.4459356225959652 0.09825025894205443 0.09331030178854331

KEGG_PATHWAY hsa04664:Fc epsilon RI signaling pathway 6 2.479338842975207 0.0034419018170934847 1437, 7124, 4893, 3265, 207, 3845 128 68 8465 5.835248161764706 0.5732752898810789 0.011973940124254798 0.007659443480292544

KEGG_PATHWAY hsa05225:Hepatocellular carcinoma 9 3.71900826446281 0.0036920440286747755 598, 7015, 7157, 4893, 3265, 207, 7042, 1956, 3845 128 168 8465 3.542829241071429 0.5989312748839981 0.012665762153925965 0.008101985507369647

OMIM_DISEASE 114480~Breast cancer, somatic 3 1.2396694214876034 0.005335872700961063 7157, 207, 3845 84 8 5812 25.94642857142857 0.6713734032906744 0.5549307608999505 0.5549307608999505

GOTERM_BP_DIRECT GO:0071364~cellular response to epidermal growth factor stimulus 4 1.6528925619834711 0.008095830969332023 3397, 207, 2064, 1956 183 44 19414 9.644311972180825 0.9999999527219089 0.12443592045454775 0.11771937920591675

KEGG_PATHWAY hsa04919:Thyroid hormone signaling pathway 7 2.8925619834710745 0.009568969500449309 7157, 4893, 3265, 207, 2308, 3845, 4193 128 121 8465 3.825865185950413 0.9069777369559687 0.029918170463430115 0.019137939000898618

KEGG_PATHWAY hsa04211:Longevity regulating pathway 6 2.479338842975207 0.01068887915843611 7157, 4893, 3265, 207, 2308, 3845 128 89 8465 4.458391853932584 0.929656482906733 0.03259448335967555 0.020849912432505006

GOTERM_BP_DIRECT GO:0007265~Ras protein signal transduction 5 2.066115702479339 0.011423948772942725 2247, 7157, 4893, 3265, 3845 183 93 19414 5.703625359891885 0.9999999999557486 0.1569847265156037 0.14851133404825542

KEGG_PATHWAY hsa05203:Viral carcinogenesis 9 3.71900826446281 0.011519677203929827 3106, 3105, 983, 1234, 7157, 4893, 3265, 3845, 4193 128 204 8465 2.9176240808823533 0.9428385778121511 0.034281449028562255 0.021929024074950752

BIOCARTA h_tffPathway:Trefoil Factors Initiate Mucosal Healing 5 2.066115702479339 0.012944034368984891 4583, 3265, 207, 2064, 1956 60 26 1623 5.201923076923077 0.9029091724079747 0.23169821520482953 0.22004858427274315

KEGG_PATHWAY hsa04213:Longevity regulating pathway - multiple species 5 2.066115702479339 0.013794507562252487 4893, 3265, 207, 2308, 3845 128 62 8465 5.333291330645162 0.9676442124758636 0.03916371687214212 0.02505209419351601

UP_SEQ_FEATURE LIPID:S-palmitoyl cysteine 8 3.3057851239669422 0.015469836545573056 4582, 1234, 4893, 931, 3265, 925, 1956, 3845 188 284 20543 3.0780641294575966 0.9999999997117972 0.40674206681529224 0.39865918685019064

KEGG_PATHWAY hsa05216:Thyroid cancer 4 1.6528925619834711 0.01767705700782002 7157, 4893, 3265, 3845 128 37 8465 7.149493243243244 0.9877873753740536 0.04860644675757651 0.03109238294614206

KEGG_PATHWAY hsa04916:Melanogenesis 6 2.479338842975207 0.017710851045270794 7299, 4893, 3265, 7306, 1638, 3845 128 101 8465 3.9286819306930694 0.9878907122499802 0.04860644675757651 0.03109238294614206

KEGG_PATHWAY hsa04550:Signaling pathways regulating pluripotency of stem cells 7 2.8925619834710745 0.020411564749301826 2247, 4893, 3265, 3397, 207, 6657, 3845 128 143 8465 3.2372705419580416 0.993865468755982 0.05480061405519077 0.03505464380858357

KEGG_PATHWAY hsa04137:Mitophagy - animal 5 2.066115702479339 0.022704773244344435 598, 7157, 4893, 3265, 3845 128 72 8465 4.592556423611112 0.9965615154878518 0.05966041480162846 0.038163342261770435

INTERPRO IPR020849:Small GTPase superfamily, Ras type 3 1.2396694214876034 0.02336347182964464 4893, 3265, 3845 183 25 19144 12.55344262295082 0.9999577095260037 0.3110262187321443 0.30153480830135115

GOTERM_BP_DIRECT GO:0008286~insulin receptor signaling pathway 4 1.6528925619834711 0.025055656503600594 3265, 207, 3484, 2308 183 67 19414 6.333578011581437 1.0 0.26661788330754477 0.25222694213624597

KEGG_PATHWAY hsa04510:Focal adhesion 8 3.3057851239669422 0.032842200679731134 7422, 3791, 2321, 3696, 3265, 207, 2064, 1956 128 203 8465 2.60621921182266 0.9997382765829108 0.08450024549889155 0.05405278861872416

KEGG_PATHWAY hsa04071:Sphingolipid signaling pathway 6 2.479338842975207 0.035175480600053516 7124, 7157, 4893, 3265, 207, 3845 128 121 8465 3.2793130165289255 0.9998558754529325 0.08957055369291977 0.057296143657819126

GOTERM_BP_DIRECT GO:0051402~neuron apoptotic process 4 1.6528925619834711 0.03695324189960536 598, 7157, 3265, 3845 183 78 19414 5.440381112512259 1.0 0.33505821910378797 0.31697314896420997

KEGG_PATHWAY hsa04662:B cell receptor signaling pathway 5 2.066115702479339 0.03717817201206075 4893, 3265, 207, 11027, 3845 128 84 8465 3.9364769345238098 0.9999137331178378 0.09370416823447963 0.059940318141893864

UP_KW_PTM KW-0564~Palmitate 10 4.132231404958678 0.04084288222292469 4582, 367, 1234, 4893, 931, 6007, 3265, 925, 1956, 3845 172 378 14111 2.1703888273655716 0.6167657268770441 0.1400327390500275 0.11669394920835625

BBID 65.Integrin_affinity_modulation 3 1.2396694214876034 0.04097411022800451 4893, 3265, 3845 28 5 388 8.314285714285715 0.9418616714154062 0.6570232038443005 0.6570232038443005

KEGG_PATHWAY hsa04540:Gap junction 5 2.066115702479339 0.04295221288450336 983, 4893, 3265, 1956, 3845 128 88 8465 3.757546164772727 0.999980474373155 0.10665942189303758 0.06822748444979732

KEGG_PATHWAY hsa05206:MicroRNAs in cancer 10 4.132231404958678 0.04318195218341602 7422, 7157, 4893, 3265, 900, 2064, 7042, 1956, 3845, 4193 128 310 8465 2.1333165322580645 0.9999815985707537 0.10665942189303758 0.06822748444979732

KEGG_PATHWAY hsa04926:Relaxin signaling pathway 6 2.479338842975207 0.04437241295429432 7422, 4893, 3265, 207, 1956, 3845 128 129 8465 3.0759447674418605 0.9999864698197831 0.10851471286842274 0.06941426977008418

KEGG_PATHWAY hsa04915:Estrogen signaling pathway 6 2.479338842975207 0.056296376110281404 4893, 3265, 207, 1956, 3845, 7184 128 138 8465 2.8753396739130435 0.9999993913062744 0.1340805586937689 0.08576813066241087

KEGG_PATHWAY hsa04810:Regulation of actin cytoskeleton 8 3.3057851239669422 0.0564549720815869 2247, 4893, 3696, 3265, 207, 2250, 1956, 3845 128 229 8465 2.310316593886463 0.9999994160579971 0.1340805586937689 0.08576813066241087

KEGG_PATHWAY hsa05231:Choline metabolism in cancer 5 2.066115702479339 0.05947480531435403 4893, 3265, 207, 1956, 3845 128 98 8465 3.3741230867346936 0.9999997354564562 0.13858751804382494 0.08865112490252769

KEGG_PATHWAY hsa04062:Chemokine signaling pathway 7 2.8925619834710745 0.06862287534500353 6375, 6366, 1234, 4893, 3265, 207, 3845 128 192 8465 2.411092122395833 0.9999999763399816 0.15840981504874646 0.10133097480850987

KEGG_PATHWAY hsa04929:GnRH secretion 4 1.6528925619834711 0.07102946609525711 4893, 3265, 207, 3845 128 64 8465 4.13330078125 0.9999999875124028 0.1624470196808195 0.10391347817639467

GOTERM_BP_DIRECT GO:0042981~regulation of apoptotic process 6 2.479338842975207 0.07899982937828454 332, 598, 4771, 7157, 27, 207 183 243 19414 2.6194427578762736 1.0 0.5174300621080736 0.4895013069485053

KEGG_PATHWAY hsa04720:Long-term potentiation 4 1.6528925619834711 0.07912248329058627 4893, 3265, 3845, 2902 128 67 8465 3.948227611940298 0.9999999985616591 0.17929590250252117 0.11469130605424431

KEGG_PATHWAY hsa04917:Prolactin signaling pathway 4 1.6528925619834711 0.08759904345908316 4893, 3265, 207, 3845 128 70 8465 3.779017857142857 0.9999999998534871 0.19147755517162426 0.12248361828792159

KEGG_PATHWAY hsa04722:Neurotrophin signaling pathway 5 2.066115702479339 0.10351313309750103 7157, 4893, 3265, 207, 3845 128 119 8465 2.778689600840336 0.9999999999981017 0.21852772542805773 0.139786966063292

UP_SEQ_FEATURE LIPID:S-farnesyl cysteine 3 1.2396694214876034 0.10363856203606953 4893, 3265, 3845 188 60 20543 5.463563829787233 1.0 1.0 0.9808238636363636

KEGG_PATHWAY hsa04935:Growth hormone synthesis, secretion and action 5 2.066115702479339 0.10590609078002676 1442, 4893, 3265, 207, 3845 128 120 8465 2.7555338541666665 0.9999999999990191 0.21982188590476145 0.14061480960709435

KEGG_PATHWAY hsa05208:Chemical carcinogenesis - reactive oxygen species 7 2.8925619834710745 0.11838271284306695 7422, 27, 4893, 3265, 207, 1956, 3845 128 223 8465 2.0759178811659194 0.9999999999999695 0.24367108393531278 0.15587057191003814

KEGG_PATHWAY hsa04910:Insulin signaling pathway 5 2.066115702479339 0.15021664319238165 4893, 3265, 207, 2308, 3845 128 137 8465 2.413606295620438 1.0 0.2968280869481461 0.1898738369951704

KEGG_PATHWAY hsa04140:Autophagy - animal 5 2.066115702479339 0.1615495472973968 598, 4893, 3265, 207, 3845 128 141 8465 2.3451351950354606 1.0 0.31419478883824414 0.2009829013621157

GOTERM_MF_DIRECT GO:0019003~GDP binding 3 1.2396694214876034 0.16164821111040295 4893, 3265, 3845 185 74 18945 4.151570489408328 1.0 1.0 0.9736842105263158

KEGG_PATHWAY hsa04912:GnRH signaling pathway 4 1.6528925619834711 0.16341609141854346 4893, 3265, 1956, 3845 128 93 8465 2.844422043010753 1.0 0.3153419889092206 0.2017167378447646

KEGG_PATHWAY hsa04072:Phospholipase D signaling pathway 5 2.066115702479339 0.1821014740577786 4893, 3265, 207, 1956, 3845 128 148 8465 2.2342166385135136 1.0 0.34599280070977934 0.22132333000868473

BIOCARTA h_chemicalPathway:Apoptotic Signaling in Response to DNA Damage 3 1.2396694214876034 0.1891260698421557 598, 7157, 207 60 22 1623 3.688636363636364 1.0 1.0 0.9550561797752809

KEGG_PATHWAY hsa04921:Oxytocin signaling pathway 5 2.066115702479339 0.20037372347332033 952, 4893, 3265, 1956, 3845 128 154 8465 2.147169237012987 1.0 0.3714703583187782 0.2376207150379229

BIOCARTA h_her2Pathway:Role of ERBB2 in Signal Transduction and Oncology 3 1.2396694214876034 0.202547335053219 3265, 2064, 1956 60 23 1623 3.5282608695652176 1.0 1.0 0.9550561797752809

BIOCARTA h_rasPathway:Ras Signaling Pathway 3 1.2396694214876034 0.202547335053219 598, 3265, 207 60 23 1623 3.5282608695652176 1.0 1.0 0.9550561797752809

KEGG_PATHWAY hsa04150:mTOR signaling pathway 5 2.066115702479339 0.20658581863634817 7124, 4893, 3265, 207, 3845 128 156 8465 2.119641426282051 1.0 0.3751963029645441 0.24000411282752215

UP_SEQ_FEATURE MOTIF:Effector region 3 1.2396694214876034 0.2209860085849833 4893, 3265, 3845 188 97 20543 3.379524018425093 1.0 1.0 0.9808238636363636

KEGG_PATHWAY hsa04730:Long-term depression 3 1.2396694214876034 0.22714521219518224 4893, 3265, 3845 128 60 8465 3.306640625 1.0 0.40952457965116795 0.2619630914367795

KEGG_PATHWAY hsa04725:Cholinergic synapse 4 1.6528925619834711 0.24035359971090603 4893, 3265, 207, 3845 128 113 8465 2.3409845132743365 1.0 0.4301981096274912 0.2751874547414721

BIOCARTA h_p53hypoxiaPathway:Hypoxia and p53 in the Cardiovascular system 3 1.2396694214876034 0.24337311217404997 7157, 207, 4193 60 26 1623 3.1211538461538466 1.0 1.0 0.9550561797752809

KEGG_PATHWAY hsa04360:Axon guidance 5 2.066115702479339 0.29149320519955196 4893, 3265, 1969, 8829, 3845 128 182 8465 1.8168355082417582 1.0 0.5142772977449238 0.3289709030109229

GOTERM_MF_DIRECT GO:0004672~protein kinase activity 6 2.479338842975207 0.3107084831506337 983, 2984, 27, 207, 2064, 1956 185 378 18945 1.6254826254826258 1.0 1.0 0.9736842105263158

KEGG_PATHWAY hsa04371:Apelin signaling pathway 4 1.6528925619834711 0.3467516638707099 4893, 3265, 207, 3845 128 139 8465 1.9031025179856114 1.0 0.6031525420849672 0.385822273884311

INTERPRO IPR001806:Small GTPase superfamily 3 1.2396694214876034 0.39982443814908986 4893, 3265, 3845 183 144 19144 2.179417122040073 1.0 1.0 0.971764705882353

KEGG_PATHWAY hsa05222:Small cell lung cancer 3 1.2396694214876034 0.4030314933443433 598, 7157, 207 128 92 8465 2.1565047554347827 1.0 0.6772025772520599 0.43319031257419216

BIOCARTA h_il2rbPathway:IL-2 Receptor Beta Chain in T cell Activation 3 1.2396694214876034 0.4185503058546992 598, 3265, 207 60 39 1623 2.080769230769231 1.0 1.0 0.9550561797752809

INTERPRO IPR005225:Small GTP-binding protein domain 3 1.2396694214876034 0.4967849794805676 4893, 3265, 3845 183 174 19144 1.8036555492745432 1.0 1.0 0.971764705882353

KEGG_PATHWAY hsa04726:Serotonergic synapse 3 1.2396694214876034 0.5177734238917371 4893, 3265, 3845 128 115 8465 1.725203804347826 1.0 0.8272103717190462 0.5291467155125882

KEGG_PATHWAY hsa05010:Alzheimer disease 7 2.8925619834710745 0.5190996259775391 7124, 4893, 3265, 207, 6868, 3845, 2902 128 384 8465 1.2055460611979165 1.0 0.8272103717190462 0.5291467155125882

UP_KW_LIGAND KW-0342~GTP-binding 5 2.066115702479339 0.5269366475767354 51062, 2984, 4893, 3265, 3845 71 370 6858 1.3052912066996576 0.9999999983295971 1.0 1.0

KEGG_PATHWAY hsa05034:Alcoholism 4 1.6528925619834711 0.5390638196965483 4893, 3265, 3845, 2902 128 188 8465 1.4070811170212767 1.0 0.8535177145195347 0.5459748943080425

GOTERM_MF_DIRECT GO:0005525~GTP binding 5 2.066115702479339 0.5481087972003189 51062, 2984, 4893, 3265, 3845 185 402 18945 1.273699072206535 1.0 1.0 0.9736842105263158

BIOCARTA h_nfatPathway:NFAT and Hypertrophy of the heart (Transcription in the broken heart) 3 1.2396694214876034 0.5932162930198641 2247, 3265, 207 60 54 1623 1.502777777777778 1.0 1.0 0.9550561797752809

UP_KW_PTM KW-0636~Prenylation 3 1.2396694214876034 0.6260644219901886 4893, 3265, 3845 172 174 14111 1.414494787489976 0.9999999998506084 0.883855654574384 0.7365463788119867

GOTERM_MF_DIRECT GO:0003924~GTPase activity 4 1.6528925619834711 0.6788503538221766 51062, 4893, 3265, 3845 185 358 18945 1.1441944738034124 1.0 1.0 0.9736842105263158

KEGG_PATHWAY hsa05022:Pathways of neurodegeneration - multiple diseases 7 2.8925619834710745 0.7265506626941672 598, 7124, 2521, 4893, 3265, 3845, 2902 128 476 8465 0.9725413602941176 1.0 1.0 0.7265506626941672

KEGG_PATHWAY hsa04714:Thermogenesis 3 1.2396694214876034 0.8676988822398525 4893, 3265, 3845 128 232 8465 0.8551656788793103 1.0 1.0 0.8676988822398525

INTERPRO IPR027417:P-loop containing nucleoside triphosphate hydrolase 6 2.479338842975207 0.9404098342937398 51062, 9585, 4343, 4893, 3265, 3845 183 918 19144 0.6837387049537484 1.0 1.0 0.971764705882353

Annotation Cluster 18 Enrichment Score: 2.5726911202298957

Category Term Count % PValue Genes List Total Pop Hits Pop Total Fold Enrichment Bonferroni Benjamini FDR

UP_SEQ_FEATURE PROPEP:Removed in mature form 12 4.958677685950414 8.132681593825434E-5 8000, 2348, 1048, 54742, 4893, 5698, 3265, 2719, 10232, 7070, 1604, 3845 188 293 20543 4.475274126788178 0.10827201593487279 0.012732164850777817 0.012479148090081028

UP_KW_PTM KW-0336~GPI-anchor 8 3.3057851239669422 0.0017848119794753943 8000, 2348, 1048, 54742, 2719, 10232, 7070, 1604 172 143 14111 4.589689380387055 0.04025471038728601 0.014278495835803155 0.011898746529835963

GOTERM_CC_DIRECT GO:0031225~anchored component of membrane 6 2.479338842975207 0.003299443751134603 8000, 1048, 54742, 10232, 7070, 1604 188 110 20624 5.98375241779497 0.6662043971608815 0.04564230522402867 0.04220538464993013

UP_SEQ_FEATURE LIPID:GPI-anchor amidated serine 4 1.6528925619834711 0.015155302338085305 8000, 2348, 10232, 1604 188 57 20543 7.66815976110489 0.9999999995479762 0.40674206681529224 0.39865918685019064

UP_KW_PTM KW-0449~Lipoprotein 20 8.264462809917356 0.01886743331324197 8000, 4582, 7124, 27, 4893, 3265, 7070, 2348, 367, 1048, 1234, 54742, 931, 6007, 2719, 10232, 925, 1604, 1956, 3845 172 937 14111 1.7511354893152318 0.35473679077838505 0.09534791031345986 0.07945659192788322

Annotation Cluster 19 Enrichment Score: 2.550863260317979

Category Term Count % PValue Genes List Total Pop Hits Pop Total Fold Enrichment Bonferroni Benjamini FDR

GOTERM_BP_DIRECT GO:0006959~humoral immune response 8 3.3057851239669422 1.6137768974850249E-6 3458, 5133, 3456, 7124, 931, 3123, 1880, 3440 183 61 19414 13.913105795932994 0.003342989490187409 2.0033259056645704E-4 1.8951945796720731E-4

GOTERM_BP_DIRECT GO:0007259~JAK-STAT cascade 5 2.066115702479339 6.931514596956303E-4 1437, 3458, 3456, 282617, 1438 183 43 19414 12.335747871394078 0.7627849073454955 0.019451565714120392 0.018401649877984736

GOTERM_BP_DIRECT GO:0098586~cellular response to virus 6 2.479338842975207 0.001583030458289585 3458, 51284, 3456, 3440, 3592, 282617 183 90 19414 7.072495446265939 0.9626488249718843 0.035704219575553135 0.033777052061113644

GOTERM_BP_DIRECT GO:0033141~positive regulation of peptidyl-serine phosphorylation of STAT protein 3 1.2396694214876034 0.0163281975213659 3458, 3456, 3440 183 21 19414 15.155347384855585 0.9999999999999986 0.20167267771925146 0.19078721270500754

KEGG_PATHWAY hsa04217:Necroptosis 6 2.479338842975207 0.0906210776938031 7099, 3458, 8743, 3456, 7124, 3440 128 159 8465 2.495577830188679 0.9999999999354366 0.19634566833657338 0.12559763399667448

UP_KW_BIOLOGICAL_PROCESS KW-0051~Antiviral defense 4 1.6528925619834711 0.1890131687739996 3458, 3456, 3440, 282617 125 136 11262 2.6498823529411766 0.999999472827979 1.0 0.9159868948278441

Annotation Cluster 20 Enrichment Score: 2.497710549368475

Category Term Count % PValue Genes List Total Pop Hits Pop Total Fold Enrichment Bonferroni Benjamini FDR

GOTERM_BP_DIRECT GO:0001570~vasculogenesis 8 3.3057851239669422 2.0145444959083794E-6 7422, 3791, 6862, 3696, 2022, 1969, 7490, 8829 183 63 19414 13.471419897649406 0.004171459231583685 2.322322127227715E-4 2.1969726919267494E-4

GOTERM_BP_DIRECT GO:0045766~positive regulation of angiogenesis 11 4.545454545454546 3.4531725398535566E-6 3728, 1116, 2247, 7015, 7422, 3791, 2321, 3696, 2022, 958, 8829 183 164 19414 7.115620418499266 0.0071397355087851055 3.11536218269397E-4 2.9472076937967526E-4

GOTERM_MF_DIRECT GO:0019838~growth factor binding 6 2.479338842975207 2.7724468002949136E-5 1436, 3791, 2321, 2064, 1969, 8829 185 37 18945 16.60628195763331 0.01259032773655211 0.0012670081877347757 0.0012309663793309417

GOTERM_BP_DIRECT GO:0002042~cell migration involved in sprouting angiogenesis 5 2.066115702479339 3.947900249084252E-5 2247, 7422, 3791, 207, 8829 183 21 19414 25.258912308092636 0.07865484080488305 0.0021557613202236375 0.00203940215498747

GOTERM_BP_DIRECT GO:0043536~positive regulation of blood vessel endothelial cell migration 6 2.479338842975207 9.355362362090386E-5 2247, 7422, 3791, 207, 958, 6868 183 49 19414 12.990297758447642 0.17645151147427873 0.003961709571701541 0.003747872717710904

GOTERM_BP_DIRECT GO:0014068~positive regulation of phosphatidylinositol 3-kinase signaling 7 2.8925619834710745 1.5790273470103676E-4 2247, 7422, 3791, 7124, 2321, 7042, 780 183 86 19414 8.635023509975856 0.2794021364543323 0.006300926432781756 0.005960828234964138

GOTERM_BP_DIRECT GO:0035924~cellular response to vascular endothelial growth factor stimulus 5 2.066115702479339 2.781524268387282E-4 7422, 3791, 2321, 207, 8829 183 34 19414 15.601092896174864 0.43855786850729706 0.009619438094839351 0.009100220231407058

GOTERM_BP_DIRECT GO:0001938~positive regulation of endothelial cell proliferation 6 2.479338842975207 6.958728947224013E-4 2247, 7422, 3791, 4893, 207, 8829 183 75 19414 8.486994535519125 0.7641216063237166 0.019451565714120392 0.018401649877984736

UP_KW_BIOLOGICAL_PROCESS KW-0037~Angiogenesis 8 3.3057851239669422 8.868910236179064E-4 4240, 2247, 7422, 3791, 2321, 2022, 1969, 8829 125 140 11262 5.1483428571428576 0.05938619572394721 0.014364254426815805 0.013115188824483991

GOTERM_BP_DIRECT GO:0051894~positive regulation of focal adhesion assembly 4 1.6528925619834711 0.0017977190724351795 7422, 3791, 7070, 8829 183 26 19414 16.32114333753678 0.9760939588773558 0.03845636160106183 0.03638064473392018

KEGG_PATHWAY hsa04370:VEGF signaling pathway 6 2.479338842975207 0.0018398247965677886 7422, 3791, 4893, 3265, 207, 3845 128 59 8465 6.725370762711864 0.36546020821658565 0.00649195321074634 0.004152747397967295

GOTERM_MF_DIRECT GO:0005021~vascular endothelial growth factor-activated receptor activity 3 1.2396694214876034 0.002527835500072992 3791, 2321, 8829 185 8 18945 38.40202702702703 0.6854723825651887 0.0679541660901975 0.06602111541367109

GOTERM_BP_DIRECT GO:0048010~vascular endothelial growth factor receptor signaling pathway 4 1.6528925619834711 0.0027311552555872843 7422, 3791, 2321, 8829 183 30 19414 14.144990892531878 0.9965689886411229 0.05247358477170014 0.049641275617757774

GOTERM_BP_DIRECT GO:0001525~angiogenesis 9 3.71900826446281 0.0028575709249408377 4162, 4240, 7422, 3791, 2321, 634, 3397, 7070, 8829 183 255 19414 3.7442622950819673 0.9973625632700603 0.054398712561947137 0.05146249289595289

GOTERM_BP_DIRECT GO:0001569~branching involved in blood vessel morphogenesis 4 1.6528925619834711 0.0032911374647962835 7422, 3791, 2022, 8829 183 32 19414 13.260928961748634 0.9989302953829511 0.060434603888958306 0.05717259153446995

GOTERM_BP_DIRECT GO:0010595~positive regulation of endothelial cell migration 5 2.066115702479339 0.003616720789616739 2247, 7422, 3791, 207, 8829 183 67 19414 7.916972514476796 0.9994569448316323 0.06525822294308464 0.0617358513914579

GOTERM_BP_DIRECT GO:0090050~positive regulation of cell migration involved in sprouting angiogenesis 4 1.6528925619834711 0.004254515431696443 2247, 7422, 3791, 8829 183 35 19414 12.124277907884466 0.9998561806618629 0.07356766267308433 0.06959678160350098

GOTERM_BP_DIRECT GO:0038084~vascular endothelial growth factor signaling pathway 3 1.2396694214876034 0.009620637669595603 7422, 3791, 2321 183 16 19414 19.891393442622952 0.9999999980578517 0.13767464251317846 0.1302435292787322

GOTERM_BP_DIRECT GO:0048844~artery morphogenesis 3 1.2396694214876034 0.02276324313633832 7422, 2022, 8829 183 25 19414 12.730491803278689 1.0 0.24859857635737903 0.2351802435612217

BIOCARTA h_vegfPathway:VEGF, Hypoxia, and Angiogenesis 5 2.066115702479339 0.02379805994852529 7422, 3791, 2321, 3265, 7428 60 31 1623 4.362903225806451 0.9865847031124596 0.3227989706217411 0.3065688547804245

KEGG_PATHWAY hsa04510:Focal adhesion 8 3.3057851239669422 0.032842200679731134 7422, 3791, 2321, 3696, 3265, 207, 2064, 1956 128 203 8465 2.60621921182266 0.9997382765829108 0.08450024549889155 0.05405278861872416

GOTERM_BP_DIRECT GO:0050918~positive chemotaxis 3 1.2396694214876034 0.07448730626530087 2247, 7422, 8829 183 48 19414 6.630464480874317 1.0 0.49538833493749773 0.46864930191918464

BIOCARTA h_no1Pathway:Actions of Nitric Oxide in the Heart 4 1.6528925619834711 0.09251540061050084 7422, 3791, 2321, 207 60 30 1623 3.6066666666666665 0.999999971605896 0.7527389413308933 0.7148917319902338

GOTERM_MF_DIRECT GO:0008201~heparin binding 5 2.066115702479339 0.09567446463465278 7123, 2247, 7422, 5788, 8829 185 178 18945 2.8765563316125116 1.0 0.8249666101516285 0.8014992886374687

UP_KW_MOLECULAR_FUNCTION KW-0358~Heparin-binding 3 1.2396694214876034 0.27613479765792054 2247, 7422, 8829 130 94 11749 2.8843698854337156 0.9999999985597593 1.0 0.967741935483871

UP_KW_MOLECULAR_FUNCTION KW-9996~Developmental protein 14 5.785123966942149 0.3082528416154039 5077, 3791, 7422, 6862, 3397, 55553, 2247, 54894, 2321, 80312, 207, 6657, 8829, 1956 130 1004 11749 1.260235979160282 0.999999999917456 1.0 0.967741935483871

UP_KW_MOLECULAR_FUNCTION KW-0217~Developmental protein 14 5.785123966942149 0.3082528416154039 5077, 3791, 7422, 6862, 3397, 55553, 2247, 54894, 2321, 80312, 207, 6657, 8829, 1956 130 1004 11749 1.260235979160282 0.999999999917456 1.0 0.967741935483871

UP_KW_BIOLOGICAL_PROCESS KW-0221~Differentiation 9 3.71900826446281 0.613487482923693 55553, 2247, 7422, 3791, 2321, 23532, 1969, 8829, 2308 125 766 11262 1.0585691906005221 1.0 1.0 0.926470588235294

Annotation Cluster 21 Enrichment Score: 2.4587441662033216

Category Term Count % PValue Genes List Total Pop Hits Pop Total Fold Enrichment Bonferroni Benjamini FDR

GOTERM_BP_DIRECT GO:0002250~adaptive immune response 14 5.785123966942149 9.673105772486053E-4 5133, 8995, 3123, 1880, 3440, 1493, 3106, 3458, 57823, 3105, 3456, 942, 925, 170482 183 504 19414 2.9468731026108075 0.8657619889025693 0.024182764431215132 0.02287747786914473

UP_KW_BIOLOGICAL_PROCESS KW-0391~Immunity 22 9.090909090909092 0.0010408880019431742 7099, 4153, 9447, 5133, 8995, 3123, 1880, 1493, 6280, 1436, 3106, 51284, 57823, 3105, 942, 5698, 54106, 11027, 925, 958, 1604, 170482 125 921 11262 2.152130293159609 0.06933758951799918 0.014364254426815805 0.013115188824483991

UP_KW_BIOLOGICAL_PROCESS KW-1064~Adaptive immunity 11 4.545454545454546 0.041763564860753645 3106, 57823, 3105, 5133, 8995, 942, 3123, 1880, 1493, 925, 170482 125 486 11262 2.0392098765432096 0.9473237409635732 0.28816859753920016 0.2631104586227479

Annotation Cluster 22 Enrichment Score: 2.4562985411415017

Category Term Count % PValue Genes List Total Pop Hits Pop Total Fold Enrichment Bonferroni Benjamini FDR

UP_KW_BIOLOGICAL_PROCESS KW-0391~Immunity 22 9.090909090909092 0.0010408880019431742 7099, 4153, 9447, 5133, 8995, 3123, 1880, 1493, 6280, 1436, 3106, 51284, 57823, 3105, 942, 5698, 54106, 11027, 925, 958, 1604, 170482 125 921 11262 2.152130293159609 0.06933758951799918 0.014364254426815805 0.013115188824483991

UP_KW_BIOLOGICAL_PROCESS KW-0399~Innate immunity 13 5.371900826446281 0.0019280998857460442 7099, 4153, 9447, 6280, 1436, 3106, 51284, 57823, 3105, 54106, 11027, 1604, 170482 125 412 11262 2.842834951456311 0.12468137263726964 0.02217314868607951 0.02024504880033346

UP_KW_BIOLOGICAL_PROCESS KW-0395~Inflammatory response 8 3.3057851239669422 0.004851915749409366 7099, 1436, 1116, 51284, 9447, 6366, 54106, 6280 125 189 11262 3.813587301587302 0.28508883648085614 0.04782602667274947 0.04366724174468429

GOTERM_BP_DIRECT GO:0045087~innate immune response 13 5.371900826446281 0.015358862520623295 7099, 4153, 9447, 6280, 282617, 1436, 3106, 51284, 3105, 3456, 54106, 11027, 1604 183 627 19414 2.1995799234798374 0.9999999999999888 0.1943270715261789 0.183838092243802

Annotation Cluster 23 Enrichment Score: 2.303801498178574

Category Term Count % PValue Genes List Total Pop Hits Pop Total Fold Enrichment Bonferroni Benjamini FDR

GOTERM_BP_DIRECT GO:2001240~negative regulation of extrinsic apoptotic signaling pathway in absence of ligand 5 2.066115702479339 3.8754076569977634E-4 1437, 598, 7015, 7124, 207 183 37 19414 14.33613941810663 0.552600321707176 0.012764239505191045 0.012075278143946998

KEGG_PATHWAY hsa04664:Fc epsilon RI signaling pathway 6 2.479338842975207 0.0034419018170934847 1437, 7124, 4893, 3265, 207, 3845 128 68 8465 5.835248161764706 0.5732752898810789 0.011973940124254798 0.007659443480292544

KEGG_PATHWAY hsa04668:TNF signaling pathway 5 2.066115702479339 0.09193473633655265 1437, 3456, 7124, 207, 3383 128 114 8465 2.900561951754386 0.9999999999548232 0.19745982500111744 0.1263103334015245

Annotation Cluster 24 Enrichment Score: 2.274946853547278

Category Term Count % PValue Genes List Total Pop Hits Pop Total Fold Enrichment Bonferroni Benjamini FDR

KEGG_PATHWAY hsa01524:Platinum drug resistance 7 2.8925619834710745 7.583917917756599E-4 7153, 332, 598, 7157, 207, 2064, 4193 128 73 8465 6.341502568493151 0.17088286785528206 0.003122046209476466 0.0019970983850092377

GOTERM_MF_DIRECT GO:0019899~enzyme binding 11 4.545454545454546 0.004097905074545211 3329, 7153, 332, 367, 7157, 207, 7428, 958, 1956, 7184, 4193 185 380 18945 2.964366998577525 0.8468887189765093 0.1040412566148423 0.10108165850544854

GOTERM_CC_DIRECT GO:0032991~macromolecular complex 12 4.958677685950414 0.04816193435790586 3329, 7153, 354, 332, 367, 7124, 7157, 207, 1956, 6382, 7184, 4193 188 685 20624 1.921789097685976 0.999999923627166 0.32632167769030096 0.30174926220157344

Annotation Cluster 25 Enrichment Score: 2.215254420066804

Category Term Count % PValue Genes List Total Pop Hits Pop Total Fold Enrichment Bonferroni Benjamini FDR

GOTERM_BP_DIRECT GO:0030307~positive regulation of cell growth 8 3.3057851239669422 2.538740941359068E-5 952, 207, 3484, 2064, 6868, 6280, 7042, 1956 183 92 19414 9.224994060346877 0.05131602402557267 0.0014633020703666848 0.0013843190188577363

GOTERM_BP_DIRECT GO:1900087~positive regulation of G1/S transition of mitotic cell cycle 4 1.6528925619834711 0.01213777531060293 7015, 207, 6868, 1956 183 51 19414 8.320582877959927 0.9999999999901148 0.16041964184395593 0.15176084671792073

GOTERM_BP_DIRECT GO:0045737~positive regulation of cyclin-dependent protein serine/threonine kinase activity 3 1.2396694214876034 0.05404179589659606 207, 6868, 1956 183 40 19414 7.95655737704918 1.0 0.42547622018814024 0.4025107567370214

GOTERM_BP_DIRECT GO:0007173~epidermal growth factor receptor signaling pathway 3 1.2396694214876034 0.08269801137989001 207, 6868, 1956 183 51 19414 6.240437158469946 1.0 0.5215756036877561 0.49342308917545313

Annotation Cluster 26 Enrichment Score: 2.1976609465043375

Category Term Count % PValue Genes List Total Pop Hits Pop Total Fold Enrichment Bonferroni Benjamini FDR

GOTERM_CC_DIRECT GO:0005796~Golgi lumen 8 3.3057851239669422 5.469983494609397E-5 4582, 94025, 4583, 4584, 4585, 2719, 727897, 6382 188 107 20624 8.202028236229866 0.017996927588707745 0.001849740201265746 0.001710452535507783

UP_SEQ_FEATURE REPEAT:12 7 2.8925619834710745 1.9570567600189084E-4 4582, 94025, 4583, 4584, 2130, 6490, 5903 188 92 20543 8.314118871415356 0.24101729154785922 0.022979108123888686 0.022522461546550937

UP_SEQ_FEATURE REPEAT:6 8 3.3057851239669422 4.557631161818255E-4 4582, 94025, 4583, 4584, 7428, 2130, 6490, 5903 188 150 20543 5.827801418439717 0.47392769511450905 0.035964061336663126 0.03524937452514676

UP_SEQ_FEATURE REPEAT:5 8 3.3057851239669422 8.320517378371549E-4 4582, 94025, 4583, 4584, 7428, 2130, 6490, 5903 188 166 20543 5.266085619072032 0.6905160053573874 0.053289131755115966 0.0522301568160505

UP_SEQ_FEATURE REPEAT:8 7 2.8925619834710745 0.001048135892261748 4582, 94025, 4584, 7428, 2130, 6490, 5903 188 126 20543 6.070626477541371 0.7718148616615673 0.06420971618246969 0.06293372466145539

UP_SEQ_FEATURE REPEAT:7 7 2.8925619834710745 0.00161126559287939 4582, 94025, 4584, 7428, 2130, 6490, 5903 188 137 20543 5.583203913651188 0.8969049802309106 0.09459471751529419 0.0927149076569349

UP_SEQ_FEATURE REPEAT:11 6 2.479338842975207 0.0016904132802286896 4582, 94025, 4584, 2130, 6490, 5903 188 94 20543 6.974762335898596 0.9078011563314882 0.09527169247368894 0.09337842959983282

UP_SEQ_FEATURE REPEAT:4 8 3.3057851239669422 0.001869990111467708 4582, 94025, 4583, 4584, 7428, 2130, 6490, 5903 188 191 20543 4.5768073966804055 0.9284445555236399 0.09758578026140743 0.09564653125692239

UP_SEQ_FEATURE REPEAT:10 6 2.479338842975207 0.0023180487810458097 4582, 94025, 4584, 2130, 6490, 5903 188 101 20543 6.491362966083843 0.9619906127886647 0.11170524082225311 0.10948540637014303

UP_SEQ_FEATURE REPEAT:16 5 2.066115702479339 0.0024576738576932906 4582, 4583, 4584, 2130, 5903 188 62 20543 8.81219972546328 0.9687933711541441 0.11170524082225311 0.10948540637014303

UP_SEQ_FEATURE REPEAT:9 6 2.479338842975207 0.003487733170179141 4582, 94025, 4584, 2130, 6490, 5903 188 111 20543 5.906555491661875 0.9927213200019287 0.14453576578771793 0.14166351494168805

UP_SEQ_FEATURE REPEAT:15 5 2.066115702479339 0.004224801045316619 4582, 4583, 4584, 2130, 5903 188 72 20543 7.588283096926714 0.9974338659752385 0.1700784192243176 0.1666985783880643

UP_SEQ_FEATURE REPEAT:3 8 3.3057851239669422 0.004565275085481731 4582, 94025, 4583, 4584, 7428, 2130, 6490, 5903 188 224 20543 3.902545592705167 0.9984150468658028 0.1774302688138752 0.17390433018592025

UP_SEQ_FEATURE REPEAT:14 5 2.066115702479339 0.006414538311155943 4582, 4583, 4584, 2130, 5903 188 81 20543 6.745140530601524 0.9998846104324732 0.23174575590817242 0.22714044635144506

SMART SM00200:SEA 3 1.2396694214876034 0.006922323297982961 4582, 94025, 4584 122 11 10378 23.199701937406857 0.527732880454826 0.1716931148211089 0.16533411056847525

UP_SEQ_FEATURE REPEAT:22 4 1.6528925619834711 0.007955824446064555 4582, 4584, 2130, 5903 188 45 20543 9.713002364066194 0.9999870522172468 0.2734086986464624 0.26797545268329637

UP_SEQ_FEATURE REPEAT:13 5 2.066115702479339 0.008229248639133595 4582, 4583, 4584, 2130, 5903 188 87 20543 6.279958425042798 0.9999912195580397 0.27607169839379136 0.2705855326343689

UP_SEQ_FEATURE REPEAT:21 4 1.6528925619834711 0.008971626811776193 4582, 4584, 2130, 5903 188 47 20543 9.299683114531462 0.9999969429767273 0.2912349155684589 0.2854474225692276

UP_SEQ_FEATURE REPEAT:31 3 1.2396694214876034 0.009094631855934841 4582, 4584, 2130 188 16 20543 20.488364361702125 0.9999974334881794 0.2912349155684589 0.2854474225692276

UP_SEQ_FEATURE REPEAT:20 4 1.6528925619834711 0.00950665226891294 4582, 4584, 2130, 5903 188 48 20543 9.105939716312056 0.9999985715915038 0.2976638454866296 0.2917485951859727

UP_SEQ_FEATURE REPEAT:19 4 1.6528925619834711 0.01063156756122273 4582, 4584, 2130, 5903 188 50 20543 8.741702127659574 0.9999997119343472 0.3187208232715495 0.31238712344784236

UP_SEQ_FEATURE REPEAT:18 4 1.6528925619834711 0.011830362726230356 4582, 4584, 2130, 5903 188 52 20543 8.405482815057283 0.9999999478095205 0.3401832873726239 0.33342308010049226

UP_SEQ_FEATURE REPEAT:17 4 1.6528925619834711 0.015877085645735325 4582, 4584, 2130, 5903 188 58 20543 7.535950110051357 0.9999999998391113 0.40674206681529224 0.39865918685019064

UP_SEQ_FEATURE REPEAT:30 3 1.2396694214876034 0.016893193033670817 4582, 4584, 2130 188 22 20543 14.900628626692454 0.9999999999624689 0.4250448032936104 0.41659820677677495

UP_SEQ_FEATURE DOMAIN:SEA 3 1.2396694214876034 0.0183925958212933 4582, 94025, 4584 188 23 20543 14.25277520814061 0.9999999999956308 0.439240127325462 0.43051143778315326

UP_SEQ_FEATURE REPEAT:29 3 1.2396694214876034 0.021552371354139035 4582, 4584, 2130 188 25 20543 13.112553191489361 0.9999999999999535 0.5061215206330317 0.4960637473344334

INTERPRO IPR000082:SEA domain 3 1.2396694214876034 0.021627706194134344 4582, 94025, 4584 183 24 19144 13.076502732240439 0.999909891448139 0.30711342795670765 0.29774142193924946

UP_SEQ_FEATURE REPEAT:28 3 1.2396694214876034 0.024919538690442042 4582, 4584, 2130 188 27 20543 12.141252955082741 0.9999999999999997 0.5526148204229426 0.5416331206558437

UP_SEQ_FEATURE REPEAT:1 7 2.8925619834710745 0.026760709420363904 94025, 4583, 4584, 7428, 2130, 6490, 5903 188 249 20543 3.0718832777920193 1.0 0.5526148204229426 0.5416331206558437

UP_SEQ_FEATURE REPEAT:2 7 2.8925619834710745 0.028108439231552745 94025, 4583, 4584, 7428, 2130, 6490, 5903 188 252 20543 3.0353132387706854 1.0 0.557447898552168 0.5463701546490732

UP_SEQ_FEATURE REPEAT:27 3 1.2396694214876034 0.03224240221596307 4582, 4584, 2130 188 31 20543 10.574639670555936 1.0 0.6057272629638928 0.5936900994699333

UP_SEQ_FEATURE REPEAT:26 3 1.2396694214876034 0.036181859549850794 4582, 4584, 2130 188 33 20543 9.93375241779497 1.0 0.6707926329702601 0.6574624741887362

UP_SEQ_FEATURE REPEAT:24 3 1.2396694214876034 0.04457792250917552 4582, 4584, 2130 188 37 20543 8.859833237492811 1.0 0.7587795707825464 0.7437009135916938

UP_SEQ_FEATURE REPEAT:25 3 1.2396694214876034 0.04457792250917552 4582, 4584, 2130 188 37 20543 8.859833237492811 1.0 0.7587795707825464 0.7437009135916938

UP_SEQ_FEATURE REPEAT:23 3 1.2396694214876034 0.05129823176720391 4582, 4584, 2130 188 40 20543 8.19534574468085 1.0 0.8003651833327734 0.7844601264602982

Annotation Cluster 27 Enrichment Score: 2.180282870147683

Category Term Count % PValue Genes List Total Pop Hits Pop Total Fold Enrichment Bonferroni Benjamini FDR

UP_KW_MOLECULAR_FUNCTION KW-0497~Mitogen 5 2.066115702479339 0.0010256709294917473 4904, 2247, 7422, 2250, 7042 130 41 11749 11.02157598499062 0.06260490799606422 0.012923453711596014 0.012308051153900967

GOTERM_BP_DIRECT GO:0051781~positive regulation of cell division 5 2.066115702479339 0.0011391816903635376 4904, 2247, 7422, 2250, 7042 183 49 19414 10.8252481320397 0.9060647375103612 0.028140500089337387 0.02662159116885267

GOTERM_MF_DIRECT GO:0008083~growth factor activity 8 3.3057851239669422 0.0012491704596836288 1437, 2247, 7422, 1442, 3593, 3592, 2250, 7042 185 167 18945 4.905648163133193 0.43516846182681757 0.038058060005027886 0.03697544560663541

UP_KW_MOLECULAR_FUNCTION KW-0339~Growth factor 6 2.479338842975207 0.015538891818239413 1437, 2247, 7422, 3592, 2250, 7042 130 133 11749 4.077154424522846 0.6271711479696139 0.13985002636415472 0.13319050129919496

GOTERM_BP_DIRECT GO:0007399~nervous system development 5 2.066115702479339 0.5532773073414055 5077, 2247, 7422, 2250, 10643 183 418 19414 1.2689884173922137 1.0 1.0 0.946480231436837

Annotation Cluster 28 Enrichment Score: 2.159089331434053

Category Term Count % PValue Genes List Total Pop Hits Pop Total Fold Enrichment Bonferroni Benjamini FDR

GOTERM_CC_DIRECT GO:0005796~Golgi lumen 8 3.3057851239669422 5.469983494609397E-5 4582, 94025, 4583, 4584, 4585, 2719, 727897, 6382 188 107 20624 8.202028236229866 0.017996927588707745 0.001849740201265746 0.001710452535507783

INTERPRO IPR001846:von Willebrand factor, type D domain 3 1.2396694214876034 0.012443675833671482 4583, 4585, 727897 183 18 19144 17.435336976320585 0.9951765415885473 0.21304909108966855 0.20654759300477255

SMART SM00216:VWD 3 1.2396694214876034 0.016354458898227932 4583, 4585, 727897 122 17 10378 15.011571841851493 0.8315109105926224 0.24650998117693093 0.23737998187408166

GOTERM_CC_DIRECT GO:0031012~extracellular matrix 5 2.066115702479339 0.20752565333913758 1116, 4583, 7422, 4585, 727897 188 258 20624 2.1260102259607456 1.0 0.8721331254252365 0.8064604503179144

Annotation Cluster 29 Enrichment Score: 2.141427936079013

Category Term Count % PValue Genes List Total Pop Hits Pop Total Fold Enrichment Bonferroni Benjamini FDR

BBID 58.(CD40L)_immnosurveillance 7 2.8925619834710745 3.200729188365703E-4 3458, 7124, 3383, 3592, 925, 958, 959 28 16 388 6.0625 0.021533219770591416 0.02144488556205021 0.02144488556205021

KEGG_PATHWAY hsa04064:NF-kappa B signaling pathway 7 2.8925619834710745 0.004638990287774607 7099, 598, 6366, 7124, 3383, 958, 959 128 104 8465 4.451246995192308 0.6828868401957457 0.015277741347737706 0.00977280620624517

BBID 55.Allergen_recognition_by_Th2_or_Th0_cell 3 1.2396694214876034 0.25353843085716476 3383, 958, 959 28 14 388 2.969387755102041 0.9999999976852354 0.9812803218491704 0.9812803218491704

Annotation Cluster 30 Enrichment Score: 1.952059152467801

Category Term Count % PValue Genes List Total Pop Hits Pop Total Fold Enrichment Bonferroni Benjamini FDR

GOTERM_BP_DIRECT GO:0001916~positive regulation of T cell mediated cytotoxicity 7 2.8925619834710745 9.637569569312762E-7 6375, 3106, 3105, 3123, 5788, 3593, 3592 183 36 19414 20.62811171827565 0.001997798388181593 1.3331971237549322E-4 1.2612366043040637E-4

KEGG_PATHWAY hsa05332:Graft-versus-host disease 7 2.8925619834710745 3.414030062867469E-5 3106, 3458, 3105, 7124, 941, 942, 3123 128 42 8465 11.022135416666668 0.008397341897737332 2.465164739131549E-4 1.576906999120586E-4

KEGG_PATHWAY hsa04612:Antigen processing and presentation 7 2.8925619834710745 0.0010766810385958161 3106, 3458, 3105, 7124, 3123, 925, 3308 128 78 8465 5.934995993589744 0.2336248565914778 0.004155315883330728 0.002658056314033421

GOTERM_BP_DIRECT GO:0060333~interferon-gamma-mediated signaling pathway 4 1.6528925619834711 0.002008585442961656 3106, 3458, 3105, 7157 183 27 19414 15.716656547257639 0.9845787077917495 0.04209913933480238 0.03982680024781546

GOTERM_CC_DIRECT GO:0071556~integral component of lumenal side of endoplasmic reticulum membrane 4 1.6528925619834711 0.002253280488368217 3106, 3105, 3123, 10134 188 29 20624 15.131327953044753 0.5271292660926847 0.037404456106912405 0.03458785549645213

GOTERM_CC_DIRECT GO:0098553~lumenal side of endoplasmic reticulum membrane 4 1.6528925619834711 0.002253280488368217 3106, 3105, 3123, 10134 188 29 20624 15.131327953044753 0.5271292660926847 0.037404456106912405 0.03458785549645213

GOTERM_BP_DIRECT GO:0016045~detection of bacterium 3 1.2396694214876034 0.00542308129540803 3106, 3105, 3123 183 12 19414 26.52185792349727 0.9999874214444247 0.08791323193727861 0.08316803580379659

INTERPRO IPR001039:MHC class I, alpha chain, alpha1/alpha2 3 1.2396694214876034 0.006544445384297538 3106, 3105, 3123 183 13 19144 24.14123581336696 0.9390131170971681 0.14673335440582902 0.14225557598499383

GOTERM_BP_DIRECT GO:0019882~antigen processing and presentation 4 1.6528925619834711 0.008615193722935857 3106, 3105, 3123, 925 183 45 19414 9.429993928354584 0.9999999840527112 0.12860810773447412 0.12166636890736035

INTERPRO IPR000353:MHC class II, beta chain, N-terminal 3 1.2396694214876034 0.012443675833671482 3106, 3105, 3123 183 18 19144 17.435336976320585 0.9951765415885473 0.21304909108966855 0.20654759300477255

GOTERM_BP_DIRECT GO:0002504~antigen processing and presentation of peptide or polysaccharide antigen via MHC class II 3 1.2396694214876034 0.014863777575106513 3106, 3105, 3123 183 20 19414 15.91311475409836 0.999999999999968 0.19061060884945824 0.18032222899830674

GOTERM_CC_DIRECT GO:0012507~ER to Golgi transport vesicle membrane 4 1.6528925619834711 0.014998437375035593 2348, 3106, 3105, 3123 188 57 20624 7.69839492347891 0.9933769617322504 0.15560878776599427 0.14389125856674773

UP_SEQ_FEATURE DOMAIN:MHC class I-like antigen recognition-like 3 1.2396694214876034 0.015448924615276888 3106, 3105, 3123 188 21 20543 15.610182370820667 0.9999999997030417 0.40674206681529224 0.39865918685019064

UP_KW_CELLULAR_COMPONENT KW-0491~MHC II 3 1.2396694214876034 0.017494115211944234 3106, 3105, 3123 182 20 17708 14.594505494505494 0.46082392829330876 0.12245880648360964 0.1119623373564431

SMART SM00921:SM00921 3 1.2396694214876034 0.018259998605698588 3106, 3105, 3123 122 18 10378 14.1775956284153 0.8633465561849382 0.24650998117693093 0.23737998187408166

INTERPRO IPR014745:MHC class II, alpha/beta chain, N-terminal 3 1.2396694214876034 0.021627706194134344 3106, 3105, 3123 183 24 19144 13.076502732240439 0.999909891448139 0.30711342795670765 0.29774142193924946

GOTERM_CC_DIRECT GO:0042613~MHC class II protein complex 3 1.2396694214876034 0.02304149796691505 3106, 3105, 3123 188 26 20624 12.657937806873976 0.9995646177193155 0.19614813653886656 0.18137794553443384

INTERPRO IPR011161:MHC class I-like antigen recognition 3 1.2396694214876034 0.030847670387453705 3106, 3105, 3123 183 29 19144 10.82193329564726 0.9999984040813121 0.3754602167158651 0.36400251057195376

UP_SEQ_FEATURE DOMAIN:Ig-like C1-type 3 1.2396694214876034 0.05129823176720391 3106, 3105, 3123 188 40 20543 8.19534574468085 1.0 0.8003651833327734 0.7844601264602982

INTERPRO IPR011162:MHC classes I/II-like antigen recognition protein 3 1.2396694214876034 0.07357118424817777 3106, 3105, 3123 183 47 19144 6.677363097314267 0.9999999999999927 0.6814615521716899 0.6606657771054177

GOTERM_MF_DIRECT GO:0042605~peptide antigen binding 3 1.2396694214876034 0.07633388979726999 3106, 3105, 3123 185 47 18945 6.536515238642899 0.9999999999999998 0.742225268879838 0.7211116397869761

INTERPRO IPR003006:Immunoglobulin/major histocompatibility complex, conserved site 3 1.2396694214876034 0.13987253141990547 3106, 3105, 3123 183 69 19144 4.548348776431457 1.0 1.0 0.971764705882353

INTERPRO IPR003597:Immunoglobulin C1-set 3 1.2396694214876034 0.1663586375279613 3106, 3105, 3123 183 77 19144 4.075793059399617 1.0 1.0 0.971764705882353

KEGG_PATHWAY hsa04145:Phagosome 5 2.066115702479339 0.19422052083450156 7099, 3106, 4153, 3105, 3123 128 152 8465 2.1754214638157894 1.0 0.36620205073375484 0.23425070451794844

SMART SM00407:IGc1 3 1.2396694214876034 0.21343722143493493 3106, 3105, 3123 122 74 10378 3.448604342046965 0.9999999999945146 1.0 0.9719626168224299

GOTERM_CC_DIRECT GO:0005765~lysosomal membrane 6 2.479338842975207 0.2730274120153748 2548, 55, 3106, 3105, 3123, 4758 188 386 20624 1.7052144195788776 1.0 0.9961000086714772 0.9210924778980226

Annotation Cluster 31 Enrichment Score: 1.8652055617760601

Category Term Count % PValue Genes List Total Pop Hits Pop Total Fold Enrichment Bonferroni Benjamini FDR

INTERPRO IPR021184:Tumour necrosis factor, conserved site 3 1.2396694214876034 0.005572276295565545 8743, 7124, 959 183 12 19144 26.153005464480877 0.907489033064279 0.13963468834770132 0.13537353588638648

UP_SEQ_FEATURE DOMAIN:TNF family profile 3 1.2396694214876034 0.010245990567364352 8743, 7124, 959 188 17 20543 19.28316645807259 0.9999995012039027 0.3138391458568776 0.30760245594630803

INTERPRO IPR006052:Tumour necrosis factor 3 1.2396694214876034 0.012443675833671482 8743, 7124, 959 183 18 19144 17.435336976320585 0.9951765415885473 0.21304909108966855 0.20654759300477255

SMART SM00207:TNF 3 1.2396694214876034 0.014540171991447328 8743, 7124, 959 122 16 10378 15.949795081967213 0.7944090898688845 0.24650998117693093 0.23737998187408166

INTERPRO IPR008983:Tumour necrosis factor-like domain 4 1.6528925619834711 0.016205090901910073 8743, 7124, 8995, 959 183 56 19144 7.472287275565964 0.9990508230433363 0.2465488830076318 0.23902509080317358

GOTERM_MF_DIRECT GO:0005164~tumor necrosis factor receptor binding 3 1.2396694214876034 0.03846009309571671 8743, 7124, 959 185 32 18945 9.600506756756758 0.9999999835539245 0.5384487728111228 0.523131849295708

Annotation Cluster 32 Enrichment Score: 1.7847489850482963

Category Term Count % PValue Genes List Total Pop Hits Pop Total Fold Enrichment Bonferroni Benjamini FDR

GOTERM_BP_DIRECT GO:0070372~regulation of ERK1 and ERK2 cascade 4 1.6528925619834711 0.0022342184416190027 634, 2064, 1969, 1956 183 28 19414 15.155347384855581 0.9903537873792221 0.045901022439202285 0.04342347327621884

GOTERM_CC_DIRECT GO:0032587~ruffle membrane 5 2.066115702479339 0.013513097337692149 4771, 2064, 1969, 6868, 1956 188 101 20624 5.430798398988835 0.9890775657782844 0.1447209134230256 0.13382325427972547

INTERPRO IPR009030:Insulin-like growth factor binding protein, N-terminal 4 1.6528925619834711 0.14651176745691724 3484, 2064, 1969, 1956 183 139 19144 3.010417895192043 1.0 1.0 0.971764705882353

Annotation Cluster 33 Enrichment Score: 1.6232845133912217

Category Term Count % PValue Genes List Total Pop Hits Pop Total Fold Enrichment Bonferroni Benjamini FDR

UP_SEQ_FEATURE DOMAIN:RanBP2-type 4 1.6528925619834711 4.5944152168909605E-4 2521, 2130, 4193, 5903 188 17 20543 25.710888610763455 0.47664844951636054 0.035964061336663126 0.03524937452514676

INTERPRO IPR001876:Zinc finger, RanBP2-type 4 1.6528925619834711 0.001666401384647102 2521, 2130, 4193, 5903 183 25 19144 16.737923497267758 0.5085913056870219 0.05070621356140467 0.04915884084708951

UP_SEQ_FEATURE ZN_FING:RanBP2-type 3 1.2396694214876034 0.012730318098716609 2521, 2130, 4193 188 19 20543 17.253359462486 0.9999999855441764 0.358740364021834 0.3516113858865527

SMART SM00547:ZnF_RBZ 3 1.2396694214876034 0.02233552689024142 2521, 2130, 5903 122 20 10378 12.75983606557377 0.9128028971486479 0.26802632268289706 0.2580994218427897

UP_KW_MOLECULAR_FUNCTION KW-0694~RNA-binding 7 2.8925619834710745 0.8387296022903625 4904, 2521, 4343, 2130, 7490, 10643, 5903 130 749 11749 0.8446441409058232 1.0 1.0 0.967741935483871

UP_KW_DOMAIN KW-0863~Zinc-finger 10 4.132231404958678 0.9972995687584302 9014, 367, 2521, 80312, 54894, 2130, 9978, 7490, 4193, 5903 149 1840 14504 0.529034140647797 1.0 1.0 0.9972995687584302

Annotation Cluster 34 Enrichment Score: 1.5817396152192778

Category Term Count % PValue Genes List Total Pop Hits Pop Total Fold Enrichment Bonferroni Benjamini FDR

GOTERM_BP_DIRECT GO:0045429~positive regulation of nitric oxide biosynthetic process 5 2.066115702479339 8.967483449040548E-4 7099, 3458, 7124, 207, 3043 183 46 19414 11.531242575433595 0.8445743238171307 0.02325941019594892 0.022003962513083244

GOTERM_BP_DIRECT GO:0048661~positive regulation of smooth muscle cell proliferation 4 1.6528925619834711 0.01715038637998365 7099, 2247, 7124, 207 183 58 19414 7.316374599585453 0.9999999999999998 0.2081114136752402 0.19687841206963685

GOTERM_BP_DIRECT GO:0031663~lipopolysaccharide-mediated signaling pathway 3 1.2396694214876034 0.058940003320477226 7099, 7124, 207 183 42 19414 7.577673692427792 1.0 0.44472911596360093 0.4207244600658065

KEGG_PATHWAY hsa04217:Necroptosis 6 2.479338842975207 0.0906210776938031 7099, 3458, 8743, 3456, 7124, 3440 128 159 8465 2.495577830188679 0.9999999999354366 0.19634566833657338 0.12559763399667448

KEGG_PATHWAY hsa05135:Yersinia infection 5 2.066115702479339 0.15021664319238165 7099, 3456, 7124, 207, 925 128 137 8465 2.413606295620438 1.0 0.2968280869481461 0.1898738369951704

Annotation Cluster 35 Enrichment Score: 1.5578790746507478

Category Term Count % PValue Genes List Total Pop Hits Pop Total Fold Enrichment Bonferroni Benjamini FDR

GOTERM_CC_DIRECT GO:0043202~lysosomal lumen 7 2.8925619834710745 2.704788096872941E-4 2548, 2760, 2719, 2581, 2717, 4758, 6382 188 98 20624 7.835866261398175 0.0858961672096914 0.006414211772584403 0.005931213898142806

UP_KW_CELLULAR_COMPONENT KW-0458~Lysosome 11 4.545454545454546 0.007053775306120711 2548, 55, 3106, 51284, 3105, 3123, 2760, 2581, 54106, 2717, 4758 182 391 17708 2.737247407324134 0.219450388774182 0.061720533928556226 0.05643020244896569

INTERPRO IPR017853:Glycoside hydrolase, superfamily 4 1.6528925619834711 0.0154427350454013 2548, 1116, 2581, 2717 183 55 19144 7.608147044212617 0.9986797445534445 0.24525886679269482 0.2377744412802417

INTERPRO IPR013780:Glycosyl hydrolase, family 13, all-beta 3 1.2396694214876034 0.02336347182964464 2548, 2581, 2717 183 25 19144 12.55344262295082 0.9999577095260037 0.3110262187321443 0.30153480830135115

GOTERM_CC_DIRECT GO:0005764~lysosome 8 3.3057851239669422 0.023974977551408372 2548, 55, 7299, 51284, 2581, 54106, 2717, 4758 188 312 20624 2.812875068194217 0.9996830169664583 0.19899231367668951 0.18400795270705925

KEGG_PATHWAY hsa00600:Sphingolipid metabolism 4 1.6528925619834711 0.04488592923865979 2583, 2581, 2717, 4758 128 53 8465 4.991155660377358 0.9999881519996723 0.10869435805832321 0.0695291845069436

UP_SEQ_FEATURE ACT_SITE:Nucleophile 8 3.3057851239669422 0.0575680532488334 2548, 55, 216, 5698, 217, 2581, 2717, 4758 188 377 20543 2.318753880015802 1.0 0.8538251266063817 0.8368577003856729

UP_KW_MOLECULAR_FUNCTION KW-0326~Glycosidase 4 1.6528925619834711 0.06307842369207993 2548, 2581, 2717, 4758 130 83 11749 4.35551436515292 0.9835068296152831 0.49674258657512943 0.47308817769059947

KEGG_PATHWAY hsa04142:Lysosome 5 2.066115702479339 0.13650918656967198 2548, 2760, 2581, 2717, 4758 128 132 8465 2.5050307765151514 0.9999999999999998 0.2763751564156474 0.1767905858853129

UP_KW_BIOLOGICAL_PROCESS KW-0443~Lipid metabolism 8 3.3057851239669422 0.7182904658181057 55, 6489, 216, 2760, 2583, 2581, 2717, 4758 125 743 11262 0.970078061911171 1.0 1.0 0.926470588235294

Annotation Cluster 36 Enrichment Score: 1.4968379609284668

Category Term Count % PValue Genes List Total Pop Hits Pop Total Fold Enrichment Bonferroni Benjamini FDR

UP_SEQ_FEATURE DOMAIN:F5/8 type C 3 1.2396694214876034 0.02321056606283909 4240, 8829, 780 188 26 20543 12.608224222585923 0.9999999999999958 0.5361260259432832 0.5254719956193571

INTERPRO IPR000421:Coagulation factor 5/8 C-terminal type domain 3 1.2396694214876034 0.02889780754284461 4240, 8829, 780 183 28 19144 11.208430913348947 0.9999962428186983 0.36207252980152366 0.3510233680939654

SMART SM00231:FA58C 3 1.2396694214876034 0.0314814908683533 4240, 8829, 780 122 24 10378 10.633196721311474 0.9684022701140432 0.2833334178151797 0.27283958752572857

INTERPRO IPR008979:Galactose-binding domain-like 4 1.6528925619834711 0.048757590847920855 4240, 1969, 8829, 780 183 86 19144 4.865675435252256 0.9999999994349941 0.5325829154157509 0.5163303851331106

Annotation Cluster 37 Enrichment Score: 1.4657741555650807

Category Term Count % PValue Genes List Total Pop Hits Pop Total Fold Enrichment Bonferroni Benjamini FDR

GOTERM_CC_DIRECT GO:0000307~cyclin-dependent protein kinase holoenzyme complex 4 1.6528925619834711 0.006936388203301551 891, 983, 900, 10983 188 43 20624 10.204849084611578 0.9008285241434667 0.08529188457393018 0.07886930290420652

UP_SEQ_FEATURE DOMAIN:Cyclin-like 3 1.2396694214876034 0.011458283477361934 891, 900, 10983 188 18 20543 18.211879432624112 0.9999999112932192 0.33634836290839515 0.3296643642132673

GOTERM_BP_DIRECT GO:0044772~mitotic cell cycle phase transition 3 1.2396694214876034 0.021070284435814376 891, 900, 10983 183 24 19414 13.260928961748634 1.0 0.24022439672700457 0.2272580678434265

UP_SEQ_FEATURE DOMAIN:Cyclin N-terminal 3 1.2396694214876034 0.02848562717938687 891, 900, 10983 188 29 20543 11.303925165077036 1.0 0.557447898552168 0.5463701546490732

GOTERM_MF_DIRECT GO:0016538~cyclin-dependent protein serine/threonine kinase regulator activity 3 1.2396694214876034 0.03628346520906469 891, 900, 10983 185 31 18945 9.910200523103748 0.9999999537794785 0.5348885032433085 0.519672856542733

INTERPRO IPR006671:Cyclin, N-terminal 3 1.2396694214876034 0.04357101072709254 891, 900, 10983 183 35 19144 8.966744730679157 0.999999994271203 0.48845396236161637 0.4735480902707689

INTERPRO IPR013763:Cyclin-like 3 1.2396694214876034 0.05539721798566766 891, 900, 10983 183 40 19144 7.845901639344262 0.9999999999714131 0.5788386233411716 0.5611745338964879

SMART SM00385:CYCLIN 3 1.2396694214876034 0.0722200342734355 891, 900, 10983 122 38 10378 6.715703192407247 0.9996951691308292 0.599981823194695 0.577760274187484

UP_KW_MOLECULAR_FUNCTION KW-0195~Cyclin 3 1.2396694214876034 0.07749977948750908 891, 900, 10983 130 42 11749 6.455494505494506 0.9937929083983498 0.5424984564125636 0.5166651965833939

GOTERM_BP_DIRECT GO:0000079~regulation of cyclin-dependent protein serine/threonine kinase activity 3 1.2396694214876034 0.09404956750201635 891, 900, 10983 183 55 19414 5.7865871833084945 1.0 0.5656604422222722 0.5351284087143713

Annotation Cluster 38 Enrichment Score: 1.4260004788788032

Category Term Count % PValue Genes List Total Pop Hits Pop Total Fold Enrichment Bonferroni Benjamini FDR

INTERPRO IPR008983:Tumour necrosis factor-like domain 4 1.6528925619834711 0.016205090901910073 8743, 7124, 8995, 959 183 56 19144 7.472287275565964 0.9990508230433363 0.2465488830076318 0.23902509080317358

UP_SEQ_FEATURE TRANSMEM:Helical; Signal-anchor for type II membrane protein 9 3.71900826446281 0.031124421289114433 8743, 2346, 7124, 952, 6489, 8995, 2583, 959, 170482 188 402 20543 2.446371864083836 1.0 0.6007439670734552 0.5888058328803703

UP_KW_DOMAIN KW-0735~Signal-anchor 9 3.71900826446281 0.10453116886402863 8743, 2346, 7124, 952, 6489, 8995, 2583, 959, 170482 149 466 14504 1.880001152173287 0.8772676633475356 0.3972184416833088 0.35540597413769737

Annotation Cluster 39 Enrichment Score: 1.4042395760419732

Category Term Count % PValue Genes List Total Pop Hits Pop Total Fold Enrichment Bonferroni Benjamini FDR

GOTERM_BP_DIRECT GO:0032722~positive regulation of chemokine production 7 2.8925619834710745 3.7579124677712837E-6 7099, 1436, 3458, 51284, 7124, 54106, 6868 183 45 19414 16.502489374620524 0.0077673599595941 3.249028487760589E-4 3.0736592392645957E-4

GOTERM_BP_DIRECT GO:0032757~positive regulation of interleukin-8 production 7 2.8925619834710745 3.263322989508274E-5 7099, 1116, 51284, 7124, 965, 54106, 11027 183 65 19414 11.424800336275746 0.06547327743376197 0.0018301068116836942 0.0017313251428120924

GOTERM_BP_DIRECT GO:1901224~positive regulation of NIK/NF-kappaB signaling 7 2.8925619834710745 5.401469985570723E-5 7099, 51284, 7124, 942, 3593, 54106, 1956 183 71 19414 10.45932425152005 0.10603041827791615 0.0026685833857283928 0.0025245441861131736

GOTERM_BP_DIRECT GO:0032755~positive regulation of interleukin-6 production 7 2.8925619834710745 4.2120053251854737E-4 3329, 7099, 3458, 51284, 7124, 54106, 11027 183 103 19414 7.209825454931297 0.5827940188032034 0.01344601699963055 0.01272025608206013

GOTERM_BP_DIRECT GO:0032727~positive regulation of interferon-alpha production 4 1.6528925619834711 0.0017977190724351795 3329, 7099, 51284, 54106 183 26 19414 16.32114333753678 0.9760939588773558 0.03845636160106183 0.03638064473392018

GOTERM_MF_DIRECT GO:0003953~NAD+ nucleosidase activity 4 1.6528925619834711 0.002220595622439623 7099, 51284, 952, 54106 185 27 18945 15.17117117117117 0.6379382907090919 0.06342576246593173 0.06162152852269954

UP_KW_BIOLOGICAL_PROCESS KW-0395~Inflammatory response 8 3.3057851239669422 0.004851915749409366 7099, 1436, 1116, 51284, 9447, 6366, 54106, 6280 125 189 11262 3.813587301587302 0.28508883648085614 0.04782602667274947 0.04366724174468429

GOTERM_BP_DIRECT GO:0007252~I-kappaB phosphorylation 3 1.2396694214876034 0.00542308129540803 7099, 51284, 54106 183 12 19414 26.52185792349727 0.9999874214444247 0.08791323193727861 0.08316803580379659

GOTERM_MF_DIRECT GO:0004888~transmembrane signaling receptor activity 7 2.8925619834710745 0.0102543079527542 7099, 51284, 2022, 2064, 54106, 3383, 1956 185 188 18945 3.812967222541691 0.9909989246583015 0.23431093672043346 0.22764563655114323

GOTERM_BP_DIRECT GO:0007249~I-kappaB kinase/NF-kappaB signaling 4 1.6528925619834711 0.022236999845052983 51284, 7124, 207, 54106 183 64 19414 6.630464480874317 1.0 0.24859857635737903 0.2351802435612217

UP_SEQ_FEATURE DOMAIN:TIR 3 1.2396694214876034 0.024919538690442042 7099, 51284, 54106 188 27 20543 12.141252955082741 0.9999999999999997 0.5526148204229426 0.5416331206558437

GOTERM_BP_DIRECT GO:0002224~toll-like receptor signaling pathway 3 1.2396694214876034 0.026310512917143095 7099, 51284, 54106 183 27 19414 11.78749241044323 1.0 0.2771284990003651 0.262170237849502

UP_SEQ_FEATURE REPEAT:LRR 18 3 1.2396694214876034 0.026678237918023418 7099, 51284, 54106 188 28 20543 11.7076367781155 1.0 0.5526148204229426 0.5416331206558437

SMART SM00255:TIR 3 1.2396694214876034 0.02674854717973162 7099, 51284, 54106 122 22 10378 11.599850968703429 0.9465058176472182 0.2833334178151797 0.27283958752572857

INTERPRO IPR000157:Toll/interleukin-1 receptor homology (TIR) domain 3 1.2396694214876034 0.026999709797619034 7099, 51284, 54106 183 27 19144 11.623557984213722 0.9999913677276424 0.3485417082965366 0.33790545898232305

GOTERM_BP_DIRECT GO:0001774~microglial cell activation 3 1.2396694214876034 0.030065318634452186 3458, 7124, 54106 183 29 19414 10.97456189937818 1.0 0.29707398174518235 0.28103914514014117

UP_SEQ_FEATURE REPEAT:LRR 17 3 1.2396694214876034 0.040296219694738615 7099, 51284, 54106 188 35 20543 9.366109422492402 1.0 0.7373684876608664 0.7227153168627797

UP_SEQ_FEATURE REPEAT:LRR 16 3 1.2396694214876034 0.04901962184196782 7099, 51284, 54106 188 39 20543 8.405482815057283 1.0 0.8003651833327734 0.7844601264602982

GOTERM_BP_DIRECT GO:0032728~positive regulation of interferon-beta production 3 1.2396694214876034 0.061444615600457955 7099, 51284, 54106 183 43 19414 7.401448722836448 1.0 0.454688169101384 0.4301459643113334

UP_SEQ_FEATURE REPEAT:LRR 15 3 1.2396694214876034 0.06824666339280616 7099, 51284, 54106 188 47 20543 6.974762335898596 1.0 0.9913355538192152 0.9716354860357248

UP_SEQ_FEATURE REPEAT:LRR 14 3 1.2396694214876034 0.07080057691831923 7099, 51284, 54106 188 48 20543 6.829454787234042 1.0 0.997580128779118 0.9777559672419887

UP_SEQ_FEATURE REPEAT:LRR 13 3 1.2396694214876034 0.09508207437189917 7099, 51284, 54106 188 57 20543 5.751119820828667 1.0 1.0 0.9808238636363636

GOTERM_BP_DIRECT GO:0007254~JNK cascade 3 1.2396694214876034 0.10881998461777416 7099, 51284, 7124 183 60 19414 5.304371584699454 1.0 0.6102742380591388 0.5773341346072721

UP_SEQ_FEATURE REPEAT:LRR 7 5 2.066115702479339 0.11768861502609386 7099, 51284, 54106, 23532, 7162 188 205 20543 2.6651530877010896 1.0 1.0 0.9808238636363636

GOTERM_BP_DIRECT GO:0032715~negative regulation of interleukin-6 production 3 1.2396694214876034 0.14640932291489545 7099, 7124, 54106 183 72 19414 4.4203096539162114 1.0 0.7278039905025133 0.6885201124609319

UP_SEQ_FEATURE REPEAT:LRR 12 3 1.2396694214876034 0.1491070504926825 7099, 51284, 54106 188 75 20543 4.370851063829787 1.0 1.0 0.9808238636363636

UP_SEQ_FEATURE REPEAT:LRR 6 5 2.066115702479339 0.15839886884569276 7099, 51284, 54106, 23532, 7162 188 230 20543 2.3754625346901017 1.0 1.0 0.9808238636363636

UP_SEQ_FEATURE REPEAT:LRR 9 4 1.6528925619834711 0.1746533017258315 7099, 51284, 54106, 23532 188 158 20543 2.766361432803663 1.0 1.0 0.9808238636363636

INTERPRO IPR000483:Cysteine-rich flanking region, C-terminal 3 1.2396694214876034 0.1867466751413131 7099, 51284, 7162 183 83 19144 3.7811574165514514 1.0 1.0 0.971764705882353

UP_SEQ_FEATURE REPEAT:LRR 11 3 1.2396694214876034 0.20431879708274553 7099, 51284, 54106 188 92 20543 3.5631938020351526 1.0 1.0 0.9808238636363636

UP_SEQ_FEATURE REPEAT:LRR 8 4 1.6528925619834711 0.21419201962454287 7099, 51284, 54106, 23532 188 175 20543 2.497629179331307 1.0 1.0 0.9808238636363636

UP_SEQ_FEATURE REPEAT:LRR 5 5 2.066115702479339 0.21677825609898907 7099, 51284, 54106, 23532, 7162 188 262 20543 2.0853297060256617 1.0 1.0 0.9808238636363636

INTERPRO IPR003591:Leucine-rich repeat, typical subtype 4 1.6528925619834711 0.2483265653019256 7099, 51284, 54106, 7162 183 181 19144 2.31186788636295 1.0 1.0 0.971764705882353

SMART SM00082:LRRCT 3 1.2396694214876034 0.2522266043168082 7099, 51284, 7162 122 83 10378 3.0746592929093426 0.9999999999999767 1.0 0.9719626168224299

UP_SEQ_FEATURE REPEAT:LRR 10 3 1.2396694214876034 0.2579701361739114 7099, 51284, 54106 188 108 20543 3.0353132387706854 1.0 1.0 0.9808238636363636

SMART SM00369:LRR_TYP 4 1.6528925619834711 0.35355948849376206 7099, 51284, 54106, 7162 122 181 10378 1.8799021827732996 1.0 1.0 0.9719626168224299

BIOCARTA h_tollPathway:Toll-Like Receptor Pathway 3 1.2396694214876034 0.4056119751079605 7099, 51284, 54106 60 38 1623 2.1355263157894737 1.0 1.0 0.9550561797752809

UP_KW_DOMAIN KW-0433~Leucine-rich repeat 5 2.066115702479339 0.41061509384181105 7099, 51284, 54106, 23532, 7162 149 319 14504 1.5257410952851824 0.9999565908599504 1.0 0.9444444444444444

UP_SEQ_FEATURE REPEAT:LRR 4 4 1.6528925619834711 0.43214237551161166 7099, 51284, 54106, 7162 188 264 20543 1.6556254029658286 1.0 1.0 0.9808238636363636

UP_SEQ_FEATURE REPEAT:LRR 3 4 1.6528925619834711 0.4649644418897636 7099, 51284, 54106, 7162 188 277 20543 1.5779245717797066 1.0 1.0 0.9808238636363636

INTERPRO IPR001611:Leucine-rich repeat 4 1.6528925619834711 0.47005185553514667 7099, 51284, 54106, 7162 183 268 19144 1.5613734605660223 1.0 1.0 0.971764705882353

UP_SEQ_FEATURE REPEAT:LRR 2 4 1.6528925619834711 0.483936813907178 7099, 51284, 54106, 7162 188 286 20543 1.5282696027376879 1.0 1.0 0.9808238636363636

UP_SEQ_FEATURE REPEAT:LRR 1 4 1.6528925619834711 0.4916760706628083 7099, 51284, 54106, 7162 188 288 20543 1.5176566193853427 1.0 1.0 0.9808238636363636

Annotation Cluster 40 Enrichment Score: 1.400296846973307

Category Term Count % PValue Genes List Total Pop Hits Pop Total Fold Enrichment Bonferroni Benjamini FDR

GOTERM_BP_DIRECT GO:2001238~positive regulation of extrinsic apoptotic signaling pathway 4 1.6528925619834711 0.0027311552555872843 7099, 8743, 7124, 5788 183 30 19414 14.144990892531878 0.9965689886411229 0.05247358477170014 0.049641275617757774

GOTERM_BP_DIRECT GO:0043123~positive regulation of I-kappaB kinase/NF-kappaB signaling 7 2.8925619834710745 0.0113058948320013 7099, 8743, 6366, 7124, 3123, 54106, 958 183 199 19414 3.7317187028036356 0.9999999999433062 0.15639821184268465 0.14795647703479034

GOTERM_BP_DIRECT GO:0046330~positive regulation of JNK cascade 5 2.066115702479339 0.013636068639946148 7099, 6366, 7124, 3265, 54106 183 98 19414 5.41262406601985 0.9999999999995762 0.1768427651743016 0.1672975171263393

KEGG_PATHWAY hsa05132:Salmonella infection 8 3.3057851239669422 0.08040351682336497 7099, 8743, 7124, 3265, 207, 5788, 54106, 7184 128 249 8465 2.1247489959839356 0.9999999989801566 0.1805424423215559 0.11548868780083331

GOTERM_BP_DIRECT GO:0032715~negative regulation of interleukin-6 production 3 1.2396694214876034 0.14640932291489545 7099, 7124, 54106 183 72 19414 4.4203096539162114 1.0 0.7278039905025133 0.6885201124609319

KEGG_PATHWAY hsa05130:Pathogenic Escherichia coli infection 3 1.2396694214876034 0.7998980390472008 7099, 8743, 7124 128 197 8465 1.0070986675126905 1.0 1.0 0.7998980390472008

Annotation Cluster 41 Enrichment Score: 1.3693462721238445

Category Term Count % PValue Genes List Total Pop Hits Pop Total Fold Enrichment Bonferroni Benjamini FDR

UP_KW_BIOLOGICAL_PROCESS KW-0746~Sphingolipid metabolism 4 1.6528925619834711 0.0055863578610937225 6489, 2760, 2583, 2581 125 33 11262 10.920727272727273 0.32059602607841187 0.04818233655193336 0.04399256815611306

GOTERM_BP_DIRECT GO:0006687~glycosphingolipid metabolic process 3 1.2396694214876034 0.019432663421694956 2760, 2583, 2581 183 23 19414 13.837491090520315 1.0 0.2240154255556502 0.21192399053770664

UP_KW_BIOLOGICAL_PROCESS KW-0443~Lipid metabolism 8 3.3057851239669422 0.7182904658181057 55, 6489, 216, 2760, 2583, 2581, 2717, 4758 125 743 11262 0.970078061911171 1.0 1.0 0.926470588235294

Annotation Cluster 42 Enrichment Score: 1.2285716081990756

Category Term Count % PValue Genes List Total Pop Hits Pop Total Fold Enrichment Bonferroni Benjamini FDR

GOTERM_BP_DIRECT GO:0007052~mitotic spindle organization 4 1.6528925619834711 0.01715038637998365 891, 332, 83540, 7272 183 58 19414 7.316374599585453 0.9999999999999998 0.2081114136752402 0.19687841206963685

GOTERM_BP_DIRECT GO:0007094~mitotic spindle assembly checkpoint 3 1.2396694214876034 0.03606727785342407 332, 83540, 7272 183 32 19414 9.945696721311476 1.0 0.33505821910378797 0.31697314896420997

GOTERM_BP_DIRECT GO:0007059~chromosome segregation 4 1.6528925619834711 0.04578063182812878 7153, 332, 83540, 7272 183 85 19414 4.992349726775957 1.0 0.383394964913317 0.3627008752408873

GOTERM_CC_DIRECT GO:0000776~kinetochore 3 1.2396694214876034 0.4301743496478713 332, 83540, 7272 188 161 20624 2.0441390247125675 1.0 1.0 0.9274924471299094

Annotation Cluster 43 Enrichment Score: 0.9806886352421628

Category Term Count % PValue Genes List Total Pop Hits Pop Total Fold Enrichment Bonferroni Benjamini FDR

GOTERM_BP_DIRECT GO:0030593~neutrophil chemotaxis 4 1.6528925619834711 0.04187994907449894 6375, 6366, 6280, 7042 183 82 19414 5.174996667999467 1.0 0.368224128515192 0.34834889844593825

UP_KW_BIOLOGICAL_PROCESS KW-0145~Chemotaxis 4 1.6528925619834711 0.10019471862965433 6375, 6366, 2321, 6280 125 101 11262 3.568158415841584 0.9993141184575609 0.5761196321205124 0.5260222728056851

GOTERM_BP_DIRECT GO:0061844~antimicrobial humoral immune response mediated by antimicrobial peptide 3 1.2396694214876034 0.27232224540731237 6375, 6366, 6280 183 109 19414 2.9198375695593324 1.0 1.0 0.946480231436837

Annotation Cluster 44 Enrichment Score: 0.8205010994390783

Category Term Count % PValue Genes List Total Pop Hits Pop Total Fold Enrichment Bonferroni Benjamini FDR

GOTERM_MF_DIRECT GO:0030246~carbohydrate binding 6 2.479338842975207 0.047912478501208916 2548, 7123, 1116, 4153, 945, 170482 185 202 18945 3.041744715012042 0.9999999998199758 0.5614359660269865 0.545465139859917

INTERPRO IPR018378:C-type lectin, conserved site 3 1.2396694214876034 0.0655682451919441 7123, 4153, 170482 183 44 19144 7.132637853949329 0.999999999999716 0.634819828449277 0.6154473923698389

UP_KW_LIGAND KW-0430~Lectin 5 2.066115702479339 0.09977558356806579 7123, 1116, 4153, 945, 170482 71 173 6858 2.791663274444354 0.941457498609884 1.0 1.0

UP_SEQ_FEATURE DOMAIN:C-type lectin 3 1.2396694214876034 0.19109296177900745 7123, 4153, 170482 188 88 20543 3.7251571566731134 1.0 1.0 0.9808238636363636

INTERPRO IPR001304:C-type lectin 3 1.2396694214876034 0.21093742110104918 7123, 4153, 170482 183 90 19144 3.4870673952641167 1.0 1.0 0.971764705882353

INTERPRO IPR016186:C-type lectin-like 3 1.2396694214876034 0.2670932124418961 7123, 4153, 170482 183 106 19144 2.9607175997525523 1.0 1.0 0.971764705882353

SMART SM00034:CLECT 3 1.2396694214876034 0.27388371025525293 7123, 4153, 170482 122 88 10378 2.899962742175857 0.999999999999999 1.0 0.9719626168224299

INTERPRO IPR016187:C-type lectin fold 3 1.2396694214876034 0.2952550618594366 7123, 4153, 170482 183 114 19144 2.752947943629566 1.0 1.0 0.971764705882353

Annotation Cluster 45 Enrichment Score: 0.8022764644002702

Category Term Count % PValue Genes List Total Pop Hits Pop Total Fold Enrichment Bonferroni Benjamini FDR

KEGG_PATHWAY hsa04115:p53 signaling pathway 6 2.479338842975207 0.004953357517149638 891, 598, 983, 7157, 900, 4193 128 74 8465 5.362119932432433 0.7066883301225847 0.01609841193073632 0.010297769575126878

BIOCARTA h_g2Pathway:Cell Cycle: G2/M Checkpoint 4 1.6528925619834711 0.05948962521726568 891, 983, 7157, 4193 60 25 1623 4.328 0.9999829359261815 0.5324321456945278 0.5056618143467583

BIOCARTA h_EfpPathway:Estrogen-responsive protein Efp controls cell cycle and breast tumors growth 3 1.2396694214876034 0.11255125807610644 891, 983, 7157 60 16 1623 5.071875 0.9999999994780285 0.8394447998176272 0.7972380780390873

GOTERM_BP_DIRECT GO:0065003~macromolecular complex assembly 4 1.6528925619834711 0.16212923581361963 983, 7157, 958, 4193 183 148 19414 2.867227883621326 1.0 0.7766070854426812 0.734689016252522

GOTERM_CC_DIRECT GO:0005759~mitochondrial matrix 6 2.479338842975207 0.28850194848707245 3329, 891, 598, 983, 7157, 217 188 392 20624 1.6791141988710376 1.0 1.0 0.9274924471299094

GOTERM_CC_DIRECT GO:0005813~centrosome 7 2.8925619834710745 0.40919931324822084 891, 9585, 598, 983, 7157, 3397, 30848 188 567 20624 1.3543472550564748 1.0 1.0 0.9274924471299094

UP_KW_BIOLOGICAL_PROCESS KW-0131~Cell cycle 8 3.3057851239669422 0.6353186035428848 891, 9232, 332, 9585, 983, 7157, 900, 83540 125 685 11262 1.0522160583941607 1.0 1.0 0.926470588235294

UP_KW_CELLULAR_COMPONENT KW-0206~Cytoskeleton 10 4.132231404958678 0.9465868520342448 891, 3728, 332, 9585, 598, 983, 4771, 7157, 27, 6280 182 1372 17708 0.7091596450197033 1.0 1.0 0.9465868520342448

Annotation Cluster 46 Enrichment Score: 0.686692701948206

Category Term Count % PValue Genes List Total Pop Hits Pop Total Fold Enrichment Bonferroni Benjamini FDR

UP_KW_BIOLOGICAL_PROCESS KW-0498~Mitosis 7 2.8925619834710745 0.09954772045134813 891, 9232, 332, 9585, 983, 900, 83540 125 289 11262 2.1822560553633217 0.9992792437317786 0.5761196321205124 0.5260222728056851

GOTERM_BP_DIRECT GO:0000086~G2/M transition of mitotic cell cycle 3 1.2396694214876034 0.10283945899623816 891, 332, 983 183 58 19414 5.48728094968909 1.0 0.5977363513086671 0.5654729916235728

GOTERM_BP_DIRECT GO:0051301~cell division 7 2.8925619834710745 0.15562792488706784 891, 9232, 332, 9585, 983, 900, 83540 183 385 19414 1.9288623944361651 1.0 0.756357454328652 0.7155323772757319

GOTERM_CC_DIRECT GO:0030496~midbody 4 1.6528925619834711 0.24092338089115917 332, 9585, 983, 7184 188 187 20624 2.346569575605871 1.0 0.9500647424709354 0.8785237227065578

UP_KW_BIOLOGICAL_PROCESS KW-0132~Cell division 7 2.8925619834710745 0.31095291836653577 891, 9232, 332, 9585, 983, 900, 83540 125 417 11262 1.5124028776978418 0.9999999999930949 1.0 0.926470588235294

UP_KW_BIOLOGICAL_PROCESS KW-0131~Cell cycle 8 3.3057851239669422 0.6353186035428848 891, 9232, 332, 9585, 983, 7157, 900, 83540 125 685 11262 1.0522160583941607 1.0 1.0 0.926470588235294

Annotation Cluster 47 Enrichment Score: 0.6798887538408607

Category Term Count % PValue Genes List Total Pop Hits Pop Total Fold Enrichment Bonferroni Benjamini FDR

KEGG_PATHWAY hsa04062:Chemokine signaling pathway 7 2.8925619834710745 0.06862287534500353 6375, 6366, 1234, 4893, 3265, 207, 3845 128 192 8465 2.411092122395833 0.9999999763399816 0.15840981504874646 0.10133097480850987

GOTERM_BP_DIRECT GO:0070098~chemokine-mediated signaling pathway 3 1.2396694214876034 0.13996664017484417 6375, 6366, 1234 183 70 19414 4.546604215456675 1.0 0.710099702598537 0.6717714294944233

GOTERM_BP_DIRECT GO:0007186~G-protein coupled receptor signaling pathway 6 2.479338842975207 0.9502552756852345 6375, 6366, 367, 1234, 1880, 207 183 961 19414 0.6623564934067997 1.0 1.0 0.9502552756852345

Annotation Cluster 48 Enrichment Score: 0.5762513697440373

Category Term Count % PValue Genes List Total Pop Hits Pop Total Fold Enrichment Bonferroni Benjamini FDR

INTERPRO IPR003961:Fibronectin, type III 5 2.066115702479339 0.1428377210702765 3598, 5788, 3593, 1969, 1438 183 212 19144 2.46726466646046 1.0 1.0 0.971764705882353

UP_SEQ_FEATURE DOMAIN:Fibronectin type-III 1 3 1.2396694214876034 0.36157757613711117 3598, 5788, 1969 188 139 20543 2.3583728761671514 1.0 1.0 0.9808238636363636

UP_SEQ_FEATURE DOMAIN:Fibronectin type-III 2 3 1.2396694214876034 0.36157757613711117 3598, 5788, 1969 188 139 20543 2.3583728761671514 1.0 1.0 0.9808238636363636

Annotation Cluster 49 Enrichment Score: 0.5703835386770084

Category Term Count % PValue Genes List Total Pop Hits Pop Total Fold Enrichment Bonferroni Benjamini FDR

GOTERM_BP_DIRECT GO:0006457~protein folding 5 2.066115702479339 0.09520833565783306 3329, 7265, 7184, 3308, 5903 183 184 19414 2.8828106438583987 1.0 0.5709748453468312 0.5401559621281107

UP_KW_BIOLOGICAL_PROCESS KW-0346~Stress response 3 1.2396694214876034 0.3501900656505586 3329, 7184, 3308 125 112 11262 2.4132857142857143 0.9999999999998791 1.0 0.926470588235294

GOTERM_MF_DIRECT GO:0016887~ATPase activity 5 2.066115702479339 0.5832712753096214 3329, 9585, 4343, 7184, 3308 185 419 18945 1.2220215442172484 1.0 1.0 0.9736842105263158

Annotation Cluster 50 Enrichment Score: 0.4855945428567771

Category Term Count % PValue Genes List Total Pop Hits Pop Total Fold Enrichment Bonferroni Benjamini FDR

GOTERM_BP_DIRECT GO:0032436~positive regulation of proteasomal ubiquitin-dependent protein catabolic process 3 1.2396694214876034 0.16940265327594597 207, 9978, 4193 183 79 19414 4.02863664660718 1.0 0.795272636985493 0.7523470777843483

GOTERM_BP_DIRECT GO:0043161~proteasome-mediated ubiquitin-dependent protein catabolic process 4 1.6528925619834711 0.32434544317369707 207, 9978, 23532, 4193 183 214 19414 1.982942648485777 1.0 1.0 0.946480231436837

GOTERM_BP_DIRECT GO:0016567~protein ubiquitination 5 2.066115702479339 0.635752918379514 54894, 207, 7428, 9978, 4193 183 464 19414 1.143183531185227 1.0 1.0 0.946480231436837

Annotation Cluster 51 Enrichment Score: 0.38358407350971585

Category Term Count % PValue Genes List Total Pop Hits Pop Total Fold Enrichment Bonferroni Benjamini FDR

GOTERM_CC_DIRECT GO:0005634~nucleus 64 26.446280991735538 0.10500094212961619 9585, 6277, 4771, 4255, 8852, 2348, 354, 952, 5698, 9978, 2308, 5272, 55, 7299, 9232, 5077, 4582, 7015, 4100, 4101, 4343, 4102, 23532, 6280, 7490, 3728, 79447, 367, 54763, 80312, 207, 9947, 10983, 728695, 2130, 7184, 4193, 891, 4904, 10953, 332, 2247, 2521, 4105, 6528, 1956, 9014, 4162, 7153, 3791, 7157, 6862, 3397, 2064, 7272, 55553, 983, 900, 7428, 6657, 55635, 51438, 10643, 3308 188 6033 20624 1.1637553738128237 0.9999999999999999 0.5622631094682673 0.5199240198998737

UP_KW_CELLULAR_COMPONENT KW-0963~Cytoplasm 54 22.31404958677686 0.7750822479940116 9585, 6277, 4771, 2346, 598, 5698, 9978, 2308, 5272, 55, 9232, 5153, 4582, 7015, 4100, 4343, 23532, 6280, 7490, 3728, 2875, 367, 207, 3845, 728695, 9447, 27, 3265, 2130, 4193, 3417, 891, 4904, 10953, 332, 1116, 2321, 216, 6528, 8829, 7153, 3791, 7157, 3397, 2064, 55553, 983, 54742, 1485, 7428, 6657, 51438, 10643, 3308 182 5599 17708 0.9383857792502193 1.0 1.0 0.9411764705882353

UP_KW_CELLULAR_COMPONENT KW-0539~Nucleus 53 21.90082644628099 0.8683739633924237 9585, 6277, 4771, 4255, 598, 5698, 9978, 2308, 55, 9232, 5077, 4582, 7015, 4100, 4101, 4343, 23532, 7490, 79447, 367, 80312, 768, 207, 83540, 10983, 5903, 728695, 9447, 3265, 2130, 4193, 891, 4904, 332, 2247, 2521, 54894, 1956, 9014, 7153, 3791, 7157, 6862, 3397, 2064, 55553, 983, 900, 7428, 6657, 55635, 51438, 10643 182 5722 17708 0.9012102891865214 1.0 1.0 0.9411764705882353

Annotation Cluster 52 Enrichment Score: 0.37313174548179157

Category Term Count % PValue Genes List Total Pop Hits Pop Total Fold Enrichment Bonferroni Benjamini FDR

INTERPRO IPR011990:Tetratricopeptide-like helical 4 1.6528925619834711 0.37186705202621606 10953, 7265, 7272, 5903 183 230 19144 1.8193395105725825 1.0 1.0 0.971764705882353

UP_SEQ_FEATURE REPEAT:TPR 3 3 1.2396694214876034 0.3801601745959572 10953, 7265, 5903 188 145 20543 2.260785033015407 1.0 1.0 0.9808238636363636

INTERPRO IPR019734:Tetratricopeptide repeat 3 1.2396694214876034 0.38507916533522535 10953, 7265, 5903 183 140 19144 2.241686182669789 1.0 1.0 0.971764705882353

UP_SEQ_FEATURE REPEAT:TPR 1 3 1.2396694214876034 0.4256896959231804 10953, 7265, 5903 188 159 20543 2.0617221999197106 1.0 1.0 0.9808238636363636

UP_SEQ_FEATURE REPEAT:TPR 2 3 1.2396694214876034 0.4256896959231804 10953, 7265, 5903 188 159 20543 2.0617221999197106 1.0 1.0 0.9808238636363636

SMART SM00028:TPR 3 1.2396694214876034 0.45731552967324834 10953, 7265, 5903 122 132 10378 1.9333084947839045 1.0 1.0 0.9719626168224299

UP_KW_DOMAIN KW-0802~TPR repeat 3 1.2396694214876034 0.5417154412373176 10953, 7265, 5903 149 173 14504 1.6880164487721612 0.9999996356076367 1.0 0.9444444444444444

Annotation Cluster 53 Enrichment Score: 0.3663120226855052

Category Term Count % PValue Genes List Total Pop Hits Pop Total Fold Enrichment Bonferroni Benjamini FDR

UP_SEQ_FEATURE CROSSLNK:Glycyl lysine isopeptide (Lys-Gly) (interchain with G-Cter in SUMO) 6 2.479338842975207 0.0024198614968157514 55553, 367, 7157, 6657, 7490, 5903 188 102 20543 6.427722152690863 0.9670813817778778 0.11170524082225311 0.10948540637014303

GOTERM_MF_DIRECT GO:0000976~transcription regulatory region sequence-specific DNA binding 6 2.479338842975207 0.07841996109293203 55553, 367, 7124, 7157, 6657, 7490 185 234 18945 2.625779625779626 0.9999999999999999 0.7466233795722904 0.7253846401096213

GOTERM_MF_DIRECT GO:0003700~transcription factor activity, sequence-specific DNA binding 8 3.3057851239669422 0.3072333232755652 55553, 5077, 367, 7157, 6862, 6657, 7490, 2308 185 563 18945 1.4551389755652633 1.0 1.0 0.9736842105263158

UP_KW_MOLECULAR_FUNCTION KW-0678~Repressor 9 3.71900826446281 0.3867594917170634 55553, 4904, 332, 7157, 80312, 3397, 2130, 55635, 23532 130 629 11749 1.2931515225632872 0.9999999999999583 1.0 0.967741935483871

GOTERM_MF_DIRECT GO:0043565~sequence-specific DNA binding 5 2.066115702479339 0.42516672386178267 5077, 367, 6657, 7490, 2308 185 342 18945 1.4971550497866288 1.0 1.0 0.9736842105263158

UP_KW_MOLECULAR_FUNCTION KW-0010~Activator 9 3.71900826446281 0.519046698064862 55553, 4904, 367, 7157, 80312, 6862, 2064, 6657, 2308 130 708 11749 1.148859191655802 1.0 1.0 0.967741935483871

GOTERM_BP_DIRECT GO:0006355~regulation of transcription, DNA-templated 10 4.132231404958678 0.6040118278490996 55553, 4904, 5077, 7157, 2521, 2022, 7428, 6657, 2130, 7490 183 1005 19414 1.0555963352635727 1.0 1.0 0.946480231436837

GOTERM_MF_DIRECT GO:0001228~transcriptional activator activity, RNA polymerase II transcription regulatory region sequence-specific binding 5 2.066115702479339 0.6842427209791964 367, 7157, 6657, 7490, 2308 185 477 18945 1.0734319224885263 1.0 1.0 0.9736842105263158

GOTERM_MF_DIRECT GO:0003677~DNA binding 13 5.371900826446281 0.7336672406945086 9014, 7153, 5077, 7015, 7157, 4255, 6862, 55553, 4904, 367, 2521, 80312, 6657 185 1421 18945 0.9368545181353064 1.0 1.0 0.9736842105263158

GOTERM_CC_DIRECT GO:0000785~chromatin 9 3.71900826446281 0.7442710242362944 55553, 5077, 4582, 367, 7157, 6862, 6657, 23532, 2308 188 1053 20624 0.9376250227314057 1.0 1.0 0.9274924471299094

GOTERM_CC_DIRECT GO:0016607~nuclear speck 4 1.6528925619834711 0.7580473434178626 7015, 367, 6657, 7490 188 435 20624 1.0087551968696504 1.0 1.0 0.9274924471299094

GOTERM_BP_DIRECT GO:0006357~regulation of transcription from RNA polymerase II promoter 13 5.371900826446281 0.8964376127212165 5077, 7422, 7124, 7157, 6862, 3265, 7490, 4193, 55553, 367, 983, 2521, 2308 183 1732 19414 0.7962682517447216 1.0 1.0 0.946480231436837

GOTERM_MF_DIRECT GO:0000978~RNA polymerase II core promoter proximal region sequence-specific DNA binding 9 3.71900826446281 0.9107733144710237 55553, 5077, 4582, 367, 7157, 6862, 6657, 7490, 2308 185 1217 18945 0.7573119545181994 1.0 1.0 0.9736842105263158

UP_KW_BIOLOGICAL_PROCESS KW-0805~Transcription regulation 21 8.677685950413224 0.9386219061059229 9014, 5077, 4100, 7157, 4101, 4343, 6862, 3397, 23532, 2064, 2130, 7490, 55553, 4904, 332, 367, 79447, 80312, 6657, 55635, 2308 125 2393 11262 0.7906460509820309 1.0 1.0 0.9386219061059229

UP_KW_BIOLOGICAL_PROCESS KW-0804~Transcription 21 8.677685950413224 0.9558526082893471 9014, 5077, 4100, 7157, 4101, 4343, 6862, 3397, 23532, 2064, 2130, 7490, 55553, 4904, 332, 367, 79447, 80312, 6657, 55635, 2308 125 2462 11262 0.7684874086108855 1.0 1.0 0.9558526082893471

GOTERM_MF_DIRECT GO:0000981~RNA polymerase II transcription factor activity, sequence-specific DNA binding 8 3.3057851239669422 0.9695979034393436 55553, 5077, 367, 7157, 6862, 6657, 7490, 2308 185 1283 18945 0.6385372121927071 1.0 1.0 0.9736842105263158

UP_KW_MOLECULAR_FUNCTION KW-0238~DNA-binding 16 6.6115702479338845 0.9799809525103123 9014, 7153, 5077, 9447, 7015, 7157, 4255, 6862, 7490, 55553, 4904, 367, 2521, 80312, 6657, 2308 130 2095 11749 0.690229484119699 1.0 1.0 0.9799809525103123

Annotation Cluster 54 Enrichment Score: 0.30456012166887764

Category Term Count % PValue Genes List Total Pop Hits Pop Total Fold Enrichment Bonferroni Benjamini FDR

GOTERM_MF_DIRECT GO:0004175~endopeptidase activity 3 1.2396694214876034 0.17196616405021348 354, 5698, 6868 185 77 18945 3.98982098982099 1.0 1.0 0.9736842105263158

UP_KW_PTM KW-0865~Zymogen 3 1.2396694214876034 0.7493984908424736 354, 5698, 6868 172 220 14111 1.1187367864693445 0.999999999999985 0.9466086200115456 0.788840516676288

UP_KW_MOLECULAR_FUNCTION KW-0645~Protease 4 1.6528925619834711 0.9465941518182384 354, 2346, 5698, 6868 130 554 11749 0.65254096084421 1.0 1.0 0.967741935483871

Annotation Cluster 55 Enrichment Score: 0.27112596423375557

Category Term Count % PValue Genes List Total Pop Hits Pop Total Fold Enrichment Bonferroni Benjamini FDR

GOTERM_BP_DIRECT GO:0006281~DNA repair 5 2.066115702479339 0.30023010862578714 9232, 79447, 983, 4255, 9978 183 297 19414 1.7859836985520046 1.0 1.0 0.946480231436837

UP_KW_BIOLOGICAL_PROCESS KW-0227~DNA damage 5 2.066115702479339 0.6846906856868296 9232, 79447, 9447, 4255, 9978 125 420 11262 1.0725714285714285 1.0 1.0 0.926470588235294

UP_KW_BIOLOGICAL_PROCESS KW-0234~DNA repair 4 1.6528925619834711 0.7476071461394089 9232, 79447, 4255, 9978 125 351 11262 1.0267350427350428 1.0 1.0 0.926470588235294

Annotation Cluster 56 Enrichment Score: 0.23873014706847323

Category Term Count % PValue Genes List Total Pop Hits Pop Total Fold Enrichment Bonferroni Benjamini FDR

INTERPRO IPR000742:Epidermal growth factor-like domain 4 1.6528925619834711 0.4125626954883933 4240, 4584, 4585, 3696 183 243 19144 1.7220085902538849 1.0 1.0 0.971764705882353

UP_SEQ_FEATURE DOMAIN:EGF-like 3 1.2396694214876034 0.5722444503142198 4240, 4584, 4585 188 210 20543 1.5610182370820667 1.0 1.0 0.9808238636363636

SMART SM00181:EGF 3 1.2396694214876034 0.653558084985381 4240, 4584, 4585 122 190 10378 1.3431406384814495 1.0 1.0 0.9719626168224299

UP_KW_DOMAIN KW-0245~EGF-like domain 3 1.2396694214876034 0.7189913652645521 4240, 4584, 4585 149 246 14504 1.1871009985267635 0.9999999999664515 1.0 0.9444444444444444

Annotation Cluster 57 Enrichment Score: 0.22314027201375472

Category Term Count % PValue Genes List Total Pop Hits Pop Total Fold Enrichment Bonferroni Benjamini FDR

GOTERM_BP_DIRECT GO:0018107~peptidyl-threonine phosphorylation 3 1.2396694214876034 0.13996664017484417 983, 207, 7272 183 70 19414 4.546604215456675 1.0 0.710099702598537 0.6717714294944233

GOTERM_BP_DIRECT GO:0018105~peptidyl-serine phosphorylation 3 1.2396694214876034 0.5192355537066822 983, 207, 7272 183 185 19414 1.720336730172796 1.0 1.0 0.946480231436837

INTERPRO IPR008271:Serine/threonine-protein kinase, active site 3 1.2396694214876034 0.8053678943875182 983, 207, 7272 183 316 19144 0.9931521062461093 1.0 1.0 0.971764705882353

GOTERM_MF_DIRECT GO:0004674~protein serine/threonine kinase activity 3 1.2396694214876034 0.9016169706206105 983, 207, 7272 185 398 18945 0.7719000407442619 1.0 1.0 0.9736842105263158

SMART SM00220:S_TKc 3 1.2396694214876034 0.9300342297141571 983, 207, 7272 122 365 10378 0.6991690994834941 1.0 1.0 0.9719626168224299

UP_KW_MOLECULAR_FUNCTION KW-0723~Serine/threonine-protein kinase 3 1.2396694214876034 0.9338004623780921 983, 207, 7272 130 394 11749 0.688149160484186 1.0 1.0 0.967741935483871

Annotation Cluster 58 Enrichment Score: 0.17893940817789727

Category Term Count % PValue Genes List Total Pop Hits Pop Total Fold Enrichment Bonferroni Benjamini FDR

UP_KW_BIOLOGICAL_PROCESS KW-0833~Ubl conjugation pathway 10 4.132231404958678 0.43038875106657787 4101, 4102, 54894, 7428, 4105, 9978, 23532, 51438, 4193, 5903 125 741 11262 1.2158704453441294 1.0 1.0 0.926470588235294

GOTERM_MF_DIRECT GO:0004842~ubiquitin-protein transferase activity 4 1.6528925619834711 0.4395661829370552 54894, 7428, 9978, 4193 185 251 18945 1.6319586518789706 1.0 1.0 0.9736842105263158

KEGG_PATHWAY hsa04120:Ubiquitin mediated proteolysis 3 1.2396694214876034 0.6327111796311474 7428, 9978, 4193 128 142 8465 1.3971720950704225 1.0 0.9767478835555838 0.6327111796311474

GOTERM_BP_DIRECT GO:0016567~protein ubiquitination 5 2.066115702479339 0.635752918379514 54894, 207, 7428, 9978, 4193 183 464 19414 1.143183531185227 1.0 1.0 0.946480231436837

GOTERM_BP_DIRECT GO:0006511~ubiquitin-dependent protein catabolic process 3 1.2396694214876034 0.7516331346044482 54894, 9978, 4193 183 286 19414 1.112805227559326 1.0 1.0 0.946480231436837

INTERPRO IPR001841:Zinc finger, RING-type 3 1.2396694214876034 0.7996244260213908 54894, 9978, 4193 183 312 19144 1.0058848255569566 1.0 1.0 0.971764705882353

GOTERM_MF_DIRECT GO:0061630~ubiquitin protein ligase activity 3 1.2396694214876034 0.8535918781456115 54894, 9978, 4193 185 347 18945 0.8853493262715164 1.0 1.0 0.9736842105263158

INTERPRO IPR013083:Zinc finger, RING/FYVE/PHD-type 3 1.2396694214876034 0.948338616224834 54894, 9978, 4193 183 488 19144 0.6431066917495297 1.0 1.0 0.971764705882353

Annotation Cluster 59 Enrichment Score: 0.14413138986144033

Category Term Count % PValue Genes List Total Pop Hits Pop Total Fold Enrichment Bonferroni Benjamini FDR

UP_SEQ_FEATURE DOMAIN:RRM 3 1.2396694214876034 0.64245824580812 2521, 2130, 10643 188 239 20543 1.371605982373364 1.0 1.0 0.9808238636363636

INTERPRO IPR000504:RNA recognition motif domain 3 1.2396694214876034 0.6568961836821372 2521, 2130, 10643 183 235 19144 1.3354726194628532 1.0 1.0 0.971764705882353

SMART SM00360:RRM 3 1.2396694214876034 0.7308903359577898 2521, 2130, 10643 122 220 10378 1.1599850968703427 1.0 1.0 0.9719626168224299

INTERPRO IPR012677:Nucleotide-binding, alpha-beta plait 3 1.2396694214876034 0.7354060754762237 2521, 2130, 10643 183 273 19144 1.149582657779379 1.0 1.0 0.971764705882353

UP_KW_MOLECULAR_FUNCTION KW-0694~RNA-binding 7 2.8925619834710745 0.8387296022903625 4904, 2521, 4343, 2130, 7490, 10643, 5903 130 749 11749 0.8446441409058232 1.0 1.0 0.967741935483871

Annotation Cluster 60 Enrichment Score: 0.08907481961671834

Category Term Count % PValue Genes List Total Pop Hits Pop Total Fold Enrichment Bonferroni Benjamini FDR

GOTERM_MF_DIRECT GO:0046872~metal ion binding 25 10.330578512396695 0.7053523056657108 2130, 3043, 174, 4193, 3417, 332, 2346, 2521, 26872, 54894, 6868, 8829, 170482, 9014, 7153, 5153, 8743, 7015, 7157, 7490, 727897, 780, 1442, 7306, 5903 185 2690 18945 0.9517230985632472 1.0 1.0 0.9736842105263158

UP_KW_LIGAND KW-0862~Zinc 23 9.50413223140496 0.8568054038709093 9014, 5153, 8743, 7157, 4255, 2130, 7490, 6280, 174, 4193, 332, 2346, 367, 2521, 1442, 54894, 80312, 768, 7306, 9978, 1638, 6868, 5903 71 2525 6858 0.8798438153674524 1.0 1.0 1.0

UP_KW_LIGAND KW-0479~Metal-binding 37 15.289256198347106 0.8943082845382898 6277, 27, 4255, 2130, 3043, 174, 4193, 3417, 332, 2346, 2521, 26872, 54894, 7018, 9978, 6868, 1638, 8829, 170482, 9014, 7153, 7299, 5153, 8743, 7015, 7157, 7490, 6280, 727897, 780, 367, 1442, 1001, 80312, 768, 7306, 5903 71 3982 6858 0.8975106288155856 1.0 1.0 1.0

Annotation Cluster 61 Enrichment Score: 0.06436751395592985

Category Term Count % PValue Genes List Total Pop Hits Pop Total Fold Enrichment Bonferroni Benjamini FDR

UP_KW_MOLECULAR_FUNCTION KW-0694~RNA-binding 7 2.8925619834710745 0.8387296022903625 4904, 2521, 4343, 2130, 7490, 10643, 5903 130 749 11749 0.8446441409058232 1.0 1.0 0.967741935483871

GOTERM_MF_DIRECT GO:0003723~RNA binding 12 4.958677685950414 0.8524765016405379 3329, 7153, 4904, 3105, 7015, 2521, 4343, 2130, 7490, 7184, 10643, 5903 185 1468 18945 0.8371014065836954 1.0 1.0 0.9736842105263158

UP_SEQ_FEATURE CROSSLNK:Glycyl lysine isopeptide (Lys-Gly) (interchain with G-Cter in SUMO2) 9 3.71900826446281 0.8965883060524307 3329, 7153, 4904, 10953, 983, 2521, 7490, 10643, 5903 188 1267 20543 0.7761969134662211 1.0 1.0 0.9808238636363636
